# Monograph of *Diplachne* (Poaceae, Chloridoideae, Cynodonteae)

**DOI:** 10.3897/phytokeys.93.21079

**Published:** 2018-01-25

**Authors:** Neil Snow, Paul M. Peterson, Konstantin Romaschenko, Bryan K. Simon

**Affiliations:** 1 Department of Biology, T.M. Sperry Herbarium, Pittsburg State University, Pittsburg, KS 66762, USA; 2 Department of Botany MRC-166, National Museum of Natural History, Smithsonian Institution, Washington, DC 20013-7012, USA; 3 Queensland Herbarium, Mt Coot-tha Road, Toowong, Brisbane, QLD 4066 Australia; 4

**Keywords:** *Azoarcus*, cleistogamy, conservation, leaf anatomy, lectotypification, *Leptochloa*, NAD-ME photosynthesis, nitrogen fixation, plastid DNA sequences, phylogeny, reclamation, stem anatomy, stellate aerenchyma, systematics, typification, weediness

## Abstract

*Diplachne* P. Beauv. comprises two species with C_4_ (NAD-ME) photosynthesis. *Diplachne fusca* has a nearly pantropical-pantemperate distribution with four subspecies: D. fusca subsp. fusca is Paleotropical with native distributions in Africa, southern Asia and Australia; the widespread Australian endemic D. f. subsp. muelleri; and D. f. subsp. fascicularis and D. f. subsp. uninervia occurring in the New World. *Diplachne gigantea* is known from a few widely scattered, older collections in east-central and southern Africa, and although Data Deficient clearly is of conservation concern. A discussion of previous taxonomic treatments is provided, including molecular data supporting *Diplachne* in its newer, restricted sense. Many populations of *Diplachne fusca* are highly tolerant of saline substrates and most prefer seasonally moist to saturated soils, often in disturbed areas. Some populations of *Diplachne fusca* in southern Asia combine nitrogen-fixation, high salinity tolerance and palatibilty to livestock, which should be pursued with further research for purposes of soil reclamation. Diplachne fusca subsp. uninervia is the most invasive of the subspecies and is becoming weedy in some non-native areas, including in the Old World. This monograph provides detailed descriptions of all taxa, a key to the species and subspecies, geographic distributions and information on the anatomy of leaves, stems, lemmatal micromorphology and discussions of the chromosome numbers. Lectotypes are designated for: *Atropis carinata* Grisb.; *Diplachne acuminata* Nash; Diplachne capensis (Nees) Nees var. concinna Nees; Diplachne capensis (Nees) Nees var. obscura Nees, Diplachne capensis (Nees) Nees var. prolifera subvar. minor Nees, *Diplachne halei* Nash, *Diplachne maritima* E.P. Bicknel, *Diplachne muelleri* Benth., *Diplachne reverchonii* Vasey, *Diplachne tectoneticola* Backer, *Leptochloa imbricata* Thurb., *Leptochloa neuroglossa* Peter, Leptochloa uninervia var. typica fo. abbreviata Parodi, *Triodia ambigua* R. Br. and *Triodia parviflora* R. Br.

## Introduction

Recent molecular phylogenetic studies (Peterson et al. 2010, 2012, 2014, 2016; Aliscioni et al. 2012; Soreng et al. 2015, 2017) documented the polyphyly of *Leptochloa* P. Beauv. sensu lato (Parodi 1927; Hitchcock 1951; McNeill 1979; Phillips 1974, 1982; Clayton and Renvoize 1986; Nicora 1995, 2006; Snow 1997a, 2003, 2012). In-depth sampling within and among genera by Peterson et al. (2010, 2012, 2014, 2015) recovered five strongly supported clades among members of *Leptochloa* s.l. The clade bearing the type species of the genus *Diplachne* had strong support levels and merits recognition as a distinct genus, which already is recognised by many authors (Kellogg 2015; Soreng et al. 2017). As re-circumscribed here, *Diplachne* has a narrower circumscription than recognised by many previous authors (e.g. McNeill 1979; Jacobs 1987; Watson and Dallwitz 1992).

**Figure 1.**
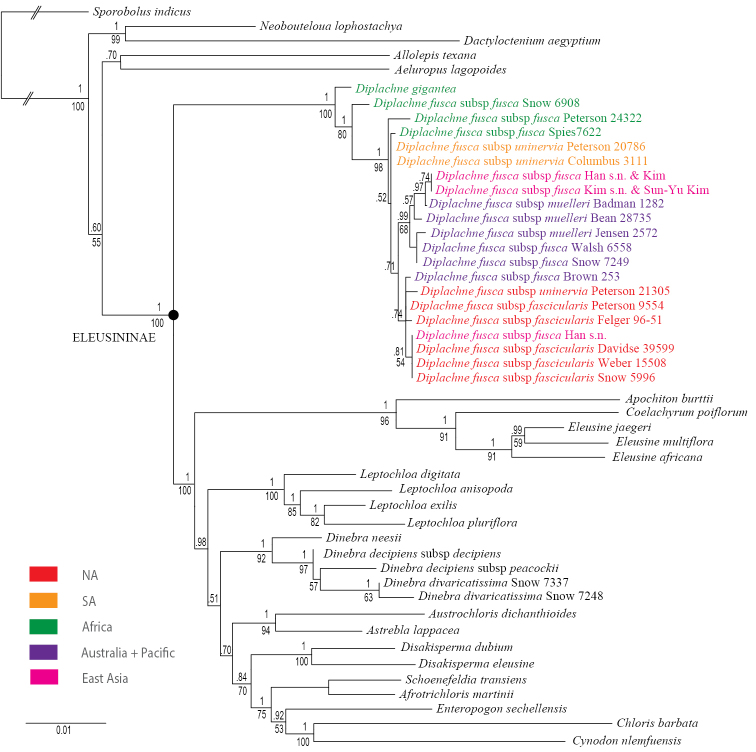
Maximum likelihood tree inferred from nuclear analysis of combined plastid (*rpL32-trnL, ndh*A intron, *rps16* intron, and *rps16-trnK*) and ITS sequences. Numbers above branches are bootstrap values; numbers below branches are posterior probabilities from Bayesian analysis; color indicates native distribution (see legend) of *Diplachne* species. Scale bar: substitutions per site.

The first species now included in *Diplachne* was described by Linnaeus (1762) as *Festuca fusca* L. (= *D. fusca* (L.) P. Beauv. ex Roem. & Schult.). *Diplachne* itself was established by Palisot de Beauvois (1812) in the same publication that he established *Leptochloa* P. Beauv. (type species: *L. virgata* (L.) P. Beauv.) and *Rhabodchloa* P. Beauv., the latter of which has been reduced to synonymy (e.g. Snow 1997a; Peterson et al. 2001; Soreng et al. 2012). Gray (1848), who first reduced *Diplachne* to synonymy under *Leptochloa*, initially did not indicate a taxonomic rank, but later specified its rank as subgenus (Gray 1857). *Diplachne fusca* was transfered to *Uralepis* by Steudel (1855). Hackel (1887, 1900, 1902) was an early advocate of maintaining *Diplachne* as a genus distinct from *Leptochloa*, but his concept of *Diplachne* was broad and included species presently placed by most workers in *Bewsia* Goossens, *Gouinia* E. Fourn. ex Bentham & Hook. f., *Cleistogenes* Keng (=*Kengia* Packer), *Odyssea* Stapf, *Pogonarthria* Stapf and *Trichoneura* Andersson (e.g. Fig. 1 in Valls [1978]). The generic name *Diplachne* R. Br. ex Desf. also once was applied to a species now placed in *Verticordia* DC., an Australian genus of Myrtaceae (Snow 1997a). After Hackel, the generic boundaries of *Diplachne* remained highly unstable (Parodi 1927; McNeill 1979; Phillips 1974, 1982; Clayton and Renvoize 1986; Watson and Dallwitz 1992; Jacobs 1987; Snow 1997a). Synonymy within *Diplachne fusca* is complex and summarised mostly under the subspecies of *D. fusca*.

This paper is the third in an antipated series of five monographic treatments (see Snow and Peterson 2012a, 2013) for species formerly placed in *Leptochloa* P. Beauv. s.l. Subsequent papers will treat *Leptochloa* and *Dinebra* Jacq. The objectives of this monograph are to provide a detailed systematic treatment of *Diplachne* in its newer and more restricted sense; provide complete synonymy (as currently understood); propose many lectotypifications; include a key to species and subspecies; and suggest IUCN recommendations based on current knowledge from herbarium specimens.

## Materials and methods

**Phylogenetic analyses**. Detailed methods for DNA extraction, amplification, sequencing and phylogenetic analysis are given in Peterson et al. (2010, 2012, 2014, 2015, 2016). In brief, the phylogeny was estimated among members of *Diplachne* based on the analysis of five molecular markers (nuclear ITS 1&2 and plastid *rpL32-trnL*, *ndhA* intron, *rps16* intron, and *rps16-trnK* DNA sequences). For this study, a sampling of species was included within subtribe Eleusininae, including the outgroups *Aeluropus* Trin. (Aeluropodinae), *Allolepis* Soderstr. & H.F. Decker (Allolepiinae, see Peterson et al. [2017]), *Dactyloctenium* Willd., *Neobouteloua* Gould (Dactylocteniinae) and *Sporobolus* R. Br. (Sporobolinae). The backbone of this phylogram (Fig. 1) was generated with existing data from Peterson et al. (2010, 2012, 2015, 2016) and the addition of sixteen new samples: thirteen of *Diplachne fusca*, two of *Dinebra divaricatissima* (one of which initially was misidentified as *D. fusca*) and one of *Diplachne gigantea*. Voucher information and GenBank numbers for all samples (including the new ones) are given in Table 1.

**Table 1.** Taxon voucher (collector, number, and where the specimen is housed), country of origin, and GenBank accession for DNA sequences of rps16-trnK, rps16 intron, rpl32-trnL, ndhA intron, and ITS regions (bold indicates new accession); a dash (–) indicates missing data; an asterisk (*) indicates sequences not generated in our lab.

Taxon	Voucher	Country	rps16-trnK	rps16 intron	rpl32-trnL	ndhA intron	ITS
*Aeluropus lagopoides* (L.) Trin. ex Thwaites	Weinert s.n. & Mosawi (US)	Iraq	GU360576	GU360284	GU360013	GU359391	GU359261
*Afrotrichloris martinii* Chiov.	Hemming 3407 (FT)	Somalia, Mudug	KP873645	KP873962	KP873428	KP873827	KP873212
Allolepis texana (Vasey) Soderstr. & H.F. Decker	Hitchcock 7541 (US)	Mexico, Durango	GU360573	GU360318	GU360015	GU359388	GU359264
*Apochiton burttii* C.E. Hubb.	Peterson 24163, Soreng, Romaschenko & Abeid (US)	Tanzania, Dodoma	KP873646	KP873963	KP873429	KP873828	KP873214
*Astrebla lappacea* (Lindl.) Domin	McKinlay s.n. (US)	Australia	GU360568	GU360312	GU360009	GU359395	GU359270
*Austrochloris dichanthioides* (Everist) Lazarides	Anson s.n. (US)	Australia, Queensland	GU360566	GU360310	GU359860	GU359420	GU359272
*Chloris barbata* Sw.	Saarela 1830, Peterson, Soreng & Judziewicz (CAN)	Australia, Northern Territory	KP873659	KP873977	KP873443	KP873838	KP873228
*Coelachyrum poiflorum* Chiov.	Burger 2915 (US)	Ethiopia	GU360601	GU360457	GU359843	–	GU359236
*Cynodon nlemfuensis* Vanderyst	Peterson 24058, Soreng, Romaschenko & Abeid (US)	Tanzania, Mbeya	KP873742	KP874024	KP873542	KP873900	KP873324
*Dactyloctenium aegyptium* (L.) Willd.	Peterson 24110, Soreng, Romaschenko & Abeid (US)	Tanzania, Rukwa	KX582953	KX582889	KX582601	KX582462	KX582328
Dinebra decipiens subsp. decipiens (R. Br.) P. M. Peterson & N. Snow	Snow 7328 & Simon (MO)	Australia, Queensland	JQ345242	JQ345285	JQ345327	JQ345211	JQ345174
Dinebra decipiens subsp. peacockii (Maiden & Betche) P. M. Peterson & N. Snow	Snow 7361 & Simon (MO)	Australia, Queensland	JQ345244	JQ345287	JQ345329	**MF353842**	JQ345176
*Dinebra divaricatissima* (S.T. Blake) P. M. Peterson & N. Snow	Snow 7248, Jacobs & Snow (NSW)	Australia, Queensland	–	–	**MF353850**	–	**MF353830**
*Dinebra divaricatissima* (S.T. Blake) P. M. Peterson & N. Snow	Snow 7337 & Simon (BRI)	Australia, Queensland	–	–	**MF353851**	–	**MF353831**
*Dinebra neesii* (Thwaites) P. M. Peterson & N. Snow	Snow 7380 & Simon (BRI)	Australia, Queensland	JQ345254	JQ345297	JQ345339	JQ345221	JQ345186
*Diplachne fusca* (L.) P. Beauv. ex Roem. & Schult.	Han s.n. (HCCN)	Korea (South), Siheung-si	–	–	–	–	KP057059*
*Diplachne fusca* (L.) P. Beauv. ex Roem. & Schult.	Han s.n. & Kim (HCCN)	Korea (South), Goonsan	–	–	–	–	KP057060*
*Diplachne fusca* (L.) P. Beauv. ex Roem. & Schult.	Kim s.n. & Sun-Yu Kim (HCCN)	Korea (South), Gangwon-do	–	–	–	–	KP057061*
Diplachne fusca subsp. fascicularis (Lam.) P. M. Peterson & N. Snow	Davidse 39599 (US)	USA, Missouri	**MF353870**	**MF353863**	**MF353853**	**MF353844**	**MF353833**
Diplachne fusca subsp. fascicularis (Lam.) P. M. Peterson & N. Snow	Felger 96-51, Schneider, Gillenwater, Tittle & Chavez (US)	Mexico, Sonora	**MF353871**	**MF353864**	**MF353854**	**MF353845**	**MF353834**
Diplachne fusca subsp. fascicularis (Lam.) P. M. Peterson & N. Snow	Peterson 9554 (US)	USA, Louisiana	**MF353872**	**MF353865**	**MF353856**	**MF353846**	**MF353836**
Diplachne fusca subsp. fascicularis (Lam.) P. M. Peterson & N. Snow	Snow 5996 (KSP)	USA, Missouri	**MF353873**	**MF353866**	**MF353857**	**MF353847**	**MF353837**
Diplachne fusca subsp. fascicularis (Lam.) P. M. Peterson & N. Snow	Weber 15508 (US)	USA, Colorado	**MF353875**	**MF353868**	**MF353860**	**MF353849**	**MF353840**
Diplachne fusca subsp. fusca (L.) P. Beauv. ex Roem. & Schult.	Brown 253 (MEL)	Australia, Queensland	KP873752	–	KP873550	KP873909	KP873334
Diplachne fusca subsp. fusca (L.) P. Beauv. ex Roem. & Schult.	Peterson 24322, Soreng, Romaschenko & Mbago (US)	Tanzania, Arusha	KP873754	KP874035	KP873553	KP873911	KP873337
Diplachne fusca subsp. fusca (L.) P. Beauv. ex Roem. & Schult.	Snow 6908 (KSP)	Botswana	–	–	**MF353858**	–	**MF353838**
Diplachne fusca subsp. fusca (L.) P. Beauv. ex Roem. & Schult.	Snow 7249 (KSP)	Australia, Queensland	**MF353874**	**MF353867**	**MF353859**	**MF353848**	**MF353839**
Diplachne fusca subsp. fusca (L.) P. Beauv. ex Roem. & Schult.	Walsh 6558 (MEL)	Australia, Western Australia	KP873755	KP874036	KP873554	KP873912	KP873338
Diplachne fusca subsp. fusca (J. Presl) P. M. Peterson & N. Snow	Spies 7622 (BLFU)	South Africa, Free State	–	–	–	–	DQ655804*
Diplachne fusca subsp. muelleri (Benth.) P. M. Peterson & N. Snow	Badman 1282 (MO)	Australia, South Australia	JQ345249	JQ345292	JQ345334	JQ345216	JQ345181
Diplachne fusca subsp. muelleri (Benth.) P. M. Peterson & N. Snow	Bean 28735 (BRI)	Australia, Queensland	**MF353869**	**MF353862**	**MF353852**	**MF353843**	**MF353832**
Diplachne fusca subsp. muelleri (Benth.) P. M. Peterson & N. Snow	Jensen 2572 (BRI)	Australia, Queensland	–	–	**MF353855**	–	**MF353835**
Diplachne fusca subsp. uninervia (J. Presl) P. M. Peterson & N. Snow	Columbus 3111 (RSA)	Argentina, Mendoza	–	–	–	–	EF153059*
Diplachne fusca subsp. uninervia (J. Presl) P. M. Peterson & N. Snow	Peterson 20786, Soreng, Romaschenko & Gonzalez-Elizondo (US)	Peru, Arequipa	JQ345250	JQ345293	JQ345335	JQ345217	JQ345182
Diplachne fusca subsp. uninervia (J. Presl) P. M. Peterson & N. Snow	Peterson 21305, Saarela & Flores Villegas (US)	Mexico, Queretaro	GU360694	GU360391	GU359809	GU359461	GU359147
*Diplachne gigantea* Launert	Browning 8 (P)	Botswana	–	–	**MF353861**	–	**MF353841**
*Disakisperma dubium* (Kunth) P. M. Peterson & N. Snow	Peterson 22334 & Saarela (US)	Mexico, Oaxaca	GU360695	GU360416	GU359811	GU359442	GU359145
*Disakisperma eleusine* (Nees) P. M. Peterson & N. Snow	Snow 6982 (MO)	South Africa,	JQ345248	JQ345291	JQ345333	JQ345215	JQ345180
*Eleusine africana* Kenn.-O'Byrne	Peterson 24072, Soreng, Romaschenko & Abeid (US)	Tanzania, Rukwa	KP873757	KP874038	KP873563	KP873914	KP873347
*Eleusine jaegeri* Pilg.	Peterson 24299, Soreng, Romaschenko & Mbago (US)	Tanzania, Arusha	KP873762	KP874043	KP873574	KP873918	KP873358
*Eleusine multiflora* Hochst. ex A. Rich.	Peterson 24272, Soreng, Romaschenko & Mbago (US)	Tanzania, Shinyanga	KP873763	KP874044	KP873576	KP873919	KP873360
*Enteropogon sechellensis* (Baker) T. Durand & Schinz	Peterson 23815, Soreng & Romaschenko (US)	Tanzania, Dar Es Salaam	KP873787	KP874064	KP873602	KP873934	KP873386
*Leptochloa anisopoda* (Scribn. ex B.L. Rob.) P.M. Peterson	Proosdij 813, van de Riet & Zauder (US)	Aruba	KP873777	–	KP873593	KP873928	KP873377
*Leptochloa digitata* (R.Br.) Domin	Risler 476, Kerrigan (MO)	Australia, Northern Territory	JQ345246	JQ345289	JQ345331	JQ345213	JQ345178
*Leptochloa exilis* (Renvoize) P.M. Peterson	Anderson 37049, Stieber & Kirkbride (US)	Brazil, Bahia State	KP873676	–	KP873464	–	KP873250
*Leptochloa pluriflora* (E. Fourn.) P. M. Peterson & N. Snow	Peterson 15048 & Refulio-Rodriguez (US)	Peru, Cajamarca	GU360623	GU360334	GU359905	GU359554	GU359212
*Neobouteloua lophostachya* (Griseb.) Gould	Peterson 11515 & Annable (US)	Argentina, San Juan	GU360725	GU360273	GU360004	GU359396	GU359123
*Schoenefeldia transiens* (Pilg.) Chiov.	Peterson 24216, Soreng, Romaschenko & Mbago (US)	Tanzania, Tanga	KP873815	KP874088	KP873632	KP873954	KP873415
*Sporobolus indicus* (L.) R. Br.	Peterson 22025 & Saarela (US)	Mexico, Chihuahua	GU360630	GU360355	GU359913	GU359504	GU359209

**Morphology**. Morphological characters from approximately 2200 specimens from over 80 herbaria were examined by the first author between 1986 and 2017, representing the entire geographic range of *Diplachne* (see Acknowledgements; abbreviations of herbaria following Thiers [2017]). Given the wide distribution of *Diplachne fusca* and its representation in virtually every herbarium the first author has visited, it is estimated that less than half of the specimens of *D. fusca* globally are cited here, particularly those from eastern Asia, middle eastern and west African herbaria, as well as more regionally-focused herbaria worldwide. The first author has collected specimens of *D. fusca* from North America (including Mexico), southern Africa (Botswana, South Africa, Namibia) and Australia (New South Wales and Queensland) and grown specimens from these continents in a greenhouse. The second author has collected members of *Diplachne* from North America, South America and Africa. The fourth author collected specimens from Africa and Australia, including the rare *Diplachne gigantea*. Geocoordinates are excluded in specimens examined given that most lacked such data at the time of study or were examined before reliable geocoordinates had been added or updated.

**Type specimens**. Details of types are indicated for those that have been confirmed (including also in Excluded Names); those that merely indicate “type” have not been confirmed or seen. Barcode numbers are enclosed by square brackets; accession numbers are indicated with a hyphen (–) following the herbarium’s acronym.

**Species concept.** A general lineage species concept (de Queiroz 1998) a diagnosability criterion were used based primarily on gross mophology (Snow 1997b; Snow et al. 2003). However, the molecular data also tested the monophyly of each taxon (Peterson et al. 2014, 2015; Fig. 1).

**Anatomy.** Numerous fresh samples of leaves and stems were collected in the field for *Diplachne fusca*, including samples of all subspecies. Samples were placed in 70% ethanol with a trace of glycerin to soften tissues. No specimens of *D. gigantea* were located in the field during this project (see comments under that species). Leaves were sectioned by a rotary microtome at Rancho Santa Ana Botanic Garden in the laboratory of Dr. J. Travis Columbus or by hand by the first author at the Missouri Botanical Garden. Data from lemmatal micromorphology (Snow 1996) and caryopsis morphology (Snow 1998b) are included from those publications.

Descriptive terminology for leaf anatomy largely follows Ellis (1976) and includes information from Snow (1997a). This includes *keel* to refer to the proliferation of parenchyma (abaxially, but usually more prominent adaxially) that surrounds the central-most (median) vascular bundle, which typically extends laterally to at least the first vascular bundle on either side of the median bundle. *Lacunae* in the keel refer to areas of the keel in which parenchyma disintegrates during laminar ontogeny. *Projection*, in association with primary and secondary vascular bundles, is a term relative to the horizontal level of adjacent adjacent intercostal zones.

**Geographical distributions.** Abbreviations follow Brummitt (2001) for both native and non-native distributions. Given the large number of specimens cited and to assist readers, countries are offset in bold and first-level divisions (states, provinces etc.) are underlined.

## Results and discussion

**Phylogeny.** A total of 46 new sequences from four species are newly reported in GenBank (Table 1). The total aligned characters for individual regions and other parameters are noted in Table 2. The plastid–ITS sequences were combined in the analysis since there was little incongruence among between these data sets (Figure 1).

**Table 2.** Characteristics of the five regions, *rpL32-trnL, ndhA* intron, *rps16* intron, *rps16-trnK* and ITS, and parameters used in Bayesian analyses indicated by Akaike Information Criterion (AIC).

	*rpL32-trnL*	*ndhA intron*	*rps16 intron*	*rps16-trnK*	Combined plastid data	*ITS*	Overall
Total aligned characters	813	1088	812	771	3484	718	4202
Sequencing success (%)	89.8	79.6	77.6	83.7	82.7	95.9	85.3
Number of new sequences	12 (27.3%)	10 (25.6%)	9 (23.7%)	9 (22%)	40 (24.7%)	10 (21.3%)	50 (23.9%)
Likelihood score (-lnL)	2537.68	2878.00	2086.42	2311.14		5378.00	
Number of substitution types	6	6	6	6	-	6	-
Model for among-site rate variation	gamma	gamma	gamma	gamma	-	gamma	-
Substitution rates	0.8794 1.8895 0.2840 0.8677 1.74818 1.0000	1.1091 3.1362 0.6149 2.9448 2.8808 1.0000	1.3551 1.2588 0.1754 0.7767 1.7467 1.0000	1.1364 3.1297 0.4521 1.7450 2.7943 1.0000	-	0.8345 2.1975 1.8367 0.5972 4.1384 1.0000	-
Character state frequencies	0.3663	0.3609	0.3776	0.3050	-	0.2325	-
	0.1354	0.1401	0.1157	0.1555		0.2574	
	0.1359	0.1482	0.1740	0.1470		0.2626	
	0.3623	0.3507	0.3325	0.3923		0.2474	
Proportion of invariable sites	0.2868	0.3418	1.0051	0.1261	-	0.2326	-
Substitution model	TVM+G	TVM+G	TVM+G	TVM+I	-	GTR+I+G	-
Gamma shape parameter (α)	0.8078	1.1794	0.2996	1.5741	-	0.8378	-

The maximum-likelihood tree from the combined analysis of ITS and four plastid regions *(rpL32-trnL, ndhA* intron, *rps16* intron and *rps16-trnK*) is well resolved with strong support for the monophyly of *Diplachne* (Fig. 1; posterior probability (PP) = 1, bootstrap (BS) = 100].

Within *Diplachne*, *D. gigantea* is sister to the numerous accessions of *D. fusca*. Among the accessions of *D. fusca*, the most strongly supported clade is an east Asian-Australian clade (PP = 99; BS = 68). However, genetic variation among DNA sequences separates only some of accessions of *D. fusca* unequivocally. The basal-most accession of D. fusca subsp. fusca (Snow 6908 from Botswana) has strong support (PP = 1, BS = 80) and the remainng accessions of *D. fusca* also are well supported (PP = 1, BS = 98). The three earliest-arising accessions of *D. fusca* are African (Snow 6908; Peterson 24322; Spies 7622 [Table 1]) which, with the basal-most (and African) *D. gigantea*, suggest an African origin of the genus.

None of the accessions of *D. fusca* cluster exclusively by subspecies or broad geographical area (e.g. continents or Asia+Pacific). For example, one of the three accessions from South Korea (Han s.n.) forms a polytomy with North American accessions of subsp. fascicularis (Davidse 39599 and Snow 5996, both from Missouri; Weber 15508, from Colorado), which collectively are part of a larger clade that includes another polytomy with two North American accessions of subsp. fusca (Felger 96–51 from Sonora, Mexico; Peterson 9554 from Louisiana), an accession of subsp. *uninervia* from Australia (Peterson 21305) and another accession of subsp. fusca from Australia (Brown 253). Two other accessions of subsp. fusca from South Korea (Han s.n. & Kim; Kim s.n. & Sun-yu Kim) form a clade embedded with two accessions of subsp. muelleri (Badman 1282; Bean 28735). A similar pattern exists for a subclade within the *fusca* clade overall, wherein an accession of subsp. muelleri (Jensen 2572) is sister to two accessions of subsp. fusca from Australia (Snow 7249; Walsh 6558). The polyphyletic nature of accessions of Diplachne fusca subsp. fusca is not surprising, as it is the most geographically widespread and morphologically diverse of the four subspecies.

The location of some accessions (Peterson 20786, Columbus 3111) of the amphitropical (Snow 2003, 2012) D. fusca subsp. uninervia near the base of the *fusca* clade suggests the possibility of a South American origin of this subspecies, but that interpretation needs further testing and does not explain the presence of one accession from Estado de México, México (Peterson 21305) in a large polytomy elsewhere. Of all subspecies, D. f. subsp. uninervia has the greatest weedy tendencies and is now distributed across many non-native areas (e.g. Snow and Simon 1999; Pérez et al. 2010). The significant separation of the Mexican accession from the South American accessions also suggests that subsp. *uninervia* may not have had a singular origin.

The three accessions of the exclusively Australian D. f. subsp. muelleri likewise are not monophyletic, given that one (Badman 1282 from South Australia) forms a clade containing two accessions of D. f. subsp. fusca from Korea and another clade includes an accession (Jensen 2527) with two accessions of subsp. fusca (Snow 7249; Walsh 6558). The Korean sequences were obtained from GenBank, but vouchers have not been seen.

The molecular data may accurately reflect the true evolutionary relationships of the specimens sampled and thus support the lack of monophyly among the subspecies. Given the widespread geographical distribution of D. fusca and of subsp. fusca in particular, the lack of monophyly would not be surprising. If accurately reflecting the actual history, then the molecular data suggest that the morphological characters used to separate the subspecies may be inadequate. The frequent inability of morphological characters, by themselves, to accurately recover genera within Poaceae, is well known (Kellogg 2015). In contrast, if the subspecies are monophyletic as recognised herein (largely) by morphological data, then any of several factors may have contributed to the incongruity of geographical region of origin and clade placement among subspecies of *D. fusca*, including: multiple mutational hits among base pairs, lineage sorting, hybridisation and long-distance dispersal. Among long-distance dispersal events, some of these might include human-mediated dispersal events within the last few millennia. In the authors’ view, a more in-depth sampling of populations is necessary to test further hypotheses of monophyly among the subspecies. Another possibility, of course, is that the taxonomy at the subspecific level needs adjusting, but that would require extensive re-analysis of the thousands of herbarium specimens that underlie the morphologically-based taxa. A high sampling priority in the authors’ view is to obtain DNA samples of D. fusca subsp. fusca from South America, to see where they fit into the phylogenetic framework.

Elsewhere in Figure 1, two accessions of *Dinebra divaricatissima* formed a clade that rendered *D. decipiens* paraphyletic. If further sampling upholds this arrangement, then *D. divaricatissima* might be better treated as a subspecies of *D. decipiens*. An earlier placement (Peterson et al. 2015) of *Dinebra divaricatissima* within *Diplachne* was based on a misidentified individual of *Diplachne fusca*.

**Morphology.** The elongate, broadly to narrowly acute membranous ligule of *Diplachne* and numerous, dorsally rounded or flattened florets (before seed development), most consistently diagnose the genus from species formerly placed in *Leptochloa* sensu lato (Snow 1997a, 2003). As reported previously (Peterson et al. 2012), banding patterns from chloroplast DNA restriction site analysis of populations of *D. fusca subsp. fascicularis* and *uninervia* were virtually identical, but consistently different from other North American members of *Leptochloa* s.l. (Snow unpublished, 1991). More recently, Peterson et al. (2014) demonstrated that DNA barcoding generally can separate members of *Leptochloa* s.l. with relatively high levels of confidence.

Geographically localised morphological variation among populations of *Diplachne fusca* is widespread, which has led to numerous heterotypic synonyms. After observing this variation globally, particularly with regards to taxa here included in Diplachne fusca subsp. fusca, Snow (1997a) concluded that only four infraspecific taxa could be consistently diagnosed morphologically following a phylogenetic species concept or (later) a general lineage concept using a diagnosability criterion (Snow 1997b; Snow et al. 2003). Although some authors might choose to re-elevate the subspecies of *D. fusca* to the specific level, it should be stressed that the nominative taxon, which is the most widespread and exhibits the highest levels of morphological variation, intergrades into each of the other three to some degree with virtually all morphological characters. Whether these taxa hybridise has not been tested. A collection from Orange County, California (Beetle 13066 [OKLA]) may be a hybid between *L. f.* subspp. *fascicularis* and *uninervia*. *Diplachne gigantea* is a rare, evidently emergent aquatic species endemic to a few narrow regions of southern Africa.

**Leaf anatomy.** Detailed anatomical descriptions follow each taxon. In general, leaves show C_4_ (NAD-ME) anatomy; primary vascular bundles project considerably more than secondary bundles and a prominent parenchymatous keel is present in the midnerve region, which typically develops a single (often prominent) lacuna.

Sutton (1973a) commented briefly on leaf anatomy of D. fusca subsp. fascicularis (as *Leptochloa fascicularias* [sic]) but not in a comparative context with other taxa. Earlier workers also made limited observations of leaf anatomy. Hattersley and Watson (1976) used the presence of an “intervening cell” to predict a XyMS+ anatomy for *D. fusca* (as *Diplachne parviflora* (R. Br.) Benth.). Clifford and Watson (1977) illustrated the transverse section of *Diplachne parviflora*. Ellis (1977) reported NAD anatomy in *Diplachne fusca*. However, Valls (1978) carried out the most extenstive investigation of species formerly placed in *Leptochloa* s.l., which now inludes that genus, *Disakisperma* Steud., *Dinebra*, *Trigonochloa* P.M. Peterson & N. Snow and *Diplachne* (Peterson et al. 2012; 2014, 2015). Watson and Dallwitz (1992: 324) briefly mentioned leaf anatomy in *Diplachne*.

Valls (1978) studied cross sections of all of subspecies of *D. fusca* (under various names). Based on the prominent adaxial projection of the primary vascular bundles of *D. fusca*, Valls (1978) reported them as being “nodular” in transverse section, meaning that the primary (and to some degree secondary) vascular bundles project from the adjacent tissues. He also noted the broad and prominent parenchyma midnerve region of the adaxial surface among subspecies of *D. fusca* (Valls 1978), which is simply referred to as the keel. Most specimens of *D. fusca* have prominent keels on the abaxial surface, but these become increasingly narrow distally.

Whereas Snow (1997a) did not examine cellular detail of leaf surfaces, Valls (1978) observed saddle-shaped silica bodies for *Diplachne fusca subsp. fascicularis*, *fusca* and *uninervia*. All three species of *Disakisperma* (Snow and Peterson 2013) also had saddle-shaped silica bodies on the laminar surface (Valls 1978), as did *Leptochloa malayana* (C.E. Hubb.) Jansen ex Veldkamp and *L. longa* Griseb., species not included in molecular studies (Peterson et al. 2012) and which presently are *incertae sedis* at the generic level.

**Stem anatomy.** Comparative studies of stem anatomy in Poaceae have been limited (but see de Wet 1960; Ebinger and Carlen 1975; Sánchez 1979, 1981a-b; Sánchez et al. 1989; Clark and Fisher 1987). The internodes of *D. fusca* are hollow, whereas stellate aerenchyma comprises the pith of *D. gigantea*. Stellate aerenchyma is known from some grasses (Pohl and Lersten 1975) and is not uncommon among plants that remain submerged to somewheat emergent in water, which appears to be the preferred, if not exclusive, habitat of *D. gigantea*.

**Lemmatal micromophology.** All examined specimens of *Diplachne* share a combination of micromorphological characters including the presence of long and short cells, silica cells, cork cells and bicellular microhairs (Snow 1996). All members of *Diplachne fusca* have macrohairs with obtuse or rounded apices, but these were absent or scant for *D. gigantea* and, if present, then restricted to the base of the lemma (Snow 1996, 1997a). *Diplachne fusca* and *D. gigantea* have papillate short cells (Snow 1996).

**Reproductive biology.** Hackel (1906) believed that some specimens of Diplachne fusca subsp. fascicularis approach a cleistogamous condition, which he defined strictly to be plants that showed intrastigmatic anthers crushed at the top of a mature fruit. In contrast, for D. fusca subsp. uninervia Valls (1978: 96) observed flowering in the first two panicles in a greenhouse-grown specimen (Valls 3514 [TAES]) that produced numerous seeds. Anthesis was not observed despite a daily check, suggesting complete cleistogamy during this interval. However, the next two inflorescences to emerge were observed to undergo anthesis (by virtue of exserted stamens) and production of seed, suggesting the possibility of seasonable shifts in chasmogamy and cleistogamy. Valls (1978) also reported observing crushed anthers in D. fusca subsp. muelleri, which also suggests the possibility of cleistogamy. In contrast, he reported no evidence of cleistogamy for D. fusca subsp. fusca. Given that anthers tend to be significantly shorter in the predominantly Neotropical subspecies of *D. fusca* (*fascicularis* and *uninervia*) compared to the native Paleotropical subspecies (*fusca* and *muelleri*), Valls (1978) suggested that predominately chasmogamous and predominantly cleistogamous lineages appear to co-exist in *D. fusca*, which he used also as evidence that the three subspecies he studied (all excluding D. f. subsp. fusca) could be treated conspecifically, as in the present paper. Further, he posited that cleistogamous lines were developed from polymorphic chasmogamous stock in D. fusca subsp. fusca (Valls 1978: 97).

**Caryopsis morphology.** The caryopsis of *Diplachne* is flattened dorsally, with the pericarps of *D. fusca* and *D. gigantea* easily separable when soaked in water at room temperature (Snow 1998b). Dorsal flattening and readily separable pericarps also occur in species now treated in *Disakisperma* and among other genera of Chloridoideae (Snow and Peterson 2013), but most caryopses of *Disakisperma* also are broadly concave at maturity. A slightly concave surface has been noted previously (Valls 1978) for some specimens of *D. fusca*, although the caryopses of Diplachne are generally elliptic in transverse section and, if concave, then notably less so than the normal condition in *Disakisperma* (Snow and Peterson 2013).

**Embryo formula.** Reeder (1957, 1961) took the results of previous studies of grass embryos and those of his own to create an “embryo formula”, which typified variation in a few observable characters of grass embryos. Reeder used the letters “F” and “P” to represent the condition typically found in subfamilies Festucoideae and Panicoideae in their former, much broader circumscriptions (e.g. Hitchock 1951). In addition, Reeder (op. cit.) indicated presence (+) or absence (–) of an epiblast. The typical formula for genera in subfamily Chloridoideae was P+PF, indicating (in order): an elongation of the vascular system between the point of divergence of the scutellum and coleoptile (P); epiblast present (+); presence of a cleft between the lower part of the scutellum and the coleorhiza (second P); and embryonic leaf in cross section with non-overlapping margins and relatively few vascular bundles (F). According to Valls (1978: 109), Reeder based his summary of *Leptochloa* (i.e. including the species treated here as *Diplachne*) on four species, but did not indicate specifically which species were examined. Valls (1978: 110 [voucher: Valls 3468 {TAES}) confirmed the formula for D. fusca subsp. fusca as P+PF.

**Pollen.** The pollen of *Diplachne* has been studied minimally, but Liu et al. (2004) report “type 4” pollen (for Chloridoid grasses) for *D. fusca*, which has an annulate aperture, an insular exine and a “sculptural density” of 0.5–2 µm^2^. These authors also showed that *Dinebra* (=*Leptochloa*) *panicea* (Retz.) P.M. Peterson & N. Snow has two aperatures, in contrast with the single aperture of *D. fusca*, the latter of which has also a minute annulus surrounding the aperture. Further study may reveal characters of pollen that reliably diagnose *Diplachne* from related genera.

**Fungal infections.** The fungal genera *Puccinia* Pers. and *Ustilago* (Pers.) Roussel have been reported for species included in *Diplachne* (Watson and Dallwitz 1992). The Australian *Ustilago serena* Syd. was first collected on *Diplachne* in 1936 (Shivas and Vánky 2003; see also *U. serena* on *Diplachne parviflora* [= D. fusca subsp. fusca); http://www.padil.gov.au/aus-smuts/pest/main/140235).

**Etymology.** From the Greek *diploos* (=double) and *achne* (=awn), referring to the 2-toothed condition of some specimens of the lemma.

### Key to *Diplachne* and morphologically similar genera

A recurring and emergent theme from DNA sequencing studies in Poaceae is that morphological characters alone frequently are unreliable indicators of phylogenetic relationships (Kellogg 2015). A corollary is that generic keys are not always easily written using morphological characters. The following key is modified slightly from Peterson et al. (2012) for *Diplachne* and related genera.

**Table:** 

1	Ligules (1.5–)4–8(–15) mm long, apex acute to attenuate, becoming lacerate by tearing with age; spikelets sometimes quite distant along primary branches; spikelets and lemmas somewhat dorsally rounded or flattened; nearly worldwide in tropical and warm-temperate regions below 2000 metres, often of low-lying, seasonally mesic and disturbed soils	***Diplachne***
–	Ligules 0.2–7 mm long, apex usually truncate to obtuse and somewhat erose; spikelets rarely if ever distant along primary branches; spikelets and lemmas somewhat to distinctly laterally compressed; distribution various	**2**
2	Apex of lemmatal hairs mostly clavicorniculate (obtuse to somewhat acute in *D. dubium*); base of lemma often indurate and sometimes 5-veined; plants perennial; ligules 0.8–2.2 mm long, apex erose	***Disakisperma***
–	Apex of lemmatal hairs acute to obtuse, never clavicorniculate; base of lemma soft and almost always 3-veined; plants annual or perennial; ligules (0.2–) 0.5–5.5(–7.0) mm long, apex usually entire	**3**
3	Panicle branches usually with 2 or more branches per node; lemmas 1- to 3-awned or unawned (the awns often >2 mm); plants perennial; culms solid; ligules 0.5–3.0 mm long, apex ciliate	***Leptochloa***
–	Panicle branches usually with a single branch per node; lemmas unawned or shortly awned (awns typically < 2 mm); plants annual or perennial; culms hollow or sometimes solid; ligules (0.2–)0.5–5.5(–7.0) mm long, apex membranous	***Dinebra***

### Taxonomic treatment

#### Diplachne

Taxon classificationPlantaePoalesPoaceae

Beauv. , Syn. Pl. Glumac. 1: 287. 1854.

Diplachne P. Beauv., Ess. Agrostogr. 80. 1812. Leptochloa subg. Diplachne (P. Beauv.) A. Gray, Man., rev. ed., 555. 1857. Diplachne P. Beauv. sect. Eudiplachne Asch. and Graebn., Syn. Mitteleur. Fl., 2: 339. 1900. Type: Diplachne fascicularis (Lam.). P. Beauv. [= Diplachne fusca subsp. fascicularis (Lam.) P.M. Peterson & N. Snow].

##### Type species.

*Diplachne fusca*

##### Description.

Plants cespitose, annual or perennial, arising from fibrous roots or sometimes rhizomatous. Culms (3–)10–250(–300) cm tall, round or somewhat compressed, mostly ascending to erect, often geniculate at lower nodes (rarely decumbent and rooting at nodes; sometimes highly reduced and prostrate), often branching. Internodes hollow or with stellate arenchyma; nodes glabrous. Leaves cauline, midrib (“keel”) prominent proximally on upper surface, blades flat or becoming involute when dry, apex attenuate; sheaths open, longer or shorter than internodes. Ligules (1.5–)4–8(–15) mm, membranous or hyaline, acute to attenutate but becoming lacerate in age. Inflorescence a panicle of spicate primary branches, terminal or sometimes lateral, completely exserted or partially enclosed basally in sheath. Panicle branches numerous, steeply erect to divergent or even reflexed (in age) but mostly ascending, stiff, minutely scabrous, whorled, subwhorled or (mostly) alternate along the rachis, typically one-sided throughout their length with spikelets in 2 rows, each branch terminating in a functional spikelet. Spikelets rounded to somewhat compressed, becoming more rounded or flattened in maturity (with caryopsis), distant to tightly imbricate, green to plumbeous (lead-coloured) in flower; callous glabrous; florets mostly (2–)4–12(–20), perfect (occasionally male or female); rachilla only rarely prolonged. Glumes unequal, membranous, 1-nerved. Lemmas 3-nerved, glabrous to serious on midvein and lateral nerves, especially proximally; apex acute, obtuse, sometimes emarginate, awnless, mucronate or awned; lateral nerves distinct and occasionally excurrent. Palea membranous, typically subequal to lemma. Stamens 1, 2, or (mostly) 3, anthers 0.2–2.7 mm long, yellow or maroon. Caryopsis elliptic to narrowly elliptic in hilar profile; dorsally compressed. *n*=10; 2*n*=19, 20.

##### Vernacular name.

Given unstable generic boundaries, no common name has been applied consistently. For English, “diplachne” is simply recommended given its shortness and ease of pronunciation.

##### Key to the species of *Diplachne*

**Table:** 

1	Plants erect or geniculate, to 170 cm tall, sometimes rooting at the lower nodes; culms hollow; lemmas more or less hairy on the nerves; sheaths glabrous; panicle branches 3–25 cm long, relatively stiff, mostly erect to ascending, much less frequently divergent to reflexed; florets sometimes tightly imbricate entirely or mostly concealing the rachilla	***Diplachne fusca***
–	Plants mostly erect, to 300 cm tall, sometimes rhizomatous; culms with abundant stellate aerenchyma; lemmas glabrous or with short hairs near the base of the nerves; lower sheaths pubescent, but becoming glabrous with age; panicle branches 12–18 cm long, somewhat lax, probably divergent to reflexed when fresh (not confirmed in field); florets less tightly imbricate and sometimes revealing rachilla	***Diplachne gigantea***

#### Diplachne fusca

Taxon classificationPlantaePoalesPoaceae

(L.) P. Beauv. ex Roem. & Schult., Systema Vegitabilum 2. 1825.

Festuca fusca L., Syst. Nat., ed. 10, 2: 876. 1759. Leptochloa fusca (L.) Kunth, Révis. Gramin. 1: 91. 1829. Diplachne fusca (L.) P. Beauv. ex Stapf, Fl. Cap. 7: 591. 1900, nom. illeg. hom. Diplachne fusca (L.) P. Beauv. ex Stuck., Anales Mus. Nac. Buenos Aires 11: 128. 1904, nom. illeg. hom. Uralepis fusca (L.) Steud., Syn. Pl. Glumac. 1: 247. 1855.

##### Type.

Palestine, F Hasselquist s.n. (lectotype: LINN 92.21!; designated by Phillips, Fl. Trop. East Afr. Gram. (2): 281. 1974)

##### Description.

Annuals or perennials. Culms 3 cm long (when prostrate) to 170 cm tall, 1–8 mm wide at base, round or sometimes flattened, ascending to erect (sometimes completely prostrate at higher altitudes) or geniculate and rooting at lower nodes (facultatively stoloniferous), branched or unbranched; nodes glabrous; internodes (0.5–)3–26 cm long, soft or sometimes slightly lignified, hollow. Leaf sheaths longer or shorter than the internodes, round or flattened, glabrous on sides and margins; ligules (1.5–)5–12(–15) mm long, hyaline to membranous, apically attenuate but often becoming lacerated due to mechanical damage; blades (3–)5–50 ×0.2–0.6 cm, flat but becoming inrolled when dry, glabrous to somewhat scabrous above and below. Panicles (1.5–)15–105 ×2–30 cm, partially inserted below (subspp. *muelleri* and *fascicularis* or occasionally subsp. fusca) to completely exserted at maturity; with (3–)5–35 branches; the branches (1.5–)3–20 cm long, alternate along the rachis, sometimes reflexed or steeply erect but mostly somewhat ascending, stiff, minutely scabrous, axils glabrous. Spikelets 5–12(–14) mm long, shortly pedicillate, sometimes distant near base of branches but overlapping near branch tips; florets (4–)6–12(–20); callus glabrous or hairy; lower glumes 1.0–3.5 (–4.9) mm long, membranous, narrowly ovate to ovate, usually scabrous on the midnerve, apex broadly acute to acute, awnless or infrequently shortly awned; upper glumes 1.8–5.5 mm long, elliptic to usually ovate or widely ovate (or sometimes obovate), scabrous on midnerve, apex obtuse (rarely) or acute at apex, rarely short-awned; lemmas 2.3–6.0 mm long, narrowly ovate, ovate, or elliptic, the lateral nerves distinct and sometimes slightly excurrent, more or less sericeous on lateral nerves and the midnerve (hair tips rounded), apex truncate, obtuse, to acute or acuminate and sometimes bifid, awnless, mucronate, or awned to 3.5 mm; palea subequal or slightly exceeding lemma, more or less sericeous on nerves; apex acute or obtuse. Stamens 1, 2 or mostly 3; anthers 0.2–2.7 mm. Caryopses 1.0–2.4 ×0.7–1.2 mm, elliptic, ovate, or obovate in hilar profile, transversely elliptic to depressed obovate in transverse section, hilar groove lacking, smooth or sometimes slightly rugose, brown; pericarp weakly adnate to the endopserm.

##### Stem anatomy.

Stems are hollow in *Diplachne fusca* (Canfield 1934; Ebinger and Carlen 1975; Brown 1975; Auquier and Sommers 1967; Valls 1978). When branching occurs in *D. fusca* it tends to be concentrated in the upper nodes (Valls 1978). Valls (1978: 33) observed that branching in D. f. subsp. uninervia tends to occur after the terminal inflorescence is fully developed, suggesting that branching is facultative and dependent on favourable growing conditions, presumably adequate soil moisture.

##### Phenology.

Flowering throughout the year in tropical latitudes; usually commencing early to mid-summer in temperate areas.

##### Distribtution.

**Native**: Widespread and common to abundant in warm–temperate and tropical areas, between approximately 49°N and 40°S in the New World and 40°N and 42°S in Old World; mostly below 2000 m. **Non-native**: See under subspecies.

##### Vernacular names.

Malabar sprangletop; Chinese: shuang fu cao (双稃草) (and see others under subspecies).

##### Comments.

Localised populations of the *Diplachne fusca* complex can be somewhat distinct morphologically from conspecifics occurring elsewhere, which is reflected in the many names that have been created to reflect such variation. However, all characters intergrade when considered globally (Snow 1997a), suggesting that the localised morphological variants do not merit recognition at the specific level. Field observations, herbarium work and multivariate statistical studies (Snow unpubl.) based on eleven population samples (n=20) from North America, Africa and Australia, which included over 80 morphometric traits, supported the recognition of four subspecies, which generally can be differentiated with little difficulty. These include: D. fusca subsp. fusca, a polymorphic Paleotropical taxon adventive in a few areas in the New World (Nicora 1995; Snow 2012); D. f. subsp. muelleri, known from much of the interior portions of Australia, particularly the Northern Territory; *D. f. subsp. uninervia*, native to the Neotropics but adventive elsewhere (e.g. Snow and Simon 1999) and *D. f. subsp. fascicularis*, native to the temperate and tropical regions of the New World.

Differentiating between subspecies can be particularly difficult in parts of California and Argentina, where *D. f. subsp. fusca* is adventive and sympatric with subspecies fascicularis and *uninervia* and in the Middle East and Australia (Western Australia, Queensland), where D. f. subsp. uninervia has become established (Snow and Simon 1999). It seems unlikely that D. f. subsp. fusca has persisted in California.

Specimens collected around wool combing mills in South Carolina in the United States by Ahles and associates in the 1950s and 1960s appear to be L. fusca subsp. fusca or occasionally subsp. muelleri, which likely arrived from wool exported by Australia to the Carolinas for the textile factories then common.

##### Key to subspecies of *Diplachne fusca*

**Table:** 

1	Lowermost panicle branches generally exserted from sheath; uppermost leaf blade length generally shorter than the terminal panicle; leaf sheaths only rarely mottled with anthocyanin pigments; lemmas at maturity smoky white or not, sometimes dark green, but usually lacking a distinct dark spot	2
–	Lowermost branches of panicles generally partially to mostly inserted in upper sheath; uppermost leaf blade length usually exceeding length of panicle; leaf sheaths sometimes mottled with anthocyanin pigments; lemmas often smoky white at maturity and with a dark spot in lower half	3
2	Lemma apices various, obtuse to acute or acuminate, notched or not; lemmas of various colours; spikelets 6–14 mm; anthers usually 0.5–2.7 mm; mostly Old World, southern South America, introduced into North America	**Diplachne fusca subsp. fusca**
–	Lemma apices obtuse to truncate, usually notched and often mucronate; lemmas often dark green or lead coloured; spikelets relatively short, 5–10 mm, anthers usually less than 0.7 mm; rachilla rarely visible during anthesis; mostly New World tropics	**Diplachne fusca subsp. uninervia**
3	Lemmas flat, relatively broad, to 2.0 mm wide; panicles narrow, mostly less than 5 cm wide; panicle branches generally steeply erect, often flexuous near tips; hairs on lateral nerves of lemma sericeous to velutinous, often densely so and typically becoming divaricate with age; lemma apices mostly broadly acute, awnless or sometimes mucronate; Australian interior	**Diplachne fusca subsp. muelleri**
–	Lemmas slightly keeled, relatively narrow, mostly less than 1.5 mm wide; panicles somewhat broad, particularly at base, to 22 cm wide; panicle branches somewhat erect to reflexed, the branches not flexuous near tips; hairs on lateral nerves of sericeous, rarely densely so, typically remaining more or less appressed; lemma apices acute to acuminate, awnless or with awns to 3.5 mm long; mostly New World	**Diplachne fusca subsp. fascicularis**

#### Diplachne fusca subsp. fusca

Taxon classificationPlantaePoalesPoaceae

(L.) P. Beauv. ex Roem. & Schult.

Figure 2

Poa malabarica L., Sp. Pl. 69. 1753, nom. utiq. rej. (Snow and Davidse 1998). Panicum malabaricum (L.) Merr., Philipp. J. Sci. 4: 248. 1909. Ottochloa malabarica (L.) Dandy, J. Bot. 69 (2): 55. 1931. Diplachne malabarica (L.) Merrill, Bull. Torrey Bot. Club 60: 635. Leptochloa malabarica (L.) Veldkamp, Blumea 19: 64. 1971. 1933. **Type.** Rheede, Hort. Malab. 83, t. 45. 1703! (lectotype: designated by Merrill, Bull. Torrey Bot. Club 60: 635. 1933).
Festuca reptatrix L. Sp. Pl. (ed. 2). 1: 106. 1762. Diplachne reptatrix (L.) Druce, Bot. Arr. Brit. Pl. (ed. 2), 129. 1928. **Type.** Linn. Herbarium (lectotype: LINN 92.20! [left–hand specimen], designated by Snow 2000).
Bromus polystachios Forssk. Fl. Aegypt.–Arab. 23. 1775. **Type.** EGYPT. Alexandria, P Forsskål 1016 (holotype: C [C10001861]!).
Poa contracta Retz. Observ. Bot. 3: 11. 1783. Leptochloa contracta (Retz.) Blatt. and McCann, Sci. Monogr. Imp. Counc. Agric. Res. 5: 243. 1935. **Type.** INDIA. JG König s.n. (fragment, K!).
Festuca indica Retz., Observ. Bot. (Retzius) iv. 21. 1786. Diplachne indica (Retz.) Spreng., Syst. Veg. 1: 351. 1825. Tridens indicus (Retz.) Nees in Wight, Cat. Ind. Pl., 106. 1794, nom. nud. **Type.** INDIA. JG König s.n. (holotype: LD [1287467!]; fragment, K!; isotype: BM!). Contrary to Snow (1997a: 225), lectotypification is not needed now that the location of the holotype is known.
Triodia parviflora R. Br., Prodr. 182. 1810. Festuca brownii F. Muell., Fragm. 8: 129. 1873. Diplachne parviflora (R. Br.) Benth., Fl. Austral. 7: 620. 1878. Leptochloa parviflora (R. Br.) Verloove & Lambinon, Syst. Geogr. Pl. 76: 219. 2006. **Type.** AUSTRALIA. Northern Territory, Arnhem Bay, “Littora Nova Hollandiae intra tropicum”, R. Brown 6254 (lectotype, here designated: BM! [BM000514993]; isolectotype: K [K000899898]!; isolectotype fragment: BRI!).
Triodia ambigua R. Br., Prodr. 183. 1810. **Type.** AUSTRALIA. Queensland, Keppel Bay, R Brown 6253 (lectotype, here designated, BM! [BM000514991]; isolectotype: BM!; BM fragment, BRI!).
Tridens capensis Nees, Linneana 7: 324. 1832. Uralepis capensis (Nees) Kunth, Enum. Pl. 1: 319. 1833. Diplachne capensis (Nees) Nees, Fl. Afr. Austr. 256. 1841. Triodia capensis (Nees) Th. Dur. and Schinz, Consp. Fl. Afr. 5: 877. 1895. **Type.** SOUTH AFRICA. “Bei Doornhoogte in der Capschen Fläche, Dec. 1824, Ecklon”. No specimen is cited in the protologue; lectotypification is probably needed.
Diplachne capensis (Nees) Nees var. concinna Nees, Fl. Afr. Austr. 257. 1841. **Type.** SOUTH AFRICA. Ad Weltevrede in ripa Gamka..., Drège s.n. (lectotype, here designated: B ([! by Snow {1997a, but unpublished}; not found electronically online]; isolectyotypes: BM! P [P00083372], [P00083373]!; fragment: PRE!). Note: Drège or a later worker assigned this species or sheet the number 2553, which at PRE is also assigned to Diplachne livida (below).
Diplachne capensis (Nees) Nees var. minor Nees, Fl. Afr. Austr. 257. 1841. **Type.** SOUTH AFRICA, Drège 3900 (fragment of holotype: PRE!).
Diplachne capensis (Nees) Nees var. obscura Nees, Fl. Afr. Austr. 256. 1841. **Type.** SOUTH AFRICA. Circa Graaf Reynet solo Karro, Ecklon s.n.; In Herb. Reg. Berol, Mundt s.n.; Ad sinum Plettenbergbai, George s.n., (fragments syntypes [Ecklon s.n., Mundt s.n.]: PRE!)
Diplachne capensis (Nees) Nees var. prolifera Nees, Fl. Afr. Austr. 257. 1841. **Type.** South Africa, Ad flumen Gauritzrivier solo Karro, George s.n. (Fragment of type: PRE!)
Diplachne capensis (Nees) Nees var. prolifera subvar. minor Nees, Fl. Afr. Austr. 257. 1841. **Type.** South Africa. Inter Los–Tafelberg et Zwartkey, JF Drège 3900 (lectotype, here designated: P [P00083374]!)
Diplachne capensis (Nees) Nees var. pauciflora Nees, Fl. Afr. Austr. 257. 1841. **Type.** South Africa. In districtus Caledon montibus. Since Poaceae types of Nees were destroyed at B, selection of a neotype may be necessary.
Diplachne livida Nees, Fl. Afr. Austr. 254. 1841. Uralepis livida (Nees) Steud., Syn. Pl. Glum. 1: 248. 1855. Triodia livida (Nees) Th. Dur. and Schinz, Consp. Fl. Afr. 5: 877. 1895. **Type.** SOUTH AFRICA. Namaqualand, In valle praerupta montium Kamiesbergen prope Kuile alt. 3000’... JF Drège (2553?; see comment above under Diplachne capensis var. concinna) (holotype: B!; isotypes: BM! P [P00439436], [P00439437], [P00083371]!).
Diplachne pallida Hack., Bull. Herb. Boiss. 3: 387. 1895. **Type.** SOUTH AFRICA. Transvaal, Boshveld, Klippan, A Rehmann 5371 (holotype: W-17607!; isotype: K [K000366665]!, fragments: PRE!, US! [US-865866]).
Leptochloa neuroglossa Eichinger, Repert. Spec. Nov. Regni Veg. Beih. 40 (1, Anhang): 75. 1930, and 264, 1931. **Type.** TANZANIA. Pare District, Mkomazi, Lake Manga, A Peter 41078 (holotype: B†). A neotype specimen at B (!) (“S. Pare, km 164 Sumpfgebiet Mkomasi -Mkumbara, A Peter 10497”) was suggested earlier (Snow 1997a: 227) but the specimen has not been seen recently.
Diplachne cuspidata Launert, Prod. Fl. Südwestafrika 34 (160): 221. 1970. **Type.** NAMIBIA. Etoscha-Pfanne, Okaukuejo, 5 Meilen westlich von Okondeka im Omuramba, 27 Feb 1963, W Giess, OH Volk, & B Bleissner 6105 (holotype: M [M0103386]!; isotype: BM [BM000514989]!).
Diplachne amboensis Roiv., Ann. Bot. Fenn. 11: 34. 1974. **Type.** NAMIBIA. Ovamboland, Ondonga, M. Rautanen s.n. (holotype: H! [H1057541]; isotype: H [H1003258]!; Z [Z-000017953]!).
Diplachne amboensis Roiv. var. plurinodis Roiv., Ann. Bot. Fenn. 11: 36. 1974. **Type.** NAMIBIA. Ovamboland, Ongandjera, Okahao, pool in valley, Soini s.n. (holotype: H! ([H1057542]).
Diplachne wahlbergii Roiv., Ann. Bot. Fenn. 11: 36. 1974. **Type.** SOUTH AFRICA, Caput Bonae Spei, J Wahlberg s.n. (holotype: S!; isotype: K!, S!).

##### Type.

PALESTINE. Ab. loco, *F. Hasselquist s.n.* (lectotype: LINN 92.21!), typ. cons. prop.

##### Description.

Plants annual or (mostly) perennial. Culms (15–)40–170 cm tall, 2–5 mm wide at base, round or somewhat flattened, ascending to erect or sometimes decumbent and rooting at nodes (and sometimes strongly stoloniferous in southern Africa), often branching; nodes glabrous; internodes to 26 cm long, soft or sometimes lignified, hollow. Leaf sheaths longer or shorter than the internodes, round or somewhat flattened, glabrous on sides and margins; ligules 4–8(–15) mm long; blades mostly 5–48 cm long, 2–6 mm wide, glabrous to moderately scabrous above and below. Panicles 15–105 cm long, 2–20 cm wide, exerted or occasionally partially inserted at maturity with 3–28 branches; the branches (1.5–) 4–20 cm long, mostly alternate along the rachis, ascending, erect, or infrequently reflexed, stiff to somewhat flexuous, minutely scabrous, axils glabrous. Spikelets 6–14 mm long, distant to tightly imbricate; florets (4–)6–12(–14); callus glabrous or hairy; lower glumes 1.9–3.0(–4.9) mm long, ovate, scabrous on midnerve, obtuse to acute, rarely bifid; upper glumes 3.0–4.7(–5.5) mm long, ovate, scabrous on midnerve, obtuse to acute, rarely bifid; lemmas 3.0–4.7(–6.0) mm long, 3-nerved, narrowly ovate to ovate, the lateral nerves pronounced and often extending to the tips, sericeous (at least below) on lateral nerves and often on midnerve, glabrous between nerves, apex obtuse to acute or acuminate, sometimes bifid, unawned, mucronate, or awned; paleas subequal to slightly exceeding lemma, elliptic, more or less sericeous along nerves; apex obtuse to acute. Stamens 1–3; anthers 0.5–2.7 mm long (typically longer in perennials), maroon or yellow. Caryopses 1.6–2.3 mm long, 0.9–1.2 mm wide, obovate in hilar profile, transversely elliptic to depressed obovate in transverse section, hilar groove lacking, smooth, brown; pericarp weakly adnate to endosperm.

##### Leaf anatomy.

Midrib present (or rarely absent) in mature leaves; central lacuna present. **Primary bundles**: protruding adaxially and abaxially and to a greater degree than secondary bundles; outer bundle sheaths continuous adaxially, interrupted abaxially; extension cells present adaxially; adaxial cells enlarged, abaxially cells not enlarged; sclerenchymatous girders present adaxially and abaxially, or abaxially only as strands. Colourless cells present between primary and secondary bundles; chlorenchyma continuous or discontinuous between adjacent bundles. **Secondary bundles**: protruding adaxially or not, flush abaxially; outer bundle sheath continuous or interrupted abaxially; sclrenchymatous girders present abaxially. (Vouchers [all at MO] and country of orgin: Snow & Chatakutah 6767, 6829, 6908 (Botswana); Snow & Burgoyne 7108 (South Africa); Snow & Burgoyne 7176, 7196 (Namibia); Snow et al. 7208, 7214, 7215, 7217 (Australia); Ellis specimens from South Africa [all at PRE]: Ellis 1908, 3410, 3653, 4779.)

##### Stem anatomy.

Stems can root at the nodes and functionally act as stolons (e.g. Hitchock 24410 [US], Drège 3900 [P]; Valls 1978). When branching occurs, it is mostly towards the base and at lower nodes. Elliptic air canals subjacent to the epidermis of culms typically are present in populations emergent in water.

Culms hollow; pith parenchyma isodiametric; inner sclerenchymatous ring present; peripheral sclerenchymatous ring present; inner sclerenchymatous ring canal tissue present (see Culm Anatomy under *D. gigantea*); Kranz sheath cells absent; Kranz sheath cell canal tissue absent. Vouchers (at MO): Snow & Chatakutah 6808 (Botswana); Snow & Chatakutah 6829 (Botswana); Snow & Burgoyne 7196 (Namibia); Snow et al. 7217 (Australia); Snow et al. 7208 (Australia); Snow et al. 7239 (Australia); Snow et al. 7249 (Australia).

##### Chromosome numbers.

*n*=10 (Gould 1958, 1966, 1968; Reeder 1971; Valls 1978; Spies et al. 1991); 2*n*=19 (Spies and Vogues 1988); 2*n*=20 (Larsen 1963; Bir and Sahni 1986).

##### Phenology.

Flowering throughout the year but seasonally locally.

##### Distribtution.

**Native**: Much of paleotemperate and paleotropical areas; mostly open mesic areas, often in saline conditions. Elevation sea level to ca. 2000 m. (TDWG: ANG, BOT, BZE-PB, BUR, CHA, ETH, JAW, IND, IRQ, ITA-IT, KEN, MDG, MLW, MLY-SI, MOZ, MYA, NAM, NGA, NGR, NSW-NS, NTA, OFS, PAK, PHI, QLD-QU, SEN, SOA, SRL, TAI, TAN, THA, UGA, VIC, WAU-WA, YEM-NY, ZAI.) **Non-native**: Persisting in Argentina, but non–persisting elsewhere. (TDWG: AGE-BA, AGE-ER, AGE-MI, AGS-RN, AGW-LR, AGW-SJ, BOL, CAL, CZE-CZ, GBR, GER, NWG-PN, SCA, SWI.). Diplachne fusca subsp. fusca was grown in experimental gardens in the early part of the 20^th^ century in Chico and Berkeley, California, as introductions from New South Wales (McKee, SPI 17213 [NA]; Wight 1359 [NA] (Beetle s.n., 1941 [NA]). It has been collected outside the plots in California, but evidently has not become widely established in that state.

##### Conservation status.

Widespread; not of concern.

##### Etymology.

The Latin *fuscus* refers to dark, dusky, or swarthy and possibly alludes to the dark green or plumbeous spikelets of some specimens.

##### Vernacular names.

Malabar sprangletop; Beetlegrass sprangletop. Japan: Hama-gaya (Koyama 1987). Tanzania: Msinti (Fipa and Kifipa); Milepa (Bullock 3605 and 2474, US); Hîsh (Simpson 5183, B). Iraq: Sabut (Springfield 12545, US). Egypt: Sayfoum, (Drar 9/123, NY). China: Shuang fu cao. Myanmar: Myet–cho.

##### Comments.

Diplachne fusca subsp. fusca is the most widely distributed taxon in the complex and occurs throughout the range of the species in the Paleotropics, with some introductions in the Neotropics and North America (California, South Carolina). It is sometimes considered a weed of rice paddies (McIntyre and Barrett 1985, McIntyre et al. 1989).

Distinguishing D. f. subsp. fusca from subsp. fascicularis where the two are sympatric can be difficult, particularly in Argentina (Snow 2012), where the populations of subsp. fusca may be non-native. The authors’ interpretation of taxa in the complex differs from Nicora (1995), who synonymised Diplachne uninervia (J. Presl) Parodi var. procumbens Parodi under *D. fascicularis*. In contrast, the authors synonymise the former under D. fucsa subsp. fusca. The subsp. fusca is differentiated from subsp. fascicularis primarily by the latter having panicle branches inserted at the base and having anthers shorter than 0.5 mm, whereas the former more typically has fully exserted panicles and anthers exceeding 0.5 mm (sometimes significantly so).

**Figure 2.**
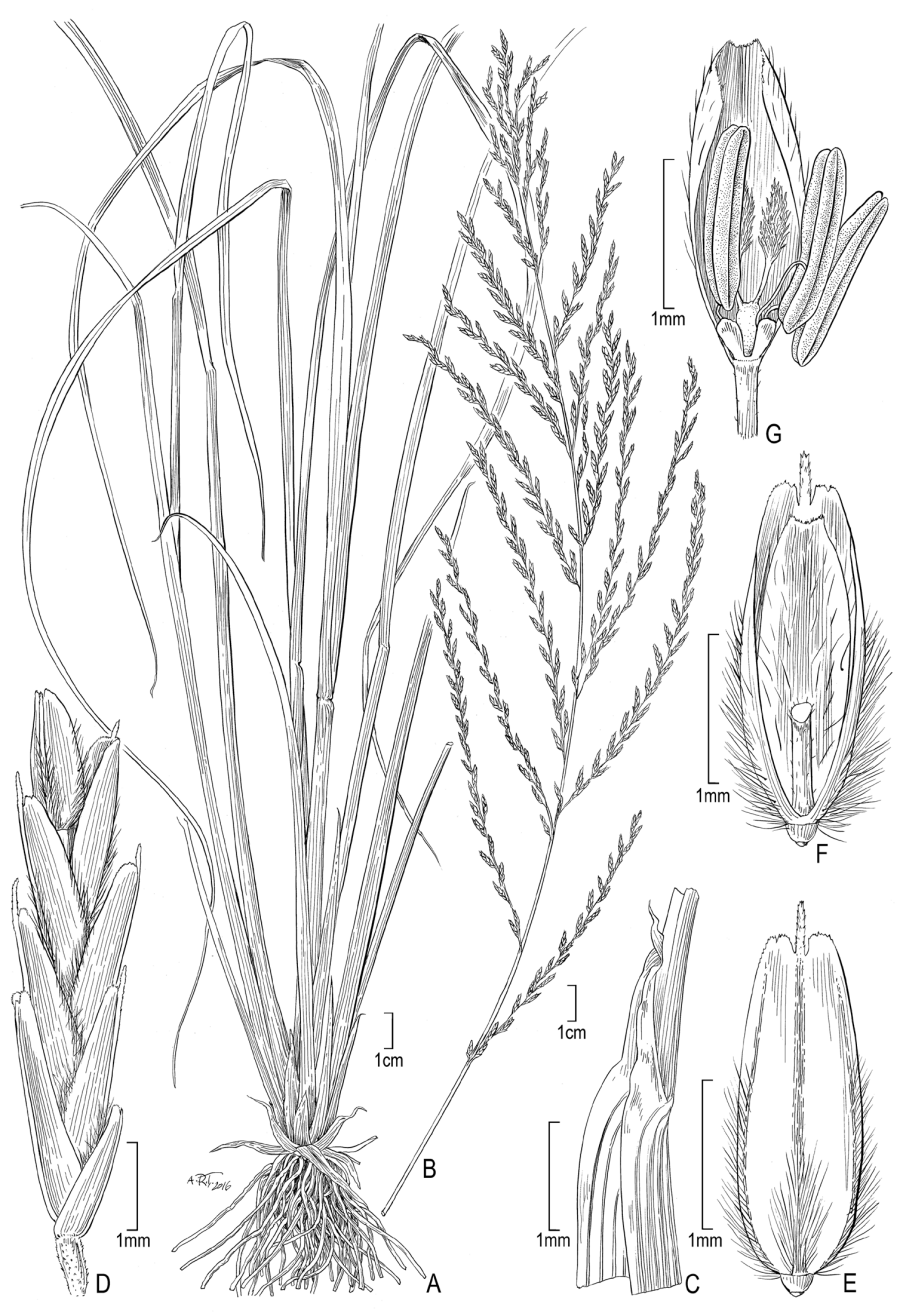
**A–G** Diplachne fusca (L.) Kunth subsp. fusca **A** habit **B** inflorescence **C** ligule **D** spikelet **E** lemma **F** floret and rachilla **G** palea, lodicules, ovary, and stamens. Drawings from *Strohbach & Sheuyange BD3162* (US).

The floret morphology of some specimens from western Africa (Audru 2704; Adam 19242; Garba 7471; Trochain 878 [all at P]), southern Africa (Snow & Chatakuta 6908 [KSP]; Strohbach & Sheuyange BD3162 [US; Fig. 2) and New Guinea [Backer 16301 {duplicates at P, U, and W}]; Backer 24130 [U]; and Brass 6065 [BRI]) closely resembles that of subsp. D. f. subsp. uninervia. The specimens from New Guinea may be from New World propagules that arrived during World War II. The spikelets of the type specimen of *Diplachne pallida* Hack. also closely resemble those of D. f. subsp. uninervia.

The length of the awns varies throughout the range of D. fusca subsp. fusca and is too inconsistent to be of taxonomic value.

The sexuality of the florets varies widely. A population from Namibia (Snow & Burgoyne 7196 [with numerous duplicates distributed]) had male, female and hermaphroditic florets within a single spikelet. The number of stamens can likewise vary within a spikelet from one to three (e.g. Snow and Burgoyne 7196), which has been noted also by Nicora (1995). The anthers also vary considerably in length between specimens, but typically are consistent within a specimen and within populations.

Thrips (Order Thysanoptera) of an indetermined genus were found in virtually every floret of fresh material of a specimen of D. fusca subsp. fusca from Queensland, Australia (Snow et al. 7249-F). In a study of germination, populations in Australia were affected more by nighttime than daytime temperatures (Myers and Morgan 1989).

Some populations of this subspecies have high to very high levels of tolerance for saline or sodic soils (Beadle 1948a-b; McVaugh 1983; Hackett and Wickens 1984; Myers and Morgan 1989; Kernick 1990; Chapman 1996; Ahmad 2009). This tendency is most developed in Australia, Asia and Africa and is least well documented for subsp. *uninervia*. For example, the label of Trapnell 1112 in Zambia states the species grows amidst ca. 5 cm of solid salts. Another specimen from Zambia (Greenway 6228) states that the species becomes a dominant pioneer in areas trampled out in brackish pans in grassland dominated by the grass genus *Hyparrhenia* E. Fourn. Others also have noted the high levels of salinity tolerance (Reinhold et al. 1986; Hurek et al. 1987; Reinhold-Hurek et al. 1993). Joshi et al. (1983) reported salt excretion on the leaves via small apical pores in papillate structures that they designated as salt glands. Chapman (1996) also reported that some populations of D. f. subsp. fusca excrete salt from glands on the leaves and that this taxon has been advocated for its use for lowering salt levels in soils. The ability of many populations of this subspecies to tolerate and even thrive in saline soils was one of many factors contributing to the decision to recognise them at the subspecific, rather than specific rank (Snow 1997a). In southern Africa, D. fusca subsp. fusca can be common to abundant in pans (low-lying areas that accumulate seasonal moisture but which dessicate in the dry season), where it sometimes is decumbent to geniculate and stoloniferous (Breen et al. 1993; e.g. see Hitchcock 24410 [US]; Snow 6829 [MO]).

Herbarium labels from African collections sometimes indicate grazing, presumably by the native fauna, and others indicate good grazing for livestock. The subspecies may occur in pure or nearly pure stands in Africa. In Australia, Lazarides (1970) reported the taxon as having high palatability and being preferentially grazed, but that it typically occurs in relatively small populations. Denny (1993a, 1993b: 27) and Ahmad (2009) indicated that among grasses generally, *Diplachne fusca* appears to maximally combine adaptations to wetness and salinity, combined with palatability, of any species of grass. Kernick (1990: 171) linked the salinity tolerance and palatability of *D. fusca* and suggested that additional screening for salinity tolerant races would be worthwhile. The salinity tolerance of D. fusca subsp. fusca, high viability of its seeds (Chapman 1996; Snow, pers. obs. in greenhouse), and high palatability of the subspecies among ruminants (Chapman 1996; Snow pers. obs.) collectively make *D. fusca* an excellent candidate for reclamation of saline soils (Haq and Khan 1971; Sandhu et al. 1981; Chapman 1996; Ahmad 2009).

A remarkable biological attribute of Diplachne fusca subsp. fusca is the presence in some Pakistani populations (where it is called Kallar grass) of a recently described nitrogen-fixing genus of bacteria, *Azoarcus* (Reinhold et al. 1986, 1993; Hurek et al. 1987, 1993, 1994; Reinhold-Hurek and Hurek et al. 1997). Subsequent studies documented *Azoarcus* to be a nitrogen-fixing endosymbiont of many grasses, including rice (Hurek et al. 1997; Krause et al. 2006). Given increasing problems of salinisation in some areas and the biologically and economically important properties of the Pakistani populations (nitrogen-fixing, salinity tolerance, perennial growth form and high palatability), these populations should be investigated further for their reclamation potential of saline soils, given that they also could provide useful fodder. More recent research has examined the rhizosphere for bacterial species of *D. f.* growing in industrial sites (Abou-Shanab et al. 2005).

*Diplachne fusca* has been studied recently augmented with three experimentally introduced endophytic bacteria, as a species for use in constructed wetlands for bioremediation of wastewater from tanneries (Ashraf et al. 2017).

##### Specimens examined.

**Afghanistan**. Maymana: 25 km SW of Maymana, Alizzi & Omar 34707 (K). **Angola**. Huila. Morros de Cualeque, Exell and Mendonça 2704 (BM); Distrito de Huila, Pereira de Iça, Cunhama, Gossweiler 11066 (K, US). **Argentina**. Buenos Aires: Pdo. Magdalena, Punta Indio, Boelcke et al. 12540 (MO); A San Vincente, Zardini 246 (LP); Vicente Lopez, Parodi 7694–1/2 (BAA); Vicente Lopez, Parodi 184 (BAA); Avellaneda, prox. al cemetario, Parodi 4774 (BAA); San Isidro, Parodi 4903 (BAA); San Vicente, Parodi 8118 (BAA, US); Palermo, Parodi 175 (BAA). Entre Rios: Dpto. Uruguay, Laguna de los Negros, Nicora 3012 (SI); Dpto. Uruguay, cerca del Parque Unzué, Burkart 25675 (BAA). Misiones: Nacanguazú, Santo Pipó, Grondona and Puccinini 3188 (UC). La Rioja: Cunta de Llanos, Nicora 18527 (B). Rio Negro: Fuerte Gral Roca, Boelcke & Serrano 3122 (BAA). San Juan: Jachal, Cabrera 18025 (LP). **Australia**. New South Wales: 24 km S of Moree, Beadle s.n. (NE 24646), Combanning Silo, ca. 13 km E of Temora on Stockinbingal Rd., ca. 26 km W of Stockinbingal, Snow et al. 7208 (KSP, MO, NSW); Ca. 20 km E of Griffith on Ardelthan Rd., Snow et al 7213 (MO, NSW); 9.7 km S of Darlington Point on Coleambally Rd., S of Griffith, Snow et al. 7214 (KSP, MO, MU, NSW); Marrimajeela Ck., ca. 15 km N of Boolingal on road to Ivanhoe, Snow et al. 7215 (MO, NSW); 15.1 km out of Brewarrina on Collerina Rd., Snow et al. 7216 (MO, NSW); 17.9 km out of Brewarrina on Collerina Rd., ca. 4.3 km E of Bokhara Riv., Snow et al. 7217 (MO, NSW); Ca. 60 km E of Brewrrina on road to Walgett, ca. 9 km W of turnoff to Cumborah, Snow et al. 7220 (MO, NSW); Southern end of town of Walgett, along muddy margins of artificial pond created by irrigation regime in the civic park, Snow et al. 7222 (MO, NSW); 46.6 km E of Connamble on road to Baradine, Snow et al. 7226 (MO, NSW); 19.9 km N of Boggabri on road to Narrabri, Snow et al. 7232 (MO, NSW); 6.5 km N of Edgeroi, between Narrabai and Moree, Snow et al. 7234 (MO, NSW); 11.6 km N of Moree on road to Boggabilla (Hwy 39), Snow et al. 7237 (MO, NSW); Ca. 58 km N of Moree on road to Boggabilla (Hwy 39), Snow et al. 7244 (MO, NSW); Grenfell, Corbett s.n. (BISH); Duck Ck., Wollongbar, McBarron 6516 (NSW) and McBarron 6516 (NSW); Nevertire, Helms 31318 (NA, NY, SMU, US); Walgett, Vickery 18009 (US); Macquarie Marshes area, 16 km SE of Carinda, Paijmans 1737 (CANB); Bogan Gate – 4 km E of Parker Rd., Lloyd 156 (CANB); Moree – 3 km E on Gwyndir Hwy, Lloyd 726 (CANB); 28 mi. S of Nyngan, along road between Nyngan to Narromine, Martensz 268 (CANB); Macquarie Marshes, Warren, Goodrick 261 (CANB); Agric. Res. Station, Yanco, Boerema 7 (CANB); Riverina Dist., Booroorban, between Deniliquin and Hay, Brown 390 (BRI, CANB); Near Barham, Ganba 3516 (CANB); Griffith, 1.7 km from W end of Hamilton Rd., Lodder and Corbett 11 (CANB); Near Homebush, Sydney, Hubbard 8185 (BRI); Fort Bourke, Pear Bore No. 1, Simon 2957 (BRI); 14 km SE of Bourke on Bourke–Nyngan Rd., Simon 2974 (BRI); Near Goonery Bore, 65 mi. E of Wanaaring, Moore 5781 (BRI); Between Fort Grey and Cameron’s Corner, Corrick 6536 (BRI, MEL); Macquarie Marhes 35 km W of Quambone, just E of Macquarie Riv., Paijmans 1704 (CANB); West of Premer, Hosking and Sullivan 472 (MEL, NE); Gulpa, Brown 389 (MEL); “Mt. Mulgah” – ca. 60 km NW of South, Moore 8231 (CANB); “Mt. Mulgah” – ca. 50 mi. NW of South, Moore 5091 (CANB); Ca. 51 mi. W of Bourke on Wanaaring Rd., Moore 5112 (CANB); 12 km W of Booligal, Paijmans 1649 (CANB). Northern Territory: MacDonnell Range, Palm Valley Canyon, Finke Riv. Canyon, SW of Alice Springs, Vasek 680915–25 (CANB, RSA); 40 km W of Suplejack Homestead, Latz 8121 (BRI); Gardens Station, Latz 1939 (BRI, CANB); Warangaiyu Lagoon, Elcho Island, Latz 6270 (CANB); Coomarie Spring, Latz 3956 (CANB); 7 km SW Borroloola, Latz 1523 (BRI, CANB); Homestead Ck. Bing Bong Station, Latz 1493 (CANB); Gulf of Carpenteria, Maria Island, Dunlop 2800 (BRI, CANB); Swamp below Howard Springs, Darwin area, Craven 4474 (CANB); Coastal Plains Research Station, 30 mi. SE of Darwin, Lazarides 6810 (CANB); South Alligator Floodplain, 16 km S Arnhem Hwy, Wightman 1456 (BRI, CANB); Glen of Palms, James Range, Gardner 11728 (MEL); Glen of Palms, ca. 112 km SW of Alice Springs, Beauglehole 23846 (BRI, MEL); 11 km from Borroloola, Daly Waters Rd., Jacobs 1670 (CANB, NSW); Fogg Ck. Dam near Beatrice, Jacobs 1767 (CANB); Palm Valley, 10 mi. S of Hermannsburg Mission, Perry 3501 (BRI, CANB); Mangrove area on banks of South Alligator Riv. Paijmans LAC4486 (CANB); Palm Valley, 12 mi. SW of Hermannsburg Mission, Lazardies 5290 (BRI, US); Finke Riv. 2 mi. S of Hermannsburg, Latz 3125 (BRI); Darwin, Leanyer Swamp, Latz 3616 (BRI); Fish Ck., Woolner Station, Rankin 2491 (BRI, CANB); Wirra Lagoon near Nhulumbry, Scarlett Y–257–74 (BRI); Palm Valley, Beauglehole 10359 (BRI); Palm Valley, Beauglehole 27518 (BRI); Mary Riv. Conservation Reserve, Liddle 1102 (BRI). Queensland: Burke Dist., 12 mi. E of Hughenden Township, Lazardies 3605 (BRI, MO, US); Between Mt. Emu Plains and Mt. Sturgeon Stations, Hubbard & Winders 7560 (BRI); Normanton, Blake 9002 (BRI); Agnes Lake, 79 km S of Lyndhurst Station on Hann Hwy, Simon and Clarkson 2752 (BRI); Mount Isa, Winders 7427 (BRI); Nonda, Hubbard & Winders 7228 (B, BRI, CANB, K, MO, NSW); Mt. Isa, No. 7 Tailings Dam, Schmid 712 (BRI); 3 km NE of Burketwon on Truganini Crossing Rd., Jacobs 1318 (CANB, NSW); Dry bed of Lake Louisa ca. 90 mi. N of Hugenden, Blake 8605 (CANB); 19 mi. S of Mt. Isa Township, Lazarides 4381 (US); 18 mi. SW of Claraville Station, Lazarides 3934 (CANB); 8 mi. E of Iffley Station, Lazarides 3939 (CANB); 8 mi. N of Cloncurry Township, Lazarides 4315 (CANB); 3 mi. SW of Malbon Township, Lazarides 4400 (CANB, MO). Cook Dist., 1.6 km S of mouth of North Kennedy Riv. on Lakefield N.P., Neldner and Clarkson 3782 (BRI, NSW); Arriga area, ca. 13 km SW of Marreba, T. Mailsel’s Farm, Pregno s.n. (BRI); 2.5 km SW of the aboriginal settlement at Mapoon, Clarkson 4962 (BRI, K); Weipa, Morton 1132 (MEL); 10 mi.NNE of Lyndhurst Station, Lazarides 3737 (CANB); Station Ck. saltpan, 1 km E of Inkerman homestead, Neldner & Clarkson 2873 (BRI, NSW); On southern bank of Nassau Riv., 0.5 km upstream from junction with Rocky Ck., Neldner & Clarkson 3007 (BRI); 9 km NW of Kenchering by road, Dalliston CC437 (BRI); Chohan’s Farm, Ariga W of Mareeba, Hawton s.n. (BRI); Lakefield N.P., Knifehole, 2 km from Saltwater Ck. crossing on the Musgrave to Lakefield Rd., Clarkson & Simon 7061 (BRI); Plains on eastern bank of Nomenade Ck., Morton AM1132 (BRI); 2 km N of the Morehead Riv. Crossing on the Lakfield to Musgrave Rd., Clarkson & Simon 7047 (BRI); 3 mi. ESE of Spring Ck. Station, Lazarides 3807 (BRI). Darling Downs Dist., ca. 10 mi. SW of The Gums on roadside, Johnson 729 (BRI); Kindon Station, 54 mi. NNW Goondiwindi, Smith 549 (GH); 28 km E of Goondiwindi on road to Yelarbon, Inglewood and Warick, Snow et al. 7249 (BRI, KSP, MO, NSW); 3.2 km N of junction with Cunningham Hwy (Hwy 42) on the Leichhardt Hwy, N of Goondiwindi, Snow & Simon 7312 (BRI, MO); 14.5 km N of Cunningham Hwy along the Leichhardt Hwy N of Goondiwindi, 3.1 km S of turnoff to Toowoomba (78 km S of Moonie), Snow & Simon 7326 (BRI, MO); 17.8 km W of Moonie on Moonie Hwy, Snow & Simon 7360 (BRI, MO); 22 km W of Moonie along Moonie Hwy, Snow & Simon 7363 (BRI, MO); Palardo, W of Miles, Blake 7619 (BRI, CANB, NSW); ca. 10 mi. SW of The Gums, Johnson 729 (CANB, NY); Yelarbon, Blake 10445 (BRI); Yelarbon 41 km E of Goondiwindi, McDonald 406 (BRI, CANB, K); Chinchilla, Hubbard and Winders 6405 (B, BRI, CANB, MO); ca. 7 mi. SE of Meandarra, Johnson 1206 (B, BRI); Condamine State School, Turnbull 5 (BRI); Milmerran, Hubbard 5867 (BRI, CANB); Dalby, Beiers 43 (BRI); Chinchilla, Beasley 124 (BRI); Dalby, Beiers 63 (BRI); 10 Meilen von Meandarra, Walter and Walter 2610 (B); near Gurulmundi, Hubbard 5129 (B, CANB, MO); Yelarbon, Everist 889 (BRI); Pelican Lakes 30 km NE of Chinchilla, Simon et al. 3491 (BRI). Gregory North Dist., Boundary Gully Bore, 25 km N of Headingly homestead, Neldner & Stanley 1804 (BRI). Gregory South Dist., 0.7 km SW of Longford turn–off, Thompson Devel. Rd., Prendergast HDP313 (BRI); ca. 92 km W of Betoota, Nicolson & Novelly 277 (BRI). Leichhardt Dist., Broadsound Shire, “Iffley”, 3 km N of Mt. Coxendean, Anderson and Russell 896 (BRI); Broadsound Shire, Anderson 752 (BRI); Emerald, Finlay & Farquhar 2 (BRI); Broadsound Shire: “Barwon Park” ca. 70 km N of Blackwater, Anderson 2054 (BRI) and (ibid.) Anderson 2055 (BRI); Gunnewin via Roma, Kieseker 9 (BRI). Maranoa Dist., Noondoo near Dirranbandi, Blake 10690 (BRI); 13 km E of Mervyndale, Walrus III, Site R030, Purdie 462D (BRI); 40 mi. W of St. George, Roe s.n. (CANB); “Warrie”, Nindigully, Allen 111 (CANB); Noondoo Station E of Dirrabandi, Everist 817 (BRI); Mitchell, Hubbard and Winders 6309 (BRI); Boatman Station, Everist 2857 (BRI, K, NY); 15 km S of Roma, Bungil Shire, Silcock S721 (BRI); Roma, Blake 10894 (BRI); Bollon, along damp creek bands, White 11545 (GH, US). Mitchell Dist., Yaraka, Bownman s.n. (BRI); 2 mi. W of Blackall, Everist 2089 (BRI); 34 mi.NW of Longreach, Davidson 82 (BRI); 11 km SE of Corinda homestead, Thompson and Sharpe MUT1 (BRI); Jericho, Blake 10245 (BRI). Moreton Dist., Wyampa, near Bald Hills, Blake 213 (BRI); Near Redcliffe, Hubbard 5551 (B, BRI, CANB, NSW); Currumbin, White 8728 (BRI, NY); Nundah, Brisbane, Blake 159 (BRI); 2 km N of Coolum beach, ca. 130 km N of Brisbane, Sharpe 2127 (BRI); Serpentine Ck. and environs, ca. 11 km NE of Brisbane, Durrington 435 (BRI); 2 km N of Coolum Beach, ca. 130 km N of Brisbane, Sharpe 1894 (BRI); Hope Island, 2 km from Boyankil on road to Pacific Hwy, Simon et al. 2932 (BRI). North Kennedy Dist., Toonpan – Antil Plains, Everist 9194 (BRI); Mt. St. John, Hubbard & Winders 6943 (BRI, CANB); Barratta Ck., lower Burdekin Valley, Paijmans 3508 (CANB); Near Saltern Lagoon, 5 km W of Valley of Lagoona HS, Lazarides 8167 (BRI, CANB); Cromarty, between Townsville and Ayr, Blake 8306 (BRI). Port Curtis Dist., Gladstone, Blake 12784 (BRI); Curtis Island, N of Southend, Blake 22578 (BRI, CANB, MO); Curtis Island, S end, Blake 22522 (BRI); 25 km ESE of Rockhampton, Paijmans 2016 (CANB); E side of Herbert Ck., 5 km NW of “Banksia” homestead, Paijmans 2017 (CANB); Gladstone, Blake 12784 (CANB); “Torilla” between Broad Sound and Shoalwater Bay, Blake & Webb 15615 (BRI, NSW). South Kennedy Dist., 17 mi. NE of “Mirtna” Station, Adams 1133 (CANB); `Cassiopeia’, the property of J. Mendazona, Belyando Shire, Jacobsen E 580 (BRI); 6.5 km NW of Belyando Crossing, Thompson and Sharpe BUC901 (BRI); Gormans Lagoon near Mirtna HS (site GL1), Thompson & Simon BUC755 (BRI); with 2.3 km S of Doongmabulla homestead, 197 km N of Jericho, Thompson & Henderson GAL142 (BRI); 10.5 km NE of Hyde Park homestead on road to Bulliwallah homestead, Thompson & Simon BUC813 (CANB, K); 3 km S of Doongmabulla homestead on sandy flat adjacent to Cattle Ck. [...] Thompson & Simon GAL50 (BRI); Laglan Station, ca. 105 km WNW–NW of Clermont, Smith 10293 (BRI); 3 km W of Yarromere homestead, Thompson & Henderson BUC993 (BRI). Warrego Dist., ca. 25 mi.S of Wyandra, Blake 11234 (BRI, K); Wyandra, Sutton s.n. (BRI); Curragh Station, Hubbard & Winders 6263 (K, MO, NSW); Gilruth Plains Stn, Cunnamulla, Barker 835 (CANB); “Cowley”, 40 mi. E of Quilpie, on main Quilpie–Charleville Rd., Anson 2 (BRI); Gilruth Plains, Cunnamulla, McKee 10326 (BRI, NSW); 10 km SE of Charleville along Boatman Rd., Purdie & Boyland 36 (BRI); Coongoola, between Charleville and Cunnamulla, Hubbard & Winders 6174 (BRI, CANB); “Ambathala”, 100 km WNW of Charleville on Adavale Rd., Silcock 5458 (BRI); “Peneroo” via Eulo, Young 54 (BRI); Bowalli, ca. 75 mi. SSW of Quilpie, Everist 5041 (BRI); “Wittenburra” Eulo, Ebersohn E180 (BRI); Carbeen near Cunnamulla, White 11567 (BRI, GH); Dynevor Lakes, 30 mi. E of Thargomindah, Blake 11761 (BRI); Bulloo, MacGillivray 1024 (BRI). Wide Bay Dist., Splitters Ck., Smith 437 (BRI). South Australia: 30 km W of Inllian Ck on Coober Pedy road, Badman 1282 (AD, BISH); Lower Winkie Rd.... near Berri, ca. 195 km NE of Adelaide, Kuchel 3215 (AD, BISH, BRI, MO); 32 km SW of Hawker Gate, Jacobs 3536 (BRI); Just out of Wyndham on and at base of rocky slope, Symon 5251 (CANB). Victoria: Melbourne area, between Newmarket and Showgrounds railway stations, E of Ascot Vale Rd., Flemington, Clarke 1797 (BRI, MEL); King’s Billabong, beside the Murray Riv., between Mildura and Red Cliffs, Henshall s.n. (MEL); Murray Valley area, Tatura – Irrigation Research Institute, Brown 34 (MEL); Port MacKay, Dietrich 538 (MEL); Malle Study Area, Reedy Lake Wildlife Reserve, Beauglehole 55705 (MEL). Western Australia: Wittenoom Gorge, Hammersley Range, Beauglehole 11564 (BRI); 10 km SE of Wyndham, Paijmans 2505 (CANB); 8 km SE of New Wyndham Post Office, North Kimberely, George 14546 (CANB, K, NSW); Goody Goody, Fitzgerald 65 (US); 34 mi.SW of Wyndham Township, Lazarides 3080 (NSW, US); Wyampa, near Bald Hills, Blake 213 (K); Parry Lagoon, NE Kimberley, Kenneally 10719 (CANB); Ord Riv. 25 km NE of Wyndham, Paijmans 2268 (CANB); North West Coastal Hwy, 94 km from Minilya on raod to Dampier, Simon & Stretch 3788 (BRI); 34 mi.SW of Wyndham Township, Lazarides 3080 (BRI). **Belgium**. Graviers de la Vesdre, Fasseaux 11a (K). **Bolivia**. Beni: Prov. Ballivian, Espiritu en la zona del influencia del rio Yacuma, Beck 3219 (US). Santa Cruz: Chiquitos, Est. San Ignacio, 22 km N of San Jose de Chiquitosm Killeen 1712 (MO, US); Nuflo de Chavez, San Antonio de Lomerio, Killeen 825 (MO, W). **Botswana**. Central: Mumpswe Pan, 25 mi. NNW of mouth of Nata Riv., Drummond & Seagrey 5167 (PRE); 17.6 road km N of Dibete (at turnoff to Mookane), ca. 135 Road km N of Gabarone, Snow & Chatakuta 6767 (GAB, K, MO, PRE); At km marker 16, W of Nata on Hwy to Maun, Snow & Chatakuta 6829 (GAB, K, MO, PRE); Along main Hwy between Nata and Maun, ca. 67 km W of Nata, Snow & Chatakuta 6878 (GAB, K, KSP, MO, MU, PRE); 44.3 road km from Sephophe on road towards Zanzibar, ca. 21.6 km prior to Tsetsejwe, Snow & Chatakuta 6908 (GAB, K, KSP, MO, MU, PRE); Along main hwy, 17.6 km N of Dibete (at turnoff to Mookane), ca. 135 road km N of Gaborone, Snow & Chatakuta 6913 (GAB, K, MO, PRE); Botletle delta area, NE of Mopipi, Tyers 616 (MO). Kgalagadi: Zanye Pan, NW of Hukuntsi, Ellis 2657 (K, MO, PRE). Mahalapye: Kalamare, de Beer K5 (GAB). Mohembo: Okavango Riv., Smith 2669 (K); in the sump of Nwaku Pan, Smith 2969(K). Northern: Mumpswe Pan, 25 mi. NNW of mouth of Nata Riv., Drummond & Seagriest 5167 (GAB, K); Kangwa (Xanwe), 27 km NE of Aha Hills, Wild & Drummond 6931 (BM, GAB, K); near Tschelenyane, 11 km from Lake Ngami on track to Khwebe Hills, Drummond 8747 (K). Ngamiland: Boteti Riv. lower reaches, Smith 2563 (K, PRE, US); Nxai Pan N.P., Smith 1648 (K, MO); Moremi Wildlife Reserve, Smith 811 (GAB, K, MO, PRE); Moremi Wildlife Reserve, Mexara Pan, Smith 1598 (BRI, K). **Brazil**. Paraiba: São João do Carirí, Pôsto Agro–Pequário, Mattos and Mattos 9736 (US). **Burundi**. Plaine de la Rusizi au N de Bujubura, Gihanga, Van der Veken 11182 (B, U); Plaine de la Rusizi (Prov. Bubanza), savane–palmeraie a hauteur du km 14 de la route Bujumbura–Cibitoke, Lambinon 75/42 (M, MO); Vallée Katunguru, Reekmans 3187 (MO). **Chad**. Entre Moolou et Berirem, Chevalier 10102 (P); Latir Nord Bol, Dune, Gaston 749 (P); Bahr el Ghazal–Gahine, Zolotarevsky et al. 876 (P); Bagana – Ba Gara, Murat 924 (P). **China**. Hebei: Peitaiha [=Beidaihe], Cowdry 213 (US); Tientsin, Clemens 1615 (B, BM, MO); Shen Hsien, Beach s.n. (US); Shatin, Shiu Ying Hu 12084 (A). Hong Kong: Hong Kong, Shiu Ying Hu 11785 (A, CANB). Hopei: Ch’ating, Kung 3784 (US). Heijan: Roadside ca. 10 mi. W of Hochien, Beach s.n. (US). Jiangsu: Nanking (Lotus Lake), Reeves s.n. (NY). **Democratic Republic of the Congo.** Sud-Kivu: Plaine Rusisi [=Rusizi], Germain 5576 (B, M). Distr. Liberec: garden in Raspenava, introduced with waste from wool processing, 24 Aug. 1963, V. Jehlík s.n. (PRA). **Egypt**. [Province Unknown: Lake Manzala: Masraf Abu Aziza, Mustafa & Sabet s.n. (KSC 77803)] Damietta District, Ezbet El-Burg, Mashaly s.n. (K); Aus der Umgegend von Cairo, Schweinfurth 1432 (K); Al-Faiyum District, El-Azab, Ghani 3650 (K); Faraskûr, Shabetai 33 (K); Aulad Hammam, Simpson 5183 (B); Gize, Kairo, Kneucker 865 (MU); near Cairo, Meinertzhagen, s.n. (BM); Fayoum, Drar 9/123 (NY); 166 km from Cairo on the desert road to Alexandria, Amin et al. s.n. (NY); An den Rändern der Bewässerungsgräben zwischen dem Dorfe Gîze unweit Kairo, Kneucker 254 (PR, US); Between Hamul and Baltin, Hefnawy s.n. (K); Lake-side, Manzale N of Damietta, Simpson 1451 (K); Wâdi el Gedîol-Dâkhlao, E part of the oasis below Balat and Ismant (Smint), Walter s.n. (K). **Ethiopia**. Oromia: Lake Alemaya (Haramaia), Burger 3607 (PRE, US); South shore of Lake Alemaya, 35 km on the road from Dire Dawa to Harar, de Wilde 4789 (B, MO); NW shore of Lake Langano, Ash 1377(K, MO) and 2644 (K); Lake Awasa, ca. 20 km SW of Shashamane, de Wilde & de Wilde-Duyfjes 7016 (B, MO). **Germany**. Wollkammerei, Kuhbier s.n. (B). **Indonesia**. Java: Horsfield s.n. (BM). **India**. Kerala: Vembanad Lake near Alleppey, Vencoba Rao 4061 (K). West Bengal: Salt Lake, Calcutta (Kolkata), Clarke 21605 (BM). **Iraq**. Basrah: Basra[h], Omar & Omar 35105 (K); Ambadi Basra, Graham 535 (BM); Garma inter Basra et Qurna, Rechinger 15815 (B, K, M, S, UC, US, W). Al-Chibayish: Al-Chibayish [as Chabaish], Thamer 50106 (K). Unknown: Maqil, Rechinger 8451 (M, W); Kabajish, Springfield 12545 (US). **Italy**. Venice: Venice, Eggert s.n. (NY, KSC). **Kenya**. Coast: Malindi, Banks of Sabaki Riv., Bogdan 2605 (UC). Rift Valley: Mile 5 from the Sobo Rd.-Galana Riv. turnoff, Greenway & Kanuri 12759 (P); West Shore of Lake Baringo, Bogdan 4102 (P, US); W side of Lake Baringo, Burney & Burney B66 (NY); Masai Distr: N end of Lake Natron, SW of Shombole Mt., Milne–Redhead & Taylor 7010 (B, K). **Madagascar**. Mahajanga: Ambohimena (Marovoay, Ouest), Bosser 8357 (P); Marovoay, Perrier de la Bâthie 11216 (P). Toliara: Antanimora (Pce. du Fort-Dauphin), Decary 4636 (P); Vohitany, Bosser 15614 (P); Vohitany, Bekily, Bosser 15686 (P); Ambovombe, Decary s.n. (P); Beloha-Ranolava (Tsihombe), Zolotarevsky 2623–37 (P). **Malawi**. Polambe plain near Rjalo Island, Jackson 582 (K, US); Dwangwa Riv., Jackson 1111 (K); Fort Johnston, Jackson s.n. (P). **Mozambique**. Inhambane: San Sebastian, Mogg 29142 (PRE). Maputo: Maputo [as Lourenco Marques], entre a Costa do Sol e Vila Luiza, a 5 km da vila, Fidalgo de Carvalho 1446 (MO); Maputo [as L. Marques], estrada para a Costa do Sol, Balsinhas 656 (B); Inhaca Island, 23 mi.E of Lourenco Marques, Mogg 29850 (PRE); Inhaca Island, Mogg 29799 (MO). Sofala: Beira, Hitchcock 24410 (US). Myanmar. Bago: Bago [as “Pegu”], Kurz 1105 (K). **Namibia**. Caprivi: Asheshe area, ca. 2 km S of Asheshwe village, Ward 36 (PRE). Hardap: Gibeon: 63 km N of Tses along Marietal-Keetmanshhop Hwy, Davidse & Loxton 6342 (BRI, MO, PRE, US); Farm Helmeringhausen, Kinges 2198 (PRE); NNW of Maltahöhe on Rd. D850 to Büllspoort [Bullsport], Smook 9338 (US). Karas: Along Hwy B4, ca. 62 Road km E of Goageb, ca. 40 Road km SW of Keetmanshoop, along Fish Riv., ca. 100 m N of bridge over river, just downstream from cement irrigation channel over river, Snow & Burgoyne 7176 (K, KSP, MO, PRE, WIND); Hardap Region, 34.4 road km N on C14 from junction of C14/C13, between Helmeringhausen and Maltahöhe, Snow & Burgoyne 7196 (K, KSP, MO, PRE, WIND); 27 km W of Maltahohe on Rd. no. 36 to Sesriem, Ellis 4779 (PRE). Kohmas: Neudam Exp. Farm, Van Vuuren 1033 (K, PRE); In vlei at Ludwein near Windhoek, Liebenberg 5074 (UC). Kunene: Etosha Game Park, 11 mi.N of Okandeka waterhold, Tinley 1202 (K); Etosha N.P., 8 km nürdlich von Okaukuejo am Weg nach Okondeka, Giess & Müller 13960 (M, PRE); Estosha Game Park border, Ekuma Riv., Tinley 1140 (K). Ohangwena: Ovamboland Nature Reserve, de Winter & Giess 6916 (K) Okavango: Klein Omuramba 5 mi.E of Runth, de Winter 4076 (K). Omaheke: Farm Omupanda, 8 km E of Hochveld, Gibbs Russell & Smook 5356 (K) and 5357 (PRE). Oshana: Ondangua, Soini s.n. (H). Oshikoto: Oniipa, Soini s.n. (H). Otjozondjupa: Sonop Research Stn, Strohbach 2309 (K); 35.3 mi. SW of Taumeb on road to Otavi, de Winter 2916 (K); 27 km W of Maltahohe on Rd. no. 36 to Sesriem, Ellis 4779 (PRE); Farm Tjo Noord, 25 km E of Kalkveld, Gibbs Russell and Smook 5248 (K); Waterberg Omuramba, Volk 1420 (US). Region unknown: Ab loco, Felmer 456 (US). Niger. Zinder: Zinder, Hagerup 576 (GH, S). Region unknown: Point Sabonjari, Boudet 5040 (P). **Nigeria**. Bornu: North Eastern State, Baga Lake Chad Border, Wit et al. 1474 (MO); N of Mardufuri, de Leeun 1896 (P). **Pakistan**. Punjab: ca. 10 mi. from Lahore on way to Baidyan, Qaiser 3551 (K, TAES). **Papua New Guinea**. Western: Mainland, opposite Daru Island, Brass 6065 (BM, GH, NY, US); Near Bula Village, at mouth of Morehead Riv., Pullen 7029 (A, K, US); near Mabaduan Hill, 50 km WSW of Daru, Paijmans 1501 (CANB). **Philippines**. Luzon: Manila, Merrill 554a (B, BM, K, PR, UC, US); Hot Springs, Los Baños, Gates 6348 (KSC); Los Baños, Merrill 5104 (K, US, W); Los Baños, Williams 2027 (K, NY, US); Manila, Santos 27 (US); Barrio Tanuk, Buluan Marsh, SE bank of Buluan Lake, Vera Santos 5969 (B, US). Pampanga: Masantol, “Fishery Student” s.n. (PNH). **Saudi Arabia** [provinces unknown]; km 106, Makkah by-pass, Collenette 3938 (K); Al-Taif, Al-Hada region, ca. 20 km from Al-Taif, Fayed 1151 (K). **Senegal**. Dakar: Hann, Adam 14090 (MO); Rufisque, Rte de Mbour, Adam 2273 (MO); Dakar Bongo, Audru 2783 (P); Rufisque, Route de M’Bour, Adam 2273 (MO). Kaolak: Ravin des Voleurs, Berhaut 3784 (P); Kaolak, Trochain 4035 (P); Kaolak, Berhaut 626 (P). Louga: Menguilé, Ferlo, Mosnier 2405 (P). Saint-Louis: St. Louis, Berhaut 2813 (P); St. Louis, Khor Marshes, Hepper 3613 (P); Tiguet, Audru 2938 (P); N’Diael, Audru 2982 (P). Unknown: Mbidjem, rive W du Tanma, Raynal 6559 (P). **Singapore**. Perak, Ag. Officer 35646 (BM). **South Africa**. Cape Districts (Northern, Western, Eastern): Beaufort West Distr., Farm Layton, Shearing 267 (K); Dist. Swellendam, Nat. Bontebok Park, Liebenberg 7204 (K); Near Riversdale, Muir 2859 (K); Gordonia, Kalahri Bemgsbok N.P., Leistner 1017 (K); Ft. Beaufort, between Ulster and Mooiriver, Smook 4033 (K); Dist. East London, Bulura Mouth, Eacocks 15785 (K); Dunswart, near Johannesburg, Moss 13959 (K); Duivenhoksrivier, 10 km NW of Vermaaklikheid on road to Heidelberg, Davidse 33779A (MO); Springbok Dist., Namaqualand, top of Wildeperdehoek Pass SW of Springbok, Goldblatt 2820 (MO); Piquetberg, Liedenberg 4266 (UC); Upington, Gordonia Distr., Liebenberg 4163 (P, UC); Buffelsrivier valley between Pedroskloof and Bobbejaanhoek on road to Rooifontein, Davidse 33309 (MO); 10 km S of Humansdorp on road to Cape St. Francis, Davidse 33626 (MO); NW...on Holbakrivier in small port, Smook 3858 (K, US); Carnarvon, Acock 1737 (US); Cape of Good Hope at Douglas Heights, NW of Grahamstown, Godfrey and Storey SH–1362 (US); Hardenbeck Riv. dam cistern on Excelsior Farm, Hugo 2286 (MO, US); Farm Lemoenkop 337, ca. 2 km N of Lemoenkop farmhouse, just S of True Beacon 25 in drainage channel, Le Roux & Lloyd 94 (PRE); Nat. Kalahari Gembsbokpark, Liebenberg 7080 (PRE); Obobogorap, ca. 120 mi. NW of Upington, Leistner 1781 (PRE); Nat. Bontebok Park, Liebenberg 7204 (PRE); Kleinemonde, Ward 8897 (PRE); (Ubombo) Lower Mkuze floodplain, Ward 8785 (K); Paterson, 2 km off main road to Addo, Smook 3768 (K, PRE); 53 km SE of Graaff Reinet on small running stream below homestead, Smook 3896 (K, PRE); Karoo N.P., Doornhoek, Bengis 462 (PRE); Grootfontein, Theron 701 (PRE); Beaufort West Distr., Farm Layton, Shearing 380 (PRE); Kalahari Gembsbok Park, Mata–Mata, Van Rooyen and Bredenkamp 115 (CM); Kaffraria, Burtt–Davy 12786 (PRE). Free State: 8 km from Luckhoff on road to Jacobsdal, Smook & Gibbs Russell 2456 (K); Boskop (363) langs Petrusburg–Boshofpad, Muller 1304 (K); Small pan just S of Mayhem Pan, Edwards 4159 (K); 11 km from Petrusburg on road to Boshof, Smook & Gibbs Russell 2498 (K, PRE, US); 23 km from Petrusburg on road to Boshof at Modder Riv. Bridge, Smook & Gibbs Russell 2501 (K, PRE, US); 5 km from Bultfontein on road to Brandfort, Ellis 3653 (PRE); Bloemfontein, du Preez 1884 (PRE); ca. 10 km W of Odendaalsrus, Smook 6501 (BRI); 25 km from Luckhoff on road to Phillipolis, Smook 2879 (PRE); 5 km from Bultfontein on road to Brandfort, Smook 2728a (PRE); Distr. Harrismith, Mont Pelaan, Ferreira F212 (P); Mont Pelaan, Harrismith, Ferreira 212 (P). Gauteng: Benoni, Bradfield T371 (PRE). Kwazulu-Natal: Bridge over St Lucia Estuary, Ellis 3410 (PRE); St. Lucia Lake, Hlabisa, Zululand, Liebenberg 5903 (UC); South Coast, Pole–Evans 778 (PRE); Bridge over St Lucia Estuary, Ellis 3410 (PRE); Falce Bay Park, Ward 7724 (PRE); Bartlow Combine bay Hluhluwe Wildtuin, 24 km van Mkuzi op pad na Mtubatuba, draai na Weste vir 20 km, du Toit 660 (PRE). Limpopo: Kruger N.P., Malongafontein, Ellis 1908 (PRE); 13 km SE of Chrissiesmeer, Smook 4905 (K); On Farm Mosdene, Distr. Naboomspoint, Liebenberg 4424 (UC); Dist. Bloemhof, SA Lombard Nature Reserve, Leistner 78 (K); Kruger N.P., Malongafontein, Davidse 5875 (BRI, K); Kruger N.P., Malongafontein, Ellis 1908 (PRE); Kruger N.P., Shingwidzi rest camp, Oakes 1455 (US); Kruger N.P. 19 mi. NE of Skukuza, De Winter and Codd 560 (BM, K); Kruger N.P., near Malonga Spring, Oakes 1483 (US); Wanetzi, Van der Schyff 508 (PRE); 23 mi. SE of Punda Maria, De Winter & Codd 674 (BM, K, PRE); Mosdene farm Naboomspruit [Mookgophong], Germishuizen 42 (PRE); Naboomspruit, Schweickerdt 1791 (BM); 2 mi. N of Rust der Winter Dam, De Winter & Codd 248 (BM, CANB). Mpumalanga: 3 km from Lake Chrissie near the shore of the lake, Loxton 398 (PRE). North West: Beneath spillway of small (ca. 5 m tall) earthen dam, located below N side of road ca. 50 m, on N14 Hwy, 4.1 road km E of Kuruman, Snow & Burgoyne 7108 (K, MO, PRE); 11 mi. NNE of Lichtenbrug on road to Koster, Scheepers 1495 (K, PRE); W of Biessiesvlie, Farm Holfontein, Smook 6628 (PRE, RSA); Knopfontein 101 IP, near Coligny, Pan no. 7, Allan 117 (PRE); Knopfontein 101 IP, Pan no. 7, Allan 134 (BRI, PRE); Bloemhof, Lombard Nature Reserve, Gemsbokpan, Leistner 2108 (K, M, PRE); Bloemhoef, Lombard Nature Reserve, Jeffers 400 (PRE), Lombard Nature Reserve, Oeistner 78 (BM). Unknown: Malathlopanga, Van der Schyff 5658 (M, K, PRE); W of Biessiesvlei, Farm Holfontein, Smook 6228 (PRE, US). **Sri Lanka**. Jaffna: Near Ampan, Clayton 5236 (CANB, K, TAES, US); ca. 4 mi.NW of Jaffna, along the coastal road, Davidse & Sumithraarachchi 9119 (BRI, CANB, K, MO, TAES, US); Keerimalai to Point Pedro, Clayton 5201 (CANB, K, TAES, US); Near Chavakachcheri, Clayton 5255 (CANB, K, TAES, US). Mannar: Mantai, Davidse & Sumithraarachchi 9174 (BRI, CANB, K, MO, NY, TAES, US). Puttalam: Puttalam, sea front, Clayton 5664 (CANB, K, TAES, US); Puttalam, sea front, Clayton 5665 (US); Trincomalee Dist., ca. 5 mi. due S of Tamaivillu, Davidse 7581 (CANB, US). Province Unknown: ca. 4 mi. NE of Hambantota, marker 154/4, Gould 13458 (US). **Switzerland.** Skåne, Lackalänge, Furuland, Blom s.n. (US). **Taiwan**. Little Quemoy, Chuang 4459 (UC, US). **Tanzania**. Arusha: Sanya Plain, Arusha–Moshi, Leippert 6455 (M); Ngorogoro Crater, Greenway & Kanuri 12535 (P); Ngorogoro Crater floor, Gorigor swamp, western end, Raynal 19521 (P); Ngorogoro Crater, Heady 1517 (UC); Tituski Riv. near Lake Magadi, Greenway & Turner 10043 (US); Nyumba ya Munga Lake, at Magadini fishing village, Mhoro & Backéus 2025 (MO). Mara: Musoma Distr., Mbalageti Riv., Greenway 9027 (B, PRE). Mbeya: Ikoga, 100 mi. SW of Iringo, Heady 1871 (MO, UC). Rukwa: Lake Rukwa, Bullock 3439 (B); Near Kampunda, Bullock 3605 (US). Tanga: Mkomazi Valley, Semsei 3964 (P). Province Unknown: Lake Rukwa Extension, Michelmore 738 (US); Milepa, Lake Rukwa, Michelmore 1418 (BM, MO); SW Serengeti Plains, headwaters of the Mbalangeti Riv., Greenway et al. 13183 (K, MO); Northern Province, Scout 618 (NY). **Thailand**. Paknam, Sørensen et al 2038 (A, B, K, P); Ab. loc., Kerr 16107 (BM, K); Hua Hiss, Kerr 16126 (BM, K); Tachin, Kerr 8975a (K); Palanam, Kerr 20441 (K, US); Paknam, Sørensen et al. 98 (K); Paknam, Sørensen et al. 2035 (B, K); Tacheen, near Bankok, Marcan 1812 (BM); Kao Tao, Kerr 16183 (BM, K). **Uganda**. Butiaba, Lake Albert, Greenway & Eggeling 7047 (K). **United Kingdom**. Barming, Mason J/67/K1 (K); Blackmoor, N. Hants, Lousley 1348 (BM, K); Charlton, Webster 8899 (BM, K) and Webster 2053 (K); Blackmoor fruit farm, grown on at Ware, Herts, Hanson 162 (BM); Blackmoor, Wurzell 1086 (MO) and Lousley 2914 (K) and Lousley 3008 (K) and Webster 7005 (K). **United States of America**. California: Kern Co., Isabella Lake bed, old Kern Riv. channel ca. 1/4 mi.S of of the old Borel Canal Bridge, Twisselmann 6491 (MO). Santa Barbara Co., San Jose Ck. at Southern Pacific RR crossing, Goleta, Pollard s.n. (MO). Yolo Co., 2 mi.SW of Knight’s Landing, Rose 69041 (FSU, GH, MICH, MO, NY, RSA, SMU). South Carolina: Berkeley Co., Santee Wool Combing Mill, Jameston on SC Rte 45, Ahles & Haesloop 53442 (NCU); ibid., Ahles & Haesloop 47048 (NCU); ibid., Ahles & Haesloop 52773 (NCU); ibid., Ahles & Haesloop 43027 (NCU). Florence Co., Wellman Wool Combing Mill, N of Johnsonville on SC Rt. 41, Ahles 42895 (MO, USCH); ibid., Ahles 42984 (NCU), Ahles & Haesloop 30873 (NCU), and Ahles & Haesloop 30876 (NCU), and Ahles 42895 (OKL). **Yemen**. Rada, Westinga 3 (K); just N of Rada, Wood 3207 (BM, K). **Uruguay**. San José: Rio San José, Puerto Tres Bocas, Rosengurtt B–7316 (P). **Zambia**. Kabwe: Kakumbasa Riv., Verboom 3185 (K). Senanga: Barotseland, Mulonga and Siloana plains, Verboom 1125 (BM, K). Districts unconfirmed: Kafue flats, Magabuka, Astle 1404 (K) and Greenway 6228 (K); Monze, near Lochinvar, Rensburg 2707 (K); Lochinvar estates, Trapnell 1112 (K); Fort Jameson, Van Rensburg 2135 (B); Kafue N.P., Mitchell 24/42 (B, BM); Muckle Neuk, 12 mi.N of Choma, Robinson 587 (M); 12 mi. N of Choma, Robinson 2857 (M, P); Njeju Game Reserve, Verboom 749 (BM, K); Kakumbasa Riv., Kabwe rural, Verboom 3185 (MO); Lake Chisi, E of Mwera wa Ntipa, Michelmore 428 (K). **Zimbabwe**. Gwampa Forest Reserve, Goldsmith 10/56 (B, BRI, CANB, NY, P, UC, US); Lower Sabi District, Rattray 1235 (BRI, P, US); Shashi Irrigation Scheme, 12 mi. SE of Tuli, Drummond 5924 (BM); Sentinel Ranch, some 12 mi. N of Pazhi–Limpopo confluence, Drummond 5982 (BM); Beaufort West, Bleak House Farm, Gibbs Russell et al. 370 (MO); Ft. Beaufort, 3226 DD Alice, “Woodstock” H. E. Matthews’ farm, Giffen 1624 (MO).

#### Diplachne fusca subsp. muelleri

Taxon classificationPlantaePoalesPoaceae

(Benth.) P.M. Peterson & N. Snow. Ann. Bot. 109: 1327. 2012.

Figures 3 , 4

Diplachne muelleri Benth., Fl. Austral. 7: 619. 1878. Leptochloa muelleri (Benth.) Stace, Watsonia 18: 413. 1991. Leptochloa fusca subsp. muelleri (Benth.) N. Snow, Novon 8: 78. 1998. Diplachne fusca (L.) Kunth var. muelleri (Benth.) J.M. Black, Fl. S. Austr. 1: 76. 1922. Diplachne fusca (L.) Kunth var. muelleri (Benth.) P.M. Peterson & N. Snow, Phytoneuron 2012-72: 2. 2012, isonym, nom. inval.

##### Type.

AUSTRALIA. Northern Territory: Charlotte Waters, Giles s.n., Herb. Wm. Munro (lectotype, designated here: K [K000899903, specimen on right side of sheet]!; isolectotypes: K!, US [734098]!).

##### Description.

Plants annual. Culms 15–100 cm tall, 1.5–3.8 mm wide at base, round, cespitose, erect or rarely geniculate below and rooting at lower nodes, usually branching (sometimes profusely); nodes glabrous; internodes 1.5–18 cm long, soft, hollow. Leaf sheaths longer or shorter than internodes, glabrous on sides and margins; ligules (1.5–)5–7.5 mm long; blades (3.5–)7–25 long ×0.2–0.5 cm wide, scabrous above, scabrous to nearly glabrous below. Panicles 5–35 ×2–7 cm, partially inserted below with 5–16 branches; the branches (0.7–)2–9(13) cm long, alternate or sometimes subopposite, ascending to erect, rigid, glabrous to minutely scabrous, axils glabrous. Spikelets 10–14 mm long, mostly imbricate; florets 8–13; callus glabrous; lower glumes (2.4–)3.3–4.7 mm long, narrowly ovate or somewhat irregularly asymmetric, glabrous, obtuse to acute apically; upper glumes 4–5.4 mm long, ovate, glabrous, obtuse and sometimes bifid; lemmas 4.7–5.8 mm long, 3-nerved, ovate, pale or smoky white, the lateral nerves pronounced and excurrent or not, sericeous to densely velutinous on lower 1/3–2/3 of lateral nerves, the midnerve usually somewhat less pubescent, apex acute, often bifid, mostly awnless or mucronate; paleas subequal to slightly exceeding lemma, obovate to narrowly ovate, sericeous to velutinous along lower 1/2 to 2/3 of nerves (hairs often diverging widely at maturity); apex obtuse to acute. Stamens 3; anthers 0.5–0.7(–1.0) mm long, yellow. Caryopses 1.6–2.4 ×1.0–1.1 mm long, obovate in hilar profile, depressed obovate in transverse section, hilar groove lacking, smooth, brown; pericarp weakly adnate to the endosperm.

**Figure 3.**
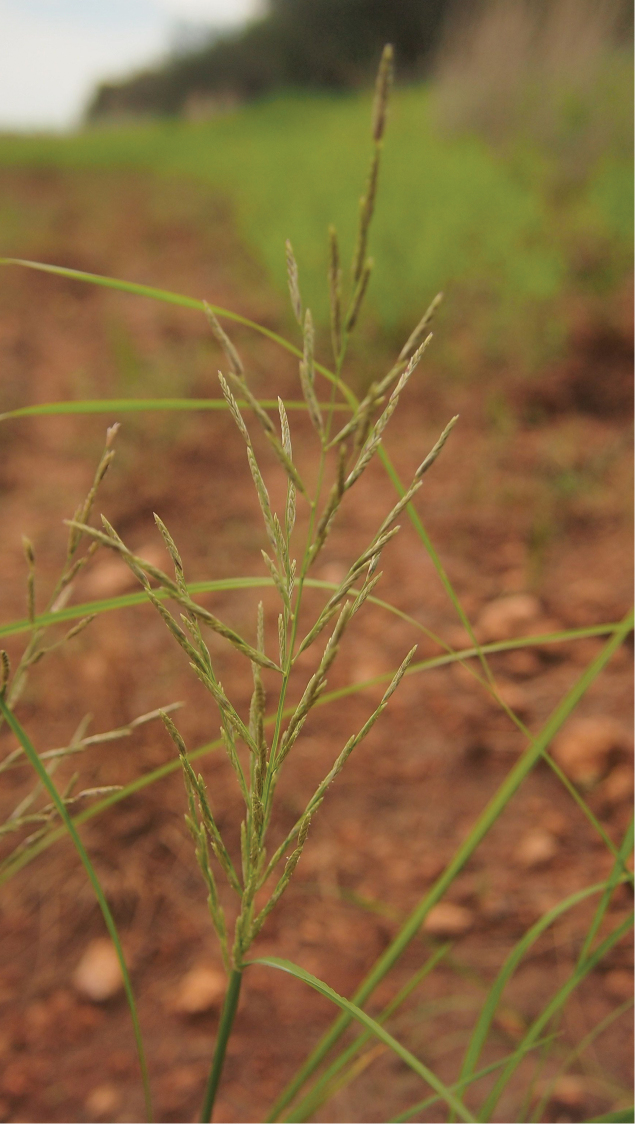
Inflorescence of Diplachne fusca subsp. muelleri (no voucher). Image by Mark Marathon (CC-BY-SA-4.0).

**Figure 4.**
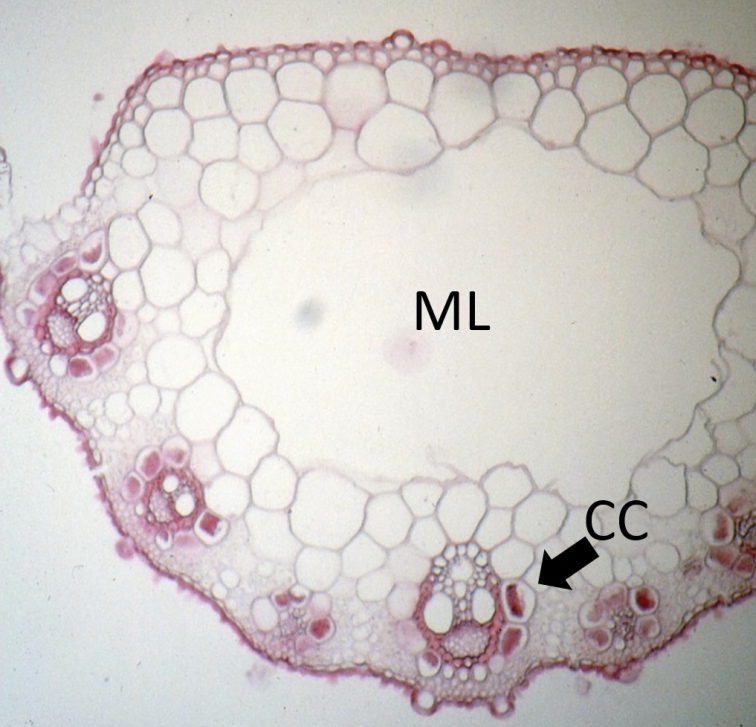
Cross section of leaf of D. fusca subsp. muelleri (Badman 675) showing midrib (central area), midrib lacuna (ML) and vascular bundle with centripetal chloroplasts (CC) of the mestome sheath.

##### Leaf anatomy.

**Figure 5:**
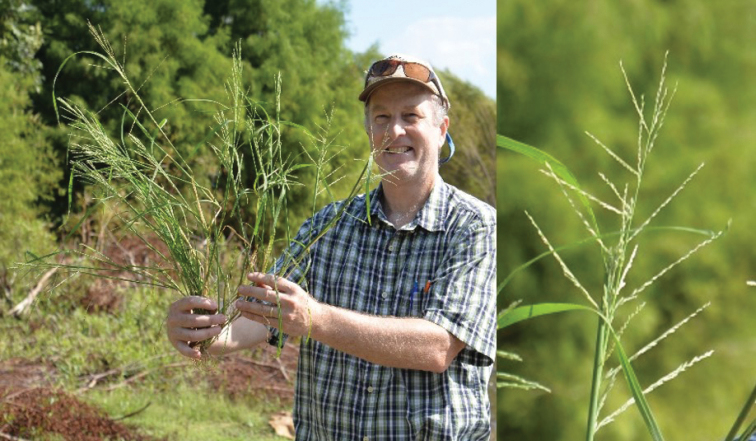
Typical growth form (left) of Diplachne fusca subsp. fascicularis (Montgomery Co., Kansas; Snow 10978 [KSP]). As typical for the subspecies, it has fewer, longer, and more widely spaced panicle branches (right) than L. f. subsp. uninervia, with one or more of the lower panicle branches enclosed in the uppermost leaf sheath.

Voucher: Badman 675 (CANB) (Fig. 5). Description as per D. fusca subsp. fascicularis.

##### Stem anatomy.

Not examined.

##### Choromsome number.

Unknown.

##### Phenology.

Flowering January through October.

##### Distribution.

**Native**: Widespread across inland Australia, particularly the central regions; wet areas such as streambanks, around boreholes, and swamps, in a variety of soil types (TDWG: WAU-WA, NSW, VIC, QLD-QU, NTE.) Elevation from near sea level, upper elevation unconfirmed. **Non-native**: TDWG: SWI; not confirmed.

##### Conservation status.

Least Concern (IUCN 2012) due to its widespread distribution across Australia.

##### Etymology.

Named after German-Australian botanist Ferdinand von Mueller (1825–1896).

##### Vernancular names.

Mueller sprangletop; Mueller beetlegrass, Sugar Grass (in the Maranoa District of Queensland, Australia).

##### Comments.

Diplachne fusca subsp. muelleri is most easily recognised by the relatively narrow panicles, which are largely inserted at their bases and the presence of inflorescences at most points of branching on the culms. The mature lemmas often have a dark spot at the base at maturity, are relatively broad, flattened and the dense trichomes along the nerves typically spread widely at maturity.

Diplachne fusca subsp. muelleri most closely resembles D. f. subsp. fascicularis by virtue of the lower inflorescence branches that typically remain inserted at the bases, especially from lateral inflorescences. However, the upper glumes are generally much broader than the primarily North American subspecies.

Another occasional trait is a pronounced magenta to purplish mottling of the leaf sheaths (e.g. Carolin 6300 [US, K], Weber 8769 [US, W]), which typically is absent in D. fusca subsp. fusca, another native Australian subspecies, but which recurs sometimes in the American subspecies fascicularis. The relatively short spikelets of Lazarides 4381 (CANB), Chippendale 2036 (CANB), Hubbard & Winders 6263 (B, CANB), coupled with inserted, narrow panicles, suggest hybridisation between between D. fusca subsp. muelleri and *fusca*.

This subspecies is a relatively short-lived annual, whose relative abundance is often associated with the vagaries of water availability, particularly along watercourses and bores (=wells). The label of Symon 5617 (CANB, US) states the specimen grew relatively close to the hottest water emerging from the bore (ca. 110°F / 42°C). Cattle will graze the foliage and pastured horses eagerly consumed freshly collected specimens when offered (Snow pers. obs. 1996).

##### Specimens examined.

**Australia**. New South Wales: Tero Ck Station, Martensz G 308 (MU). Along road between White Cliffs–Tero Ck. Station, Martensz 4494 (CANB); Near Goonery Bore, 65 mi.E of Wanaaring, Moore 5785 (BRI,CANB); ca. 35 km NW of Milparinka, Hawker’s Gate Rd., Jacobs 3510 (BRI); 39 km from Brewarrina towards Collerina, Dunlop 933 (CANB); 11 km from Brewarrina towards Collerina, Dunlop 915 (CANB); “Mt. Mulyah”, ca. 50 mi. NW of South, Moore 5010 (CANB); Calindary Station, Libke 4479 (CANB); Tero Ck. Stn., Martensz 2320 (CANB). Northern Territory: Burt Plain, 33 mi.N of Alice Springs, Must 487 (BRI, CANB); Ruby Gorge, Hale Riv., ca. 70 mi. ENE of Alice Springs, Beauglehole 20735 (BRI); Heavitree Range, Ormiston Gorge, Beauglehole 45280 (BRI); Twin Bore, Todd Riv. station, Latz 812 (BRI); 1 mi. S Long waterhole, Coniston Station, Latz 1192 (BRI, CANB); Busrt Ck., 36.5 mi. N of Alice Springs, Nelson 1653 (BRI, K); 24 mi. S of Barrow Ck., Perry 5352 (BRI, K); Heavitree Range, Ormiston Gorge, Beauglehole 45280 (BRI); North West Simpson Desert, Latz 4642 (CANB); Twin Bore, Todd Riv. Station, Latz 812 (BRI); Burt Plain, 33 km N of Alice Springs, Must 487 (BRI,CANB); Andado, Buckley 1280 (CANB); Palm Valley, Chippendale 2669 (BRI, CANB); ca. 8 km SW of Mt. Conner, Carr & Beauglehole 1959 (CANB); Coolata Springs, 30 mi. SSE of Mt. Ebenezer Station, Lazarides 6194 (BRI, CANB, K, US); Emily Gap, 10 mi. E of Alice Springs Township, Lazarides 5329 (BRI, CANB, MO, US); 1/2 mi. S of Yambah Station, Lazarides 5192 (CANB); Petermann Bore, Tempe Downs Station, Henry 558 (CANB); Livingstone Pass, Petermann Ranges, Carolin 6300 (US, K [n.v.])10 mi. SW Alice Springs, Swinbourne 792 (CANB); Mt. Denison Station, Martin 40 (CANB); ca. 13 km SSW of Alice Springs, Pullen 10525 (CANB); 24 km S of Barrow Ck. Township, Perry 5352 (CANB, US); 28 mi. NNE of Alice Springs, Perry 3340 (CANB, K); 5 mi. S Alice Springs, Paige s.n. (CANB); Bloodwood Bore (No. 25) 46 km SW Brunette, Must 516 (CANB); 5 km E Old Coniston Homestead, Latz 1170 (BRI, CANB); 8 km NNE of Willowra Homestead, Latz 1250 (CANB); 65.5 mi. NNW of Old Andado Homestead, Beauglehole 27805 (BRI); 9 mi. W of Old Andado Homestead, Beauglehole 27998 (BRI); 8 km SW of Mount Conner, Beauglehole 45738 (BRI); George Gill Range, Kings Canyon, along Kings Ck., Beauglehole 20294 (CANB); Alice Springs Airport entrance, Nelson 2067 (BRI); 28 mi. NNE of Alice Springs Township, Perry 3340 (BRI); Deep Well Rd., 13 mi. S Alice Springs, Nelson 1832 (BRI); Palm Valley, Beauglehole 10351 (BRI); Palm Valley, Chippendale 2036 (CANB); Ruby Gorge, Hale Riv., ca. 70 mi. ENE of Alice Springs, Beauglehole 20735 (BRI); 1 mi. S of Long Waterhole, Coniston Station, Latz 1192 (BRI, CANB). Queensland: Burke Dist., Sandhurst Bore, Millungera Station, Blake & Lazarides 4783 (K, MEL, NSW, US). Darling Downs Dist., 16.5 km SE of Cunningham Hwy on Glenarbon Rd., NE of Texas, Snow & Simon 7311 (BRI, MO). Gregory North Dist., W side of Lake Phillipi, Kamaran Downs Station, ca. 60 km W of Bedourie, Edmunds KN2 (BRI); 30–35 mi. S of Bedouire, Blake 12307 (BRI, K). Gregory South Dist., Coochie bore, 55 km NW of Birdsville, Edmnuds AD62 (BRI); Earlstown between Quilpie and Windorah, Blake 5459 (BRI), Mulligan–Eyre Survey, Gasteen 11 (BRI); Between Betoota and Birdsville, Peart 1720 (BRI). Maranoa Dist., Boatman Station; “Bluebush Swamp”, Everist 2857 (CANB, US). Mitchell Dist., Near Lochnagar, Blake 10322 (BRI, CANB, K, NSW); Geera, E of Barcaldine, Blake 10371 (CANB, K); Walrus V, Site G215, just S of Lake Mueller, ca. 29 km NE of Aramac on Lake Dunn Rd., McDonald 2672 (BRI). Warrego Dist., ca. 14 km N of Thargomindah, Fairfax 1273 & Kemp (BRI); On Paroo Riv. channels, 7 km N of Hungerford, Eberson E202 (BRI). “Gilruth Plains”, Allen 269 (CANB); “Curragh” Station, near Cunnamulla, Hubbard & Winders 6263 (B, CANB, NY); Eulo, White 11575 (K). South Australia: in the hot water from Murnpeowie flowing bore, Symon 5617 (B, CANB, K, US); Northern margin of Margaret Ck., ca. 30 km W of Coward Spring Railway Station, Weber 8769 (US, W); Between Camp and Rotten Swamp ca. 12 km NW of Quinyambie Homestead, Whibley 3587 (NY); Region 2, Lake Eyre Basin, Margeret Ck., near Curdimurka, Badman 492 (CANB); 16 km NE of Marree on the Birdsville Track, Badman 730 (AD, BISH); 1 km S of Anna Ck. HS, 16 km W of William Ck., Badman 1263 (BRI); Region 2, Lake Eyre Basin, N Lake Talinnie, O’Malley 374 (BRI); 34 km S of William Ck., Beauglehole 28112 (BRI); ca. 40 km W of Marree and 9 km in a southerly direction off the main road to William Ck., Weber 9671 (BRI); Lake Eyre South, Sales and lower slopes of sandhills S of the lake, Badman 488 (CANB); Region 4: Gairdner–Torrens, Bates 16944 (CANB); Region 2: Lake Eyre Basin, Callanna Ck., 15 km W of Marree, Badman 378 (CANB); Region 2: Lake Eyre Basin, 4 km SE of Coward Springs, Badman 675 (CANB); Region 2: Lake Eyre Basin, km SE of Nunns Bore 28 km SE of William Ck., Badman 1876 (CANB); N of Marree, Lake Harry, McKean APG3 (CANB); Clayton Riv.... 50 km (ca. 30 mi) NE of Marree, Lothian L2011 (B); Near Queensland border, ca. 15 km NE of Innamincka, Whibley 2480 (B); Region 2: Lake Eyre Basin, Marree–Oodnadatta Rd., 42 km by road NW of William Ck., Donner 9870 (MEL); Lake Eyre Basin, 5 km E of Strzelecik Ck crossing, Williams 8023 (AD, BISH). Victoria: Near Mt. Everard, Giles s.n. (MEL). Western Australia: ca. 20 km E of Mulga Downs Headquartes, Mitchell PRP263 (BRI); Bore, 6.4 km W of Mongral Downs, Dunlop 2119 (BRI); 28 mi. E of Windidda, Eremean Prov., Speck 1269 (CANB, US); Donkey Well, Yoothapina Station, Cranfield 5557 (CANB); Hadji Well, near Lake Way, Craven 5392 (CANB); Bore, 6.4 km W of Mongral Downs, Dunlop 219 (BRI); Bore, 6.4 km W of Mongral Downs, Dunlop 2119 (BRI); 28 mi. E of Windidda, Speck 1269 (CANB, US); Donkey Well, Yoothapina Station, Cranfiedl 5557 (CANB): Hadji Well, near Lake Way, Craven 5392 (CANB). **Switzerland**. Derendingen, Probst 8902 (US, n.v.).

#### Diplachne fusca subsp. fascicularis

Taxon classificationPlantaePoalesPoaceae

(Lam.) P.M. Peterson & N. Snow, Ann. Bot. 109: 1327. 2012.

Festuca fascicularis Lam., Tabl. Encycl. 1: 189. 1791. Diplachne fascicularis (Lam.) P. Beauv., Ess. Agrostogr. 81, 160, pl. 16, f. 9. 1812. Cynodon fascicularis (Lam.) Raspail, Ann. Sci. Nat., Bot. 5: 303. 1825. Festuca aquatica Bosc ex Roem. and Schult., Syst. Veg. 2: 615. 1817. Nom. inval. pro syn. Diplachne fascicularis P. Beauv. Diplachne aquatica Bosc ex Roem. and Schult., Syst. Veg. 2: 615. 1817. Nom. inval. Leptochloa fusca subsp. fascicularis (Lam.) N. Snow, Novon 8: 78. Diplachne fusca (L.) Kunth var. fascicularis (Lam.) P.M. Peterson & N. Snow, Phytoneuron 2012-72: 2. 2012.
Festuca polystachya Michx. Fl. Bor. Amer. 1: 66. 1803. Leptochloa po*lystachya* (Michx.) Kunth, Révis. Gramin. 1: 91. 1829. **Type.** U.S.A., Illinois, A. Michaux s.n. (holotype: P [P00680135]!; isotypes: GH [00062448]!; P [P00680134]!, [P00680136]!, [P00680137]!, [P00680138]!, [P00740207]!, [P00740209]!, [P00740210]!).
Festuca texana Steud., Syn Pl. Glumac. 1: 310. 1854. **Type.** United States of America, Texas. T. Drummond 387 (holotype: P [P032675]!). Snow (1997a: 253) inadvertently referred to the holotype as a lectotype and annotated it as the latter, although the specimen is marked “Herbarium Steudel”.
Festuca thouini Steud., Syn Pl. Glumac. 1: 311. 1854. **Type.** West Indies, Ins. Antillae in Domingo, A. Thouin (holotype: P [P032674]!, fragment US! (US–78820)). This specimen (see previous) also was annotated incorrectly as a lectotype (Snow 1997a: 253).
Uralepis composita Buckley, Proc. Acad. Nat. Sci. Philadelphia 1862: 94. 1862. **Type.** United States of America. New Mexico, Woodhouse s.n. (holotype: PH [PH00028562]!).
Diplachne tracyi Vasey, Bull. Torrey Bot. Club 15: 40. 1888. Leptochloa tracyi (Vasey) Beal, Grass. N. Amer. 2: 436. 1896. **Type.** United States of America. Nevada. Reno, 1887, SM Tracy (holotype: US! [US00133612]!; isotypes: GH [00023585]!; NY [NY00019502]!, [NY00019503]! [NY-1144032]!, [NY-1144033]!). Uncertainty has surrounded the correct type citation (Snow 1997a: 253). The protologue indicated a collection of Tracy in Reno, which is housed at US and cited above. The number 216 on the label of the US specimen was added by a later worker. Hitchcock (1939: 613), evidently working from Leptochloa tracyi (Vasey) Beal, Grass. N. Amer. 2: 436 (1896), believed lectotypification was necessary given the citation of two collections (of Tracy and Palmer and each of these assigned the number 619 by some later worker) in the work by Beal. However, Vasey’s description included a description of the newly proposed species, in addition to citation of a specimen collected by Tracy and made no reference to Palmer’s collection, so lectotypification was unnecessary. Zanoni ([NY-1144033]) in 2010 also made reference to this on an annotation slip.
Diplachne procumbens Arechav., Anales Mus. Nac. Montevideo 1: 414. 1894. Diplachne uninervia Arechav. var. procumbens Parodi, Revista Fac. Agron. Veterin. (Buenos Aires) 6: 37. 1927. **Type.** ARGENTINA. Buenos Aires, Feb. 1892, Spegazzini s. n. (holotype: MVM, n.v.; isotype: BAA-914!).
Festuca procumbens Muhl., Descr. Gram. 160. 1817. Nom. nud. Diachroa procumbens (Muhl.) Nutt., Trans. Amer. Philos. Soc., ser. 2., 5: 147. 1837. Nom. illeg. Diplachne procumbens (Muhl.) Nash, Man. Fl. N. States 128: 1901. Hom. illeg. pro Diplachne procumbens Arechav., Anales Mus. Nac. Montevideo 1: 414. 1894. Festuca prostrata Muhl. ex Scribn. and Merr., U.S.D.A. Div. Agrost. Circ. 27: 5. 1900. Nom. inval. pro syn. Festuca prostrata Muhl. Type. Habitat in Carolina, Elliott (holotype: P [P00032382], PH [image!] and J. Teisher [PH], pers. comm. (2017)].
Diplachne acuminata Nash in Britton, Man. Fl. N. States and Canada 128. 1901. Leptochloa acuminata (Nash) Mohlenbr., Ill. Fl. Illinois, 293. 1973. Leptochloa fascicularis (Lam.) A. Gray var. acuminata (Nash) Gleason, Phytologia 4(1): 21. 1952. **Type.** United States of America. Kansas. Riley Co., Manhattan, 19 July 1892, CH Thompson s.n. (lectotype, here designated: NY! [NY 00019505]; fragment at US [US-865882]!). Sources indicating the specimen is holotype are incorrect given that no specimen was cited in the protologue; lectotypificatoin is thus needed.
Diplachne maritima E.P. Bicknell, Bull. Torrey Bot. Club 35(4): 195. 1908. Leptochloa fascicularis (Lam.) A. Gray var. maritima (E.P. Bicknell) Gleason, Phytologia 4(1): 21. 1952. **Type.** United States of America. Massachussetts: Nantucket Island, sandy shores of Sachacha Pond, Sep. 16 1899, EP Bicknell s.n. (lectotype, here designated: NY [00079838]!). Lectotypification is necessary because individual specimens were not cited in the protologue.
Tridens veralensis Cat. Guerra, Act. Bot. Cub. 4: 4. 1980. **Type.** CUBA. Prov. de Pinar del Rio: Guanahacabibes, El Veral, Catasus 298 (holotype: HAC, n.v.).

##### Type.

“South America”, *D. Richard s.n.* (holotype: P!; isotypes: US-2875408!, US-3875408a! fragm. ex P).

##### Description.

Annuals. Culms (3.5–)15–130 cm tall, 1–5(–8) mm wide at base, round, ascending to erect (or completely prostrate and densely cespitose at higher elevations; or rarely rhizomatous and rooting at nodes in freshwater marshes, often branching; nodes glabrous; internodes (0.5–) 3–18 cm long, soft, hollow. Leaf sheaths mostly longer than the internodes, round or somewhat flattened, glabrous on sides and margins; ligules (2–)5–7 mm long; blades 3–45 cm ×2.5–7 mm, usually sparsely scabrous above and below. Panicles (1.5–)10–72 ×(1–)4–22 cm, generally partially included below at maturity with 3–35 branches; branches (0.5–)3–22 cm long, alternate along the rachis, erect, ascending, or occasionally divergent, rigid or slightly flexuous, minutely scabrous, the axils glabrous. Spikelets 5–12 mm long, distant to mostly imbricate; florets 6–12; callus glabrous; lower glumes 2–3 mm long, narrowly ovate or somewhat asymmetric, scabrous on midnerve, acute to aristate and occasionally short-awned; upper glumes 2.5–5.0 mm long, elliptic to ovate, scabrous on midnerve, obtuse, aristate, or short-awned; lemmas 2.5–5 mm long, narrowly ovate, light brown or smoky white at maturity, the lateral nerves pronounced and extending to edges often as small teeth, sparsely sericeous along lateral nerves and often midnerve, glabrous between nerves, apex acute to attenuate, often mucronate or with awns to 3.5 long; paleas elliptic, generally subequal to lemma, sericeous along nerves; apex acute to obtuse. Stamens 3; anthers 0.2–0.5 mm long, yellow. Caryopses 1–2 mm long, 0.8–1.0 mm wide, elliptic to obovate in hilar profile, transversely elliptic in transverse section, hilar groove lacking, smooth, brown; pericarp weakly adnate to the endosperm.

**Figure 6:**
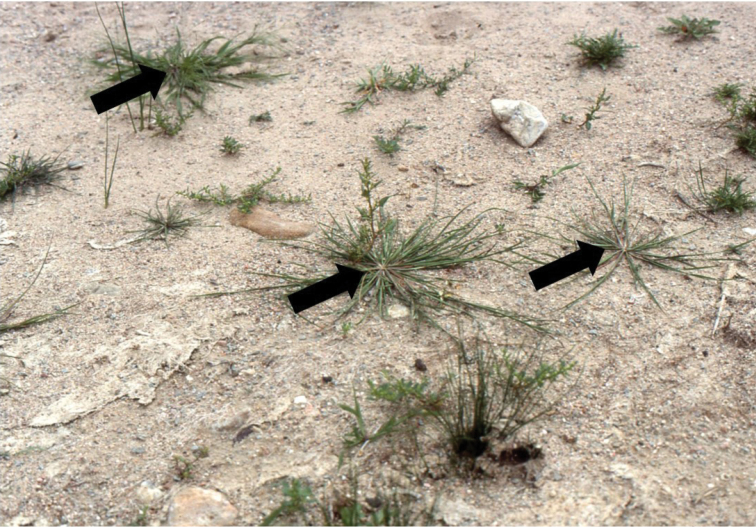
Prostrate growth form (arrows) of a high altitude (ca. 1425 m) population of Diplachne fusca subsp. fascicularis along margins of water impoundment (Platte Co., Wyoming; Snow 6082).

##### Leaf anatomy.

Midrib present; central lucunae present. **Primary bundles**: protruding adaxially or not; protruding abaxially; outer bundle sheath interrupted adaxially and abaxially; extension cells present adaxially; adaxial sclerenchyma present as girders or strands; abaxial sclerenchyma present as girders; adaxial cells of primary bundle sheath cells enlarged; abaxial cells of primary bundle sheath cells not enlarged. Colourless cells present between primary and secondary bundles; chlorenchyma continuous or discontinuous between adjacent bundles. **Secondary bundles**: protruding adaxially or not; flush abaxially; outer bundle sheath continuous or interrupted abaxially; adaxial sclerenchyma present as girders or strands; abaxial sclerenchyma present as girders.

(Vouchers [at MO]: Snow 5804 [USA], 6672 [México].)

##### Stem anatomy.

Internodes hollow. Inner sclerenchymatous ring present. Peripheral sclerenchymatous ring present. Peripheral sclerenchymatous girders connected to the outermost vascular bundles absent. Intervascular peripheral sclerenchymatous pillars not associated with outermost vascular bundles absent. Inner sclerenchymatous ring canal tissue present. Kranz sheath cells absent. Kranz sheath cell canal tissue absent. Vascular bundles nested in outer portion of Kranz sheath cell canals absent. Sclerenchymatous rings surrounding vascular bundles located inside inner sclerenchymatous ring absent. Sclerenchymatous rings (5–10 cells thick) surrounding outermost primary vascular bundles absent. Phloem not divided.

##### Chromosome number.

2*n*=20 (Gould 1966; Reeder 1971; vouchers: Reeder & Reeder 4844 & 4883 (ARIZ!)).

##### Phenology.

Flowering mostly May to October in North America and throughout the year in South America during and after warm and wet seasons.

##### Distribution.

Southern Canada (infrequently) through USA (where most common) south sporadically to Paraguay. The transition between the native and non-native distribution of L. fusca subsp. fascicularis is somewhat uncertain, particularly in its sourtherly range. Elevation from sea level to 2300 metres. **Native**: Most of the United States mainland (excluding Georgia) north to Ontario, Canada, occurring irregularly to about 40°S in Argentina. (TDWG: in North America in part or all of regions 73-80 (see additional specimens for additional detail): BLZ, COS, HON; 81: BAH, CAY, CUB, DOM, HAI, JAM, LEE-NL, LEE-VI, TCI; 82: VEN; 83: BOL, COL; 84: BZC, BZE, BZL, BZS; 85: AGE, AGW, AGW, PAR. **Non-native**: TDWG: CZE. Collected once from a railyard in Vancouver, British Columbia but not persisting (F. Lomer, pers. comm. 2010). Possible occurrences in Saskatchewan and Quebec have not been confirmed.

##### Conservation status.

Least Concern (IUCN 2012), given its wide distribution and abundance.

##### Etymology.

Possibly in reference to the fasciculate (bundled or clustered) arrangement of the lower panicle branches before they are exserted from the sheath.

##### Vernacular names.

bearded diplachne; salt meadow grass; zacate salado lagunero; salado fasciculado.

##### Comments.

Specimens from Florida and the Bahamas typically have the shortest spikelets (e.g. Vincent 16032 [MU]; Davis s.n. [FLAS 38436]; Craighead s.n. [FLAS 84837]; Herndon 905 [RSA]). On rare occasions, a specimen growing in a saturated soil may branch and root so vigorously at nodes that it appears to be stoloniferous (e.g. Fleetwood 12215 [SMU]). Older specimens from Reno, Nevada, identified as *Diplachne tracyi* Vasey, have narrow, relatively long and somewhat cylindrical spikelets (e.g. the type specimen), but since this spikelet morphology recurs in the species complex from parts of Africa and Australia and is narrowly distributed, this variant is unworthy of taxonomic recognition. Those specimens may be waifs of D. f. subsp. fusca from the Paleotropics, but genetic analyses would be necessary to test this hypothesis. Some specimens from California (Twissleman 6491 [MO]) also closely resemble some forms of D. fusca subsp. fusca.

A morphotype in the United States described as *Diplachne acuminata* Nash in Britton has been recognised based on the relative elongation of glumes and lemmas and awned lemmas (Gleason, 1968: 188; Gleason and Cronquist, 1991: 785). *Diplachne maritima* E.P. Bicknell was described based on its prostrate habit, more pronounced lemmatal awns and ecological occurrence in brackish coastal areas from Massachussetts to Florida (e.g. Weatherby 4390; Commons 155; Metcalf 5717; Torrey 8765; Williams 3102 [all at US]) and around saline areas in Onondaga and Cayuga counties, New York (e.g. Leonard 8559 [FLAS]; Congdon s.n. [RSA]; Perkins s.n. [RSA], Fogg 3889 [MO]). However, given that three of the four subspecies of *Diplachne fusca*, recognised in this treatment, have moderate to high levels of salinity tolerance (but little evidence for this with subsp. *uninervia*) and that populations with elongated awns often occur elsewhere, such as in the Distrito Federal and Estado de México (Jiménez–Osorino 40 [RSA]; Mattuda 25656 [MEXU, MO, MU]; Espinosa & Sarukhán 318 [ENCB, MEXU, MO]; Rzedowski 26251 [ENCB, MO]; Rzedowski 28145 (ENCB, US); Villegas 585 [ENCB]; Rzedowski 20432 [ENCB], Schaffner 49 [W]), taxonomic recognition of specimens with longer than average lemmatal awns also is unwarranted.

Specimens have not been confirmed for this subspecies listed on websites from Sweden (e.g. Lackalänga, year 1949 [LD, S]) and Poland (Vogel, years 1916 and 1918 [OHN]).

Diplachne fusca subsp. facicularis is more widespread in North America than subsp. uninervia, although the latter is more invasive outside of its range (e.g. Snow & Simon 1999). Specimens morphologically intermediate beween *D. f.* subspp. *fascicularis* and *uninervia* occasionally are seen (Hitchcock 93 [TEX]), but the taxa maintain their distinct morphology in sympatry (e.g. Snow 5899 and 5900 at Falcon Lake, Zapata County, Texas).

Sutton (1973a) briefly described the anatomy of *D. fascicularis* (as “*fascicularias*”) and placed *Leptochloa* into the tribe Chlorideae (Sutton 1973b), but these papers extrapolated the limited analysis beyond their usefulness. Clifford and Watson (1977) illustrated D. fusca subsp. fusca (as *Diplachne parviflora*). Renvoize (1984) illustrated the midrib of *D. fusca* and discussed its prominent air canal, but cited no voucher.

Characters of Diplachne fusca subsp. fascicularis that generally differ from those of subsp. uninervia include: lower panicle branches inserted in sheath, a greater tendency for sheaths to be mottled with anthocyanins (e.g. McGregor 12652 [KANU, US]), the tip of the uppermost leaf blade often extending beyond the apex of the panicle and the lemmatal apices generally acute to acuminate and sometimes mucronate on the lateral nerves. Specimens of *D. f. subsp. fascicularis* with exserted panicles bearing relatively short, somewhat flexuous branches (Morello and Cuezzo 1124 [BAA])) can resemble *Dinebra scabra* (Nees) P.M. Peterson & N. Snow. Relatively erect specimens of *Dinebra viscida* (Scribn.) P.M. Peterson & N. Snow are often confused for *D. f. subsp. fascicularis* where the species overlap, but the panicle length and width of the former are usually significantly shorter and the fresh foliage is slightly sticky or viscid.

##### Specimens examined.

**Argentina**. Buenos Aires: Ptdo. de San Fernando, cerca dela estación “El Delta”, Hunziker 3561 (MO); Avellandeda, Venturi 209 (GH, NY, US); Villa Elina, Cabrera 6321 (LP). Catamarca: Dpto. Capayán, Ruta 60 (Km 1044) entre San Martin y El Medano, Hunziker 21023 (NY); Dpto. Santa María, Entre Ríos, Reales 1596 (BRIT). Córdoba: Entre San José de la Esquina y San Ricardo, Birabén 48 (LP). Salta: Cerca de Dragones, Ruta 81, Cabrera et al. 26544 (LP); Quebrada de las Conchas, Senn 4143 (MO). San Luís: Dpto. Junín, Bajo de Velis, Kurtz 8506 (LP, W). **Bahamas**. Acklin: Along road south of Lovely Bay, Correll & Proctor 48872 (NY, US). Great Inagua: Open gravel area by the “town pans”, Dunbar 223 (A, US); In mud of Grassy Pond, E of Morton Bahamas Ltd. headquarters, Correll 47401 (NY); In mud of sink at Horse Pond, just NE of Matthew Town, Correll 45812 (NY); In clumps in water of pond, Horse Pond, NE of Matthew Town, Correll 47549 (MO); Cleared portion of Maroon Hill, NE of Matthew Town, Gillis 12131 (A); Inaqua, water hole near airport runway, Gillis 11750 (A, MO); dry pond in algal mat, 2.5 mi.N of Mathew Town on road to salt works, Gillis & Proctor 11727 (A). Andros Island: North Andros, along Queens Hwy at Stafford Ck, at roadside, Jacobs s.n. (MU); The Bluff, Freid 06-713 (MU). Long Cay: South Side, Brace 4084 (NY). Long Island: A short distance NE of St. Paul’s Church at the base of the slope, Clarence Town, Hill 2339 (MO); Edge of palmetto flat near Alligator Bay, Correll 48200 (NY); Millerton, Freid and Richley 98-423 (MU); Stella Maris, Fried and Richey 98-341 (MU). Grand Bahama: In ditch along road to dump, W of Freeport Airport, Correll & Correll 50942 (NY); Open moist area of Bucida spinosa marsh, W end of Freeport Airport, Correll and Kral 43008 (NY). New Providence Island: Near Nassau, Curtiss 176 (CM, NY, P, PR); Edge of freshwater marsh, SW Bay, Britton and Brace 504 (US). Great Exuma: Sink hole near Georgetown, Britton and Millspaugh 3108 (GH, NY, US). Cat Island: Orange Ck. and vicinity, Britton and Millspaugh 5719 (NY). **Bolivia**. Cochabamba: Prov. Mizque, Town of Mizque, wetland adjacent to the Río Mizque, near the intersection with the road to Aiquille, Ritter et al., 2119 (NHA); Prov. Cercado, City of Cochabamba, Laguna Alalay, Ritter 1674 (NHA). **Bonaire** (Nether Antilles). Back of the dam near Jatoe Bacoe, Stoffers 662 (A). **Brazil**. Bahia: Cachoiera, base of cliff in slimey water, Chase 8097 (US); Rio de Contas a Jequié, Davidse et al. 11647 (K, MO, TAES); Iacú, Faz. Suíbra (Boa Sorte), 18 km al este da cidade, Noblick 3655 (K); Mpio. Livramento do Brumado, agua Vargem de Dentro, c. 8 km ao oeste da cidade, Harley et al. 25858 (K); Rio Salitre, 46 km WSW of Joaziero, Chase 7934 (BAA, GH, MO, US, W); 64 km N of Senhor do Bonfim on the BA 130 Hwy to Juaziero, Harley et al. 16343 (K, NY, P, US). Ceará: Thickets on shores of Acude Choró, Mpio. de Quixadá, Drouet 2408 (US); Picos, Piauhy to Campo Salles, Swallen 4265 (BAA, US); Campo Salles to Crato, Swallen 4300 (US); Iguatú, Swallen 4415 (US); Cratheus, edge of small pond, Swallen 4497 (US). Distrito Federal: Brasilia, Luetzelburg 418a (K) and Luetzelburg 794 (K). Paraíba: Escola de Agronomia do Nordeste, Areia, Paraiba, Coêlho 1145 (US); Pocinhos, Pickel 3841 (MU). Pernambuco: In low field, Tapera, Pickel 1701 (GH, MICH, P, US); Caruaru, on border of a rivulet, Pickel 4257 (MU); Bello Jadim, Serra do Genipapo, Chase 7685 (GH, MO, US); Vicinity of Pernambuco (Recife), Chase 7740 (MO, US); Mpio. Belem de São Francisco, 3 km S of city of Belem de S. Francisco (also called Jatanã) at point where road stops at shore of Rio São Fransisco, Eiten & Eiten 4958 (K, US). Rio de Janeiro: Margin of Lagoa R. de Freitas, vicinity of Rio de Janeiro: Chase 8457 (GH, MO, US); Avenida Niemeyer, Chase 9991 (US). Rio Grande do Norte: Angicos, Swallen 4704 (GH, MICH, US); Nova Cruz to Montahnas, Swallen 4821 (US). Rio Grande do Sul: Estação Experimental de Uruguaiana, Simas 6590 (TAES); Santa Cruz, Swallen 4857 (US). **Caicos Islands**. Pine Cay: In moist area on NW side, Correll 43169 (NY). **Canada**. Ontario: Junction of Hwy 3 and Regional Rt. 14, SW of Canborough, Regional Mpio. of Haldimand–Norfolk, Cusick 29191 (MICH). Elgin Co., St. Thomas railway yard, Oldham, 9865 (MICH); 4.7 km WNW of Dutton, Hwy 401, service centre, Oldham 8828 (MICH); Yarmouth Township, Port Stanley, beach ca. 0.5 km SSE of Post Office, W of Orchard Beach, Oldham 11897 (MICH). Essex Co., Anderdon Tp, just S of Canard Riv. mouth, W side of Hwy 18, at Ranta Marina, Oldham 6953 (MICH). Halton Co., City of Burlington, Burlington West Railway Station, Oldham 13523 (MICH). Huron Co., Hay Township, CN railway tracks at Hensall, Oldham 13567 (MICH). Lambton Co., City of Sarnia, railway yard in Sarnia, Oldham 13075 (MICH). Middlesex Co., Hwy 401, from ca. 0.5 to ca. 2.0 km W of Dorchester Rd., Oldham & Deslisle–Oldham 7033 (MICH). Oxford Co., Norwich Township, 5.4 km E of Woodstock Post Office, Hwy 401 at Hwy 403 junction, Oldham 11816 (MICH). **Cayman Islands**. Grand Cayman: Bodden Town, Guala and Burton 1934 (US). **Colombia**. La Guajira: Costa del Caribe, llanura litoral arenosa y árida entre Mayapo y El Pájaro, Dugand 6667 (COL, US). **Cuba**. Havana: Batabano, Shafer 484 (CM, NY); Batabano, Baker 2762 (POM); Batabano, Ekman 889 (US); Batabano, Baker 1767 (NY). Las Villas: Cayo La Lisa, Río Negro, Zapata Swamp, León et al. 19565 (GH); La Lisa, Rio Negro, Ciénega de Zapata, León 19565 (US). Matanzas: Cardenas, Britton and Wilson 176 (NY). Oriente: Los Caños, S of Guantánamo, León 3911 (US); La Perla, N of Guantánamo, León 3911 (GH). Pinar del Rio: Corrientes Bay, Britton & Cowell 9984 (NY, US); Central Orozco, in dried up pools near the manglares, Ekman H10570 (US). Unknown province: Playa de Marianao, León 2020 (US); Santo Cristo de Maniadero, Peninsula de Zapata, Roig & Cremata 2225 (NY); Coastal Marsh (Playa) SE of Baraguá, Hitchcock 23337 (US); Isle of Pines, Killip 41664 (GH). **Czech Republic.** Distr. Děčín: ship to railway transfer area Nové Loubí in Děčín, V. Jehlík s.n. (PRA). Distr. Ústí nad Labem: Western Port on Labe River in Ústí nad Labem, V. Jehlík s.n. (PRA); Railway station Ústí nad Labem sever, V. Jehlík s.n. (PRA). **Dominican Republic**. Independencia: Barahona, vicinity of Cabral, Jiménez & Marcano 4290 (TAES, US). Valverde: Esperanza, Jiménez 8169 (NY, TAES). **Haiti**. Nord: Plaine du Nord, Cap Haitien, edge of salt water ditches, Ekman H2750 (A, US). Ouest: Vicinity of Etang, Etang Suamatre, Leonard 3520 (GH, NY, US); Massif de la Hatte, Miragoane, shore of Etang–Miragoane, Ekman H7257 (US); Port–au–Prince, Bon–Répos, in irrigation ditches, Ekman 2105 (US); Port–au–Prince, Potter 5012 (GH); Massif de la Selle, Croix–des–Bouquets, edge of Grande–Rivière, near Gourjon, Ekman H6453 (US). **Honduras**. Choluteca: 30 km carretera de Choluteca a Cedeño, Midence 32 (TEFH). **Jamaica**. Saint Ann Parish: Saint Ann’s Bay and Vicinity, Marsh, Flat Point, Britton 2519 (NY, US). Saint Elizabeth Parish: Salt Spring to Borad Riv., Adams 12058 (MO); Black Riv. and vicinity, roadside ditch, Britton 1339 (NY); Black Riv., Hitchcock 9643 (US). Westmoreland Parish: Savanna-la-Mar, swamps and dithes, Hitchcock 9863 (US); Ferry Peux, Harris 12498 (CM, GH, MO, NY, P, US, W). **México**. Aguascalientes: Rincon de Romos, Hernández & Mathus N–1609 (GH); 16 km al N de Aguascalientes, sobre la carretera a Rincón de Romos, Rzedowski & McVaugh 731 (ENCB, NY). Campeche: Carr. Tankuche–El Remate, 1 km al S del camino, Mpio. Calkini, Ortíz 761 (UC). Chihuahua: Km 33 Nuevo Casas Grandes–Galeana, Hernández and Tapia N–130 (US); Hilly terrain, 9 mi.S of Villa Matamoros, Reeder & Reeder 4883 (ENCB); Rio Conchos, Cd. Camargo, Harvey 1403 (GH, MICH, US); Rio Aros, LeSueur 202 (GH, SMU, US); Santa Maria en el Valle de Aldama, Hernández and Mathus N–1744 (GH); 28 km SW of La Junta on road to Creel, Peterson 9610 (BISH, K, US); Low area between Hwy and railroad tracks... 11 mi. W of Cuauhtémoc, Reeder et al. 4844 (ARIZ, ENCB, US); Torreon, Johnston 44166 (ARIZ, MO, SMU); Plains near Chihuahua, Pringle 813 (GH, MEXU, MICH, MO, NA, NY, P, PR, RSA, S, UC, US, W); ca. 65 (air) mi. SW of Cd. Juarez, 6.8 road mi. N of Guzman, Henrickson 14105 (LL); At Paplote Las Juntas (Preson de Anteojos), 2 km NW of Hacienda El Berrendo, on Las Pampas Ranch, Chiang et al. 8870 (LL); Rio Florido, Jiménez, Harvey 1320 (ENCB, TEX, US); At Papalote Las Juntas, (Presón de Anteojos), 2 km NW of Hacienda El Berrendo, on Las Pampas Ranch, Chiang et al. 8870 (MO). Coahuila: Don Martín Dam, Harvey 944 (MICH, US); ca. 51.5 (air) mi. ENE of Torreon on SW edge of Laguna Mayran, along trail from Estacion Cuchilla to Ejido Mayran, Henrickson 14349 (LL); 20 mi.NW of San Pedro ... 63 km de Ocampo rumbo a Sierra Mojada, Carranza C–631 (ARIZ, MO, TEX); Sierra La Madera, Rcho. Laguna la Leche, aprox. 62 km de Ocampo rumbo a Sierra Mojada, Carranza 617 & Carranza (BRIT); road to San Martín Dam near near Nuevo Leon–Coahuila border, Harvey 947 (GH, MICH, MO, MONTU, US); 10 km carretera Matamoros – Saltillo, Espinosa A. 89 (ARIZ); Hwy 30, in wet shore of Laguna de Mayran, Henrickson 5985 (LL); Torreón, Palmer 503 (GH, MO, TAES); Torreón, Fisher 44166 (NY, TAES). Distrito Federal: Tlahuac Distr., chinampas at San Andrés Mixquic, 40 km SE of Mexico City & 7.5 km W of Chalco in the Chalco Lk basin, Jiménez-Osornio 40 (ARIZ); Ixtapalapa, en ladera humeda cerca de canal, Matuda 25656 (MEXU, MO, MU); Xochimilco, Espinosa & Sarukhán 318 (ENCB, MEXU, MO); NW de Xico Viejo, cerca de la aldea, Villegas 558 (ENCB); Deleg. de Tláhuac, Míxquic, orilla de canal, Rzedowski 26268 (ENCB); W de Tláhuac, en zona de dhinampas, Villegas 564 (ENCB); Xochimilco, Rzedowski 20432 (ENCB, MICH); Tláhuac District: Chenampas at San Andrés Mixquia, 40 km SE of Mexico City and 7.5 km W of Chalco in the Chalco Lake Basin, Jiménez–Osorino 40 (RSA). Durango: Durango, Hitchcock 7567 (US); Torreón, Hitchcock 7729 (US); Cienega bottomland near small lake 40 mi.N of Ciudad Durango, Gentry 8600 (ARIZ, GH, MICH, RSA, US); 10 mi. NE of Durango on Durango–Torreén Rd., Soderstrom 803 (SMU, US); 66 km N of Durango on HWY 45, Peterson & Annable 5987 (BISH, US); Colonia Filipe Angeles, at 15 km S of Durango on Hwy 23 towards Mezquital, Peterson et al. 18963 (BISH, US). Guanajuato: Irapuato, Hitchcock 7386 (US); ca. 6 km W of San Felipe, Sohns 402 (MICH, MO, NY, TAES, US); 6 km al NNE de Salvatierra, sobre la carretera a Celaya, Rzedowski 40295 (ENCB). Guerrero: Near Pie de la Cuesta, 6 mi. N of Acapulco, Barkley et al. (TEX). Hidalgo: Mpio. Huichapán, Roadside ditches near El Carmen on Huichapan–Querétaro Rd., Moore 2158 (GH, UC, US); El Peñón, 5 km al sur de Alfajayucan, Mpio. de Alfajayucan, Hernández 6721 (MO). Jalisco: Villa Obregon, Beetle M-5545 & Guzmán (ARIZ); 2 km from San Julian, Beetle M-5583 & Guzmán (ARIZ); Hwy 54, along Río Juchipila, ca. 50 mi. N of Guadalajara, just past Puente Santa Rosa, Spellenberg 2945 (NMC, NY); 3 km N Ojuelos, Hernandez X-2487 (KANU); 2 km al E de San Diego de Alejandría, rumbo a la Cd. de León, Lot & Novel 969 (GH); Mpio de Ixtlahuacán del Rio, Atotonilquillo, Guzmán 8377 (ENCB); Isla de Alacranes, Lago de Chapala, Cota 56 (ENCB); El Jaguey, 14 kms al N de Ixtlahuacán del Rio, Guzmán 7977 (ENCB); 23.3 km E of Aguacalientes on Hwy 70 towards San Luis Potosi, just past state boundary, Peterson & Annable 6186 (US); ca. 6 mi. W of Lagos, Reeder et al. 1416 (GH, RSA); Laguna de Zacoalco, Diaz Luna 1063 (ENCB); ca. 50 mi. N of Hwy 54 along Río Juchipila, just pass Puente Santa Rosa, Spellenberg et al. 5342 (NMC). México: S de Ixtapaluca, a 2 km del pueblo, Villegas 585 (ENCB); 1 km al N de Zumpango, Rzedowski 25850 (ENCB, MICH); Lago de Texcoco, 18 km al WSW de Texcoco, sobre el camino a México, Rzedowski 28145 (ARIZ, ENCB, MICH, US); Atenco, cerca de Texcoco, Torres s.n. (ENCB). Michoacán: 6 km al E de Maravatío, sobre la carretera a Contepec, Rzedowski 44199 (ENCB); Shore of Lake Pátzcuaro, Barkley et al. 2669 (US); Shore of Lake Pátzcuaro, Barkley et al., 2663 (TEX); Vicinity of Morelia, Arsène 5760 (CM, GH, MO, S). Morelos: Morelos. Valley near Jojutla, Pringle 9595 (GH, MEXU, MO, NY, TAES, US). Nuevo Leon: 32 mi. S of San Roberto along Hwy 57, McGregor et al. 501 (KANU); Monterrey, Arsène 6108 (NY, US); Monterrey (Nuevo Leon) río, Abbon 201 (MO); Pan Am. Hwy from Laredo to Monterrey near Maulique Pass, Leavenworth & Leavenworth 740 (MO, US); Mpio. de Linares, Ejido Los Cristales, Ortíz s.n. (ENCB). Querétaro: Cerro de las Campanas, Arsène 10072 (GH, MO); Querétaro, in water of irrigation ditch, Hitchcock 5836 (US); Querétaro, Arsène 10289 (US); 2 mi. SE de San Juan del Rio, O’Bien s.n. (MEXU 321518). Quintana Roo: Cozumel al sur de la Islan a 200 m del embarcadero, Ortíz 904 (MEXU, MO); Punta Nizuc, Can–Cun, Mpio. Isla Mujeres, Ortíz 530 (MEXU). San Luís Potosí: Charcas, Lundell 5535 (ARIZ, MICH, US); 8 km al E de S. L. Potosí; Rzedowski 11236 (ENCB); Mpio. Guadalcazar, Ejido Minas de Plata, Bravo 34 (ENCB); ca. 2 mi. W of Arriaga on road from Aguascalientes to S. L. Potosí, Reeder et al. 1358 (ARIZ, ENCB, MEXU); Charcas, Whiting 1052 (ARIZ, MEXU, MICH, TEX, US); Mpio. Charcas, Laguna Seca, Rzedowski 6535 (ENCB, LL); 5 mi. SE of SLP, Gould 11566 (ENCB, TAES, UC, US); Rio Verde, along polluted creek on N side of town (along road to Las Tables), Thomas 2773 (MO). Sinaloa: Along Hwy 15 (de cuota), at marker 19 km N of Mazatlán, Snow 6599 (MEXU, MO); Mazatlán, Harvey 8828 (MONTU). Sonora: Mpio. Agua Prieta, W Side of Agua Prieta, Reina G. 2006-445 (ARIZ); Mpio. Empalme, on Mex. Hwy 15, 10.3 mi. E of Empalme end of Douglas Bridge, Felger 85-1141 (ARIZ); Mpio. Guaymas, Cañón La Balandrona, north side of Sierra El Aguaje, Felger 01-655 et al. (ARIZ); Mpio. Guaymas, ca. 6 km E of Las Guasimas exit, ca. 31 km ESE of Empalme on MEX 15, Van Devender 2002-1072 et al. (ARIZ); Mpio. Guaymas, Mex. Hwy 15, 1.7 km NW of Pitahaya (Belem, Rio Yaqui) junction, 3.6 mi. S of Pitahaya, Felger 85-1293a & Reichenbacher (ARIZ); Mpio. Guaymas, Cañón Las Barajitas, Felger 96-51 et al. (ARIZ); Mpio. Hermosillo, Hwy 24, 5.0 mi. N of El Sahuaral (4.7 mi. N of Bahia San Augustin Road junction), Felger 85-1570 (ARIZ); Mpio. Cucurpe, Cañon las Barajitas, Sierra el Aguaje, ca. 18 km NW of San Carlos, Felger 95-228 & Wilson (ARIZ); Reservoir, ca. 6 km SE of Magdalena off the road to Cucurpe, Reina G. 2001-750 & Van Devender (ARIZ); Rio Mayo drainage, along the Hwy from Navojoa to Huatabampo, ca. 0.6 mi. NE of turnoff to Bacobampo and 8 mi.SW of Navajoa, Sanders et al. 8945 (ARIZ) and 8947 (MO, UC); Mayocahui, Van Devender 95-292 et al. (ARIZ); On paved road leading to Ortíz, 14.1 road mi. N of intersection with Hwy 15, SE of Guaymas, Snow 6564 & Prinzie (MEXU, MO); SE of Guaymas, along Hwy 15, ca. 100 m S of km 82 signpost, Snow 6566 & Prinzie (KSP, MEXU, MO, MU); Between road and railroad tracks, at km 90 on Hwy 15, SE of Guaymas heading to Ciudad Obregon, Gibson & Gibson 2043 (ARIZ, ENCB). Tamaulipas: Sierra de San Carlos, Vicinity of El Mulato, Bartlett 10972 (GH, MICH, US); Mpio. Aldama: Ejido La Piedra, Mora-Olivo 1478 (BRIT). Tlaxcala: Entrada W de la población de san Jorge Tezoquipan, al W de Tlaxcala, Weber 697 (ENCB). Yucatán: Progreso, Swallen 2918 (MICH); Carr. San Felipe–Rio Lagartos, a 5 km del crucero, Mpio. Rio Lagartos, Ortíz 691 (UC); Mpio. Sisal, 4 km al SE de Sisal, Santos Meza 60 (MO); Mpio. Progresso, mangrove 3 km S of El Progresso, Davidse & Davidse 29454 (MO); 18 km al SE de Celestún, Gutiérrez–Parra 30 (ENCB). Zacatecas: Along Hwy 54, at turnoff to Jalpa, Snow 6672 (MEXU, MO); Villa de Cos, Johnston 7429 (GH, US). **Paraguay**. Boquerón: Filadelfia, Hahn 801 (MO); A 12 km E de Pratts Gill, Molas & Vera 1388 (MO). **Turks and Caicos**. Pine Cay, Correll 43169 (LL); Provo, Long Bay, Neis 406 (MU). **United States of America**. Alabama: Mobile Co., Battleship Park, by causeway US 90–98, E of Mobile on the bay, Kral 32754 (US); Along (and S) of the newly completed Interstate Hwy 10 overpass, SE part of Blakeley Island between Mobile Riv. and Mobile Bay, Lelong 7323 (NCU). Morgan Co., Port DeKatur, sandy beach by Dekatur boat ramps along Tennessee Riv., Kral 54428 (MO). Arizona: Apache Co., Canyon de Chelly Nat. Mon., Capmground, Rink 1555 (ARIZ). Cochise Co., Mule Mountains, near watercourse, Goodding 297-61 (ARIZ); Douglas, Tornber s.n. (ARIZ 147338); S edge of St. David, Gould & Haskell 4496 (ARIZ, GH); playa just W of Willcox, Reeder & Reeder 7955 (ARIZ); just W of Willcox along road to Cascabel, Reeder & Reeder 8879 (ARIZ); Willcox Playa area, SW of the town, Reeder & Reeder 9061 (ARIZ); San Pedro Riparian National Conservation Area, St. David Cienega, just W of Upper San Pedro River, N end of wetland, Makings 1624 (ASU). Gila Co., Black Riv., near settlement of Black Riv., Gould & Robinson 4925 (ARIZ, UC). Graham Co., Hooker Cienega, SW of Bonita along Sunset School Road, Jenkins 2466 (ARIZ); Tatcher, Thornber s.n. (ARIZ 147336); San Simon Valley, 15 mi.NE of Bowie, Bingham 2338 (ASU). Mohave Co., Arizona Strip, Rock Crossing along the Colorado City-Main Street Valley road, Reeder & Reeder 9649 (ARIZ); Around a large charco at Rock Crossing on the Colorado City to Main Street Valley road, Reeder & Reeder 8627 (ARIZ); ca. 5 km S of jct of AZ-389 and Cane Beds road, then ca. 10.5 km west, Reeder & Reeder 8441 (ARIZ); Havasu Nat. Wildlife Refuge, Krakowski s.n. (ASU 70812). Navajo Co., Woodruff, in moist soil along reservoir, Darrow 3324 (ARIZ, US); Hwy 260, ¾ mi. SE. Ent. St., Stephenson 1968 (ARIZ). Pima Co., edge of old clay quarry, flats W of Tumamoc Hill, Tucson, Bowers 2716 (ARIZ). Pinal Co., 9 mi. N of Casa Grande overpass on Hwy 87, Pultz & Phillips 1532 (NY); Southwestern shoreline of Picacho Lake, Gould 4630 (ARIZ); Near the Mormon Batallion Monument along AZ-387 ca. 5 km N of its jct. with AZ-84 in Casa Grande, Reeder & Reeder 9599 (ARIZ). Santa Cruz Co., Along road to Canelo Pass, ca. 5 km S of its jct with AZ-83, Reeder & Reeder 8924 (ARIZ); 1 km N of Canelo Pass summit, Reeder & Reeder 9896 (ARIZ); N of Canelo Pass summit, Reeder & Reeder 9899 (ARIZ). Yavapai Co., Perkinsville Quadrangle, T17N R2E SE ¼ SE 1/4 , NW of Jerome, W of Mormon Pocket, east of Horsesehoe Canyon, along Verde Riv., Baker 12076 & Routson (ARIZ, ASU); Chino Valley North, Inscription Ranch, along the Verde River 2.7 km ENE of its confluence with Granite Creek, Baker 12753 (ASU). Yuma Co., Backwater A-7 to Colorado Riv., 1 mi. S of Ehrenberg, on east shore ca. 1 mi.S of N end of lake, Kennedy 10 (ARIZ). Arkansas: Arkansas Co., US Hwy 79, 0.3 mi. NE of junction with US Hwy 165, southbound just N of Stuttgart, Snow 5809 (MO); US Hwy 79, 0.2 mi. NE of junction with US Hwy 165 (southbound), just N of Stuttgart, Snow 5811a (MO). Drew Co., Along railroad tracks at Ark. Hwy 138 and Coon Bayou in Winchester, Thomas et al. 171074 (GREE); Lewisville, Sundel et al. 11976 (KANU). Lawrence Co., 4 mi.SW of Hoxie, Taylor s.n. (MU barcode 00008103). Ouachita Co., RR tracks at the Port of Camden E of Adams St., in downtown Camden, Thomas 171837 (BRIT, MU). Pulaski Co., Arkansas Riv. up the river from North Little Rock, Demaree 8364 (SMU, US). Sebastian Co., Arkansas Riv. floodplain, Sec 25, T8N, R31W, Thompson et al. C1074 (GH). California: Butte Co., Lake Oroville at Vinton Gulch, ca. 0.7 (air) mi. N of Cherokee, Oswald and Ahart 4413 (UC); Gray Lodge Game Refuge, NW of Pennigton, Mason 12730 (POM); Joie Osgood Ranch, ca. 2 mi. E of Honcut, Ahart 6721 (MO, UC); ca. 1.1– mi.S of the clay burrow for Oroville Dam, 1/4 mi. E of Larkin Rd., ca. 6 mi.SW of Oroville, Ahart 5480 (MO); Rice fields between Nelson and Richvale, Heller 13354 (GH, MO, NY). Colusa Co., Pond on Colusa–Marysville Hwy, 4 mi. S of Colusa, W side of Sacramento Riv., Mason & Grant 12962 (FSU). Fresno Co., Cottonfield 2 mi. NE of Fresno, Wiggins 4198 (POM); Fresno, Griffiths 4729 (US); Fresno, Springer 469 (ARIZ). Glen Co., S of Willows Migratory Wildfowl Refuge, Beetle 3062 (US). Inyo Co. 4 mi. N of Lone Pine, Kerr s.n. (RSA). Kern Co., Kern Riv. Canyon, Howell 38682 (MO); South Fork Valley ca. 2 mi. NE of Weldon, Howell 40133 (MO); Bed of Lake Isabella below mouth of Tillie Ck., Twisselmann 19263 (MO); Dessicated strand–bed of Lake Isabella (E side of N arm), ca. 5 mi.S of Kernville, Howell 47465 (RSA). Los Angeles Co., Coast Hwy just N of mouth of Santa Monica Canyon, Raven 16753 (RSA); California–Mojave Desert, W end of Rosamond Dry Lake at Ave. “C”, Sanders 2340 (CM); Rancho Santa Ana Bot. Garden, Claremont, Balls 9465 (W). Marin Co., Nicasio Reservoir ca. 10 air mi. SW of Petaluma and W of Novato, Ertter 8018 & Neese (ARIZ). Merced Co., San Joaquin Riv. 1/4 mi. S of Hwy 152 upstream from bridge 2.9 mi. E of Santa Rita Park, Wiggins and Wiggins 20702 (MU, RSA); Los Banos Water Flow Refuge, 3 mi. N of Los Banos, Breedlove 5402 (SMU). San Diego Co., Lake Henshaw, SE of East Grade Road and E of Henshaw Truck Farm, ca. 1.3 air mi. E of the dam, along the stream feeding into the NE portion of the Lake and on the NE shore, Rebman 13640 et al. (ARIZ). San Francisco Co., Street weed in San Francisco, Howell 11697 (US). San Luis Obispo Co., 0.5 km N of Bee Rock Rd on E Perimeter Road, Camp Roberts, Johnson & McCarty ROB0762 (OKL). Santa Barbara Co., San Jose Ck. at Southern Pacific RR corssing, Goleta, Pollard s.n. (ARIZ, RSA). Sonoma Co., 5 mi.N of Sears Point, Rubtzoff 6284 (RSA). Stanislaus Co., Near Oakdale, Hoover 700 (GH, MO). Sutter Co., E side of West Butte Rd., ca. 0.5 mi. S of North Butte Rd., Ahart 6666 (UC). Tulare Co., Only seen near NW corner of vernal pool natural area in valley grassland, 4.5 mi. ENE of Pixley, Howell 44030 (RSA); Along Dinuba Blvd., 8 mi.N of Visalia, Bacigalupi 2509 (GH, MONTU); 1.5 mi. ESE of Stoil, Bradshaw 344 (KANU). Yolo Co., At junction of State Hwy 128 and County Rd. 86 (Pleasants Valley Rd.), Willoughby 1800 (JEPS). Colorado: Alamosa Co., Alamosa, Ramaley 15845 (MICH). Arapahoe Co., Cherry Ck. Reservoir, along southern shorline, Snow 6121 (KSP, MO). Baca Co., SE corner of the county where CR 48 and CR 46 converge just N of the Cimarron River, Clark 2311 (COLO, KANU). Bent Co., John Martin Reservoir, on Arkansas Riv., Lindstrom 1664 (KSC). Delta Co., Between Delta and Austin, Goodding & Goodding 244 (US). Cheyenne Co., 5-1/2 mi. SE of Wild Horse, Harris 70 (RM); Kit Carson, municipal park on N side of town, T15S, R48W, Sec. 4, SW ¼, Freeman 15516 & Morse (KANU). Fremont Co., Canyon City, Clements 263 (MO, NY); Canyon City, Shear 964 (GH). Kiowa Co., Eads, 10 mi. S, 4.5 mi. E [of] Neenoshe Reservoir, Freeman 15998 & Morse (GREE, KANU). Kit Carson Co., Burlington, S side of town ca.1 block N of juct US 385 and I-70, Freeman and Morse 15777 (GREE, KANU). Las Animas Co., Along Carrizo Ck., 6 mi. SE of Troy, Rogers 4910 (MICH, US); Ranchland ca. 4 mi. E of Wlat’s Corner and N on 143.0 Road before the road that descends along the Purgatoire River Canyon, Clark 2528 & Deihl (KANU). Lincoln Co., Limon, E side of town, Kissel Fishing Pond along US 24, Freeman 16528 & Morse (KANU). Logan Co., E edge of Sterling, Taylor & Taylor 8982 (BRIT). Mesa Co., D Rd. near 32nd, Clifton, wet ground around lagoon margin, Weber 15508 (US); Colorado Nat. Monument, sewage ponds, NW of headquaters of Mesatop, Siplivinsky 5059 (CM, NCU); Willow thickets around Connecticut Lake, Siplivinsky 2373 (CM). Otero Co., La Junta, Harvey 7520 (MONTU). Weld Co., Western shores of Union Reservoir, ca. 0.5 mi. N of State Hwy 119 in Longmont, Snow 6119 (KSP, MO); Greeley, Highland Nursery, Snow 7722 (GREE); New Windsor, Osterhout 1974 (RM), 2346 (NY) and 2347 (RM); Windsor, Osterhout 6365 (NY, RM); Crow Campground at Crow Creek, 1 mi.N of Briggsdale, Wingate 4533 (KANU). Yuma Co.; Wray, Osterhout 4070 (RM). Connecticut: Fairfield Co., Bridgeport, Beardslee s.n.New Haven Co., Near Union Depot, New Haven, Setchell s.n. (UC 38741). New London Co., Old Lyme, Weatherby 4390 (US); Woodward s.n. (US). Deleware: New Castle Co., Port Penn, Commons s.n. (GH); Ab loco, Commons 155 (US). Florida: Bay Co., Crooked Island east, Tyndall Air Force Base, Johson 7970 (FSU). Brevard Co., 0.5 mi. W of I–95, immediately S of FL 50, edge of St. Johns Riv., SW of Titusville, Hall and Beeman 1901 (FLAS); In marsh, E side of Lake Poinsett, Chamberlain 17 (FLAS); Sect. 23 of St. Johns Nat. Wildlife Refuge, ca. 3.5 mi. W of Titusville, Rakestra s.n. (GA). Broward Co. E side of Hwy 27 just S of Griffin Rd. (Rte 818), ca. 14 air mi. WSW of Ft. Lauderdale, Anderson 9905 (FSU). Charlotte Co., Vicinity of Port Charlotte, Godfrey & Reinert 60966 (FSU, NCU); Old field, Frye C–67 (FLAS); Caloosa Experimental Range, USFS and Range Station, Adams 239 (GA); 15 mi.NNW of Fort Myers, frequent on wet sands of distrubed flatwoods, Kral 7521 (GH, NCU). Citrus Co., Crystal, along or in water along railroad, Combs 1004 (US). Collier Co., Rte 41, 1 mi.W of Monroe Station, Hill 2754 (SMU); Naples, Godfrey 74458 (FSU, NCU); Fathlahatchee Swamp, J. H. Davis s.n. (FLAS); Fakahatchee Strand, Roadside, Janes Hwy, Avery and Churchill 2062 (FLAS); Fakahatchee Strand 7 mi.NW of route 29 junction, Churchill s.n. (SMU); NW of Copeland, Atwater 612 (FLAS); Vicinity of Bahama Ranch, off US 41, W of Everglades City, Lakela 31325 (GA, NCU); Remuda Ranch, W of Everglades City on Tamianmi Trail, US 41, extensive cut area in Big Cypress Swamp, Lakela 31723 (NCU, RM); Marco Island, Harvey 8203 (MONTU). Dade Co., Montgomery Foundation, E of Old Cutler Rd at a point E of end of SW 120 St, ca. 3 mi. S of South Miami, Orzell 22042 & Bridges (BRIT). About ponds along road just E of Homestead Airforce Base, Correll & Popenoe 49096 (NCU); Homestead, Herndon 905 (BRIT, RSA); Long Pine Key, Atwater GS–149 (FSU); W of Homestead, Avery & McPherson 1691 (FLAS); Marl Prairie, Davis s.n. (FLAS 38437); Homestead, Byrd s.n. (FLAS 114443). Franklin Co. 6 mi. SW Carrabelle, White 176 (RSA); Cape St. George Island, ca. 0.7 mi. of Marshall House, Anderson 9949 (FSU); St. Vincent Island, just W of Tahiti Point on NE sector of Island, Anderson 8964 (FSU); Dog Island, in W sector of Cannonball Acres, Anderson 5907 (FSU); St. Vincent Island, 1.8 mi.W of Tahiti Beach, Anderson 8580 (FSU). Glades Co., Hicpochee, Beardsley s.n. (FLAS 50590); Hendy Co. 12 mile Slough, Davis s.n. (FLAS 38438). Hillsborough Co., ca. 12 mi. SE of Tampa off US 41, Ray et al. 10615 (US). Lee Co., Sanibel Island scrub along Caloosa Drive, Churchill 7952 (ASU); Middle Sanibel Island, Brumbach 5374 (FLAS); W Sanibel Island, Brumbach 6964 (GH, FLAS); Upper Captiva Island, Brumbach 9160 (BRIT, FLAS, GA, GH, MICH, NCU); Central Sanibel Island, Brumbach 6960 (FLAS); 5 mi. E of Ft. Meyers Beach, Kral 7543 (GH, FLAS, NCU); 4 mi.N of Ft. Myers, Godfrey 65411 (FSU). Manatee Co., Brandenton Beach, Godfrey 65221 (FSU). Monroe Co., Loop Rd. 94, Correll et al. 42238 (LL); Flamingo, Everglades N.P., Atwater GS–162 (FLAS); Moist ground near Watson Hammock, NW side Big Pine Key, Thorne 15030 (GH); Big Pine Key, around small pond and brook, Swallen 5174 (US). Palm Beach Co., 18 mi. E of Belle Glade on FL 80, Dusky s.n. (FLAS 150015). Pinellas Co., Near Maximo Pt., St. Petersburg, Thorne 15440 (FSU, US). Sarasota Co., 3.5 mi. N of Englewood on Rte 775, Brass 28964 (NCU). St. Johns Co., Guana Lake, 10 mi. N of St. Augustine, Simons s.n. (FLAS 134057). Sarasota Co., 3.5 mi. N of Englewood on Rte 775, Brass 28964 (FLAS). Seminole Co., 2 mi. E of Geneva, Kral 5098 (FSU, GH, SMU); Upper St. Johns Riv., ca. 4 mi. S of FLA 46, Auth 62 (FLAS). Wakulla Co., Near Oak Island, Godfrey 58904 (FSU); St. Mark’s Wildlife Refuge, Godfrey & Kral 54080 (FSU, GH, NY); St. Mark’s Wildlife Refuge, Godfrey 55753 (FSU, NCU). Idaho: Canyon Co., Right side of Currant Island, T1N, R3W, Sec. 35, NE ¼, Dixon 114 (KANU); In seed from Forage Genetics, 812 First St., Nampa, Robinson s.n. (MU). Owyhee Co., Moist sandy soil along the Snake Riv. at Walters Ferry, Baker 8591 (US). Illinois: Adams Co., Shore of Mississippi Riv., at ferry loading, W of Meyer, Evers 78417 (MO). Alexander Co., Horseshoe Lake, Huston 71 (MO). Bureau Co., W side of Illinois River, decommissioned IL Rt. 26 bridge right-of-way, Hill 20819 (BRIT). Calhoun Co., Shore of Mississippi Riv., Cap au Gris bluff SW of Batchtown, Evers 89684 (NCU); Sand bar, Royal Landing, E of Brussels, Evers 98144 (MU). Champaign Co., Urbana, Ahles 7426 (ARIZ, BAA, CM, GH, KANU, NCU, OKLA, PR, RM, SMU, UC, US, W). Clark Co., Streets [of] Marshall, Bock 15 & Chase (SMU). Cook Co., Chicago, Moffatt 554 (GH, OKL); Berm of US Rt 6, 0.5 mi. E State Rt. 7 and W of 180th Ave, Cusick and Furlow 23702 (MICH); Buffalo Woods, junction of 87^th^ St and US Rt. 45 (96^th^ Ave.), Hill 28798 & Tessene (BRIT). Grundy Co., W side of Aux Sable Creek at turf farm, between Minooka Rd and Route I-80, ca. 2 mi. due W of Minoka, Hill 36535 (BRIT, MU). Jackson Co., Mississippi Riv. shore, at Station 7, Evans 642 (MO); cinder–bed along railroad, Urbana, Ahles 7560 (NCU). Jefferson Co., Railroad tracks at crossing of IL State Hwy 15, ca. 4.8 mi. W of intersection with Interstate Hwy 57, ca. 4.7 mi.W of Mt. Vernon, Snow 5842 (MO). Jo Daviess Co., Weedy areas along railroad tracks in cut through Maquoketa Shale, on W side of Scales Mound, Nee 22019 (KANU, NY). La Salle Co., Salt marsh along creek, in bottoms of Illinois Riv., 4 mi. E of Starved Rock, Steyermark 40618 (GH). Madison Co., Pond shore, 2 mi. SW of Poag, Evers 77839 (MO). Monroe Co., S of Fults, Mississippi Riv. floodplain, Bailey & Swayne 3072 (NCU). Randolph Co., Kaskaskia Island, W of Mississippi Riv., around slough, near Dozaville, Vincent & Thomas 8081 (MU). Sangamon Co., ¾ mi. S of Buckhart, Shildneck 15699 (MU). Will Co., NE of Channahon, at SW corner of intersection where US 6 goes over US 66 (I-55), Schulenberg 73-351 (BRIT). Wyandot Co., Along Co Rt 99, SW of Co Rt 16, NE of Carey, Cusick 35680 (MU). Indiana: Adams Co., Weedy berm of US Rt 33 at SE limits of Decatur, Cusick 29817 (MICH). Allen Co., berm of St. Rt. 101 at S limits of Monroeville, Cusick 29815 (CM); Median of US Rt. 30 at junction of Ternet Rd., N of Tillman, Cusick 25808 (MICH, NY). Benton Co., ca. 0.5 mi. of Otterbein off US Rt. 52 along the N and W railroad, Brant & O’Donnell 953 (CM, MO). Jay Co., berm of State Rt. 67, 1–1.5 mi. E of Trinity, Cusick 28473 (MICH). La Porte Co., On S side of I-94 just E of US 421, Schulenberg 74-284 (BRIT). Newton Co., N of Foresman, S of Mt. Ayr, NE of Brook, at the bend in Ind 55 at SE corner of Sec. 35, T29N, R8W, Schulenberg 76-466 (BRIT). Porter Co., Porter, SW corner of junction Hwy 49 and Hwy 20, Reznicek et al. 8321 (ISC, MICH). Steuben Co., Along US Rt 20, 1 mi. E of Angola, Cusick 36709 (MU). Van Wert Co., Junction of St. Rt 66 and Fifth St., W limits of Delphos, Cusick 29796 (CM). Iowa: Clay Co., SW margin of Mud Lake, Hayden 13 (GA, MICH, MO, MONT, NMC, NY, SMU, UC, US); W margin of Mud Lake, Hayden 87 (GH, MO, NY); Gravelly, sandy shore of Round Lake, Hotchkiss 4542 (US). Dubuque Co., Waste ground along Mississippi Riv., N of Dubuque Harbor, Cusick 31183 (GH, VPI). Fremont Co., Gravel road bearing N from IA Hwy 2, ca. 0.4 mi. E of bridge over Missouri Riv., ca. 1 mi. N on the gravel road, Snow & Koster 5824 (MO); Bartlett State Wildlife Mgmt Area, small lake in sandy barrow pit NW of exit off I-29, Freeman 20981 (KANU). Harrison Co., In DeSoto Bend Wildlife Refuge, Sutherland and Kaul 3984 (NY). Mills Co., Pacific Junction, 2 mi. S of Keg Creek Lake Wildlife Mgmt Area, Freeman 20932 (KANU). Monona Co., Onawa, 4 mi. W, 1 mi. W of Louisville Bend Wildlife Mgmt Area, Freeman 23050 (KANU). Pocohontas Co., Pocahontas, Pohl 6929 (FSU). Polk Co., Pond adjacent to original Skunk Riv. bed, Van Bruggen 3324 (UC). Story Co., Ames, along railroad tracks near Iowa State Univ., Davidse 1923 (MO, US). Woodbury Co., Snyder Bend Wildlife Mgmt Area, Freeman 20891 & Morse (KANU); Dessicated margin of Brown’s Lake, Sec. 33, 2 mi. W of Salix, Thorne 16342 (BRI, NCU); Rest Area beside U.S. Hwy 59 at Seven Oaks S of Corregan, Thomas & Thomas 153315 (GREE). Kansas: Allen Co., Kirschner farm, 2 mi. E of Humbolt, Dubois 229 (US). Anderson Co., 2 mi. N of Garnett, Wilson 6942 (KSTC). Atchison Co., 2 mi. N of Atchison, McGregor 29881 (KANU). Barber Co., ¼ mi. S of Elm Mills, Stephens 19289 (KANU); Barber County State Lake, McPheeters 93 (KSTC); State Lake Medicine Lodge, Hall 83b (KSTC); State Lake, Greiner 220 (KSTC) and Wilson 7068 (KSTC). Barton Co., Cheyenne Bottoms, Wilson 4512 (KSTC); Cheyenne Bottoms, ca. area 34, Wilson 4559 (KSTC); Cheyenne Bottoms, Lindstrom 752 (KSC, OKLA); Cheyenne Bottoms, ¼ mi. NE of Dock #7, Wilson 4527 (KSTC) and 4542 (KSTC); ibid. except “Area 19”, Wilson 4271(KSTC); Cheyenne Bottoms, Prather s.n. (KSTC 011252); 2 mi.SE Redwing, area of the Cheyenne Bottoms, McGregor 12652 (KANU, US); Cheyenne Bottoms, SE corner of Pool 2, Hoffman s.n. (KANU 17922); 2 mi. S, 5 mi. E of Hoisington, Cheyenne Bottoms Nature Conservancy Reserve, near Rush Lake, Morse 1585 & Loring (KANU) and (ibid.) 1599 (KANU); Great Bend, Runyon 323 (CM, FLAS, MICH, RM); Hoisington, 1.0 mi. S, 2.0 mi. E of Cheyenne Bottoms Preserve, Freeman 5354 (KANU). Bourbon Co., Fort Scott (8 mi. W of, on US 54), Wilson 6600 (KSTC). Brown Co., 1 mi. N of Horton, McGregor 29872 (KANU). Butler Co., 5 mi. N of El Dorado, McGregor 29495 (KANU). Chase Co., Chase County State Lake, McGregor 29483 (KANU); NW edge of Lake Kahola, Wilson 8061 (KSTC). Chautauqua Co., 1 mi. S, 2 mi. E of Chautauqua, McIntosh 84 (KANU). Clay Co., 1 mi. W of Clay Center, Stephens 29473 (KANU); Broughton, 1 mi. S, 0.5 mi. W, Freeman & Elliot 7555 (KANU); Clay Center, 1 mi. S along KS 15, Freeman & Elliot 7747 (KANU). Cloud Co., Ditch on corner 1 mi. E of S side of salt marsh 4 mi. E of Jameston, Fraser 300 (KSC); 1 mi. NW of Old Lake Schley, Fraser 631 (MICH); R4W, T5S, NW ¼ sec. 17, Ungar 1118 (KANU); Concordia, 1 mi. N along US 81 on N. Side of Republican River, Freeman & Elliot 7646 (KANU). Coffee Co., 2 mi.S & 1 [mi.] W of New Strawn, Magrath 1198 (KSTC); 1 mi. S of Crandall, McGregor 28645 (KANU). Cowley Co., 8 mi. E, 2 mi. N. of Arkansas City, Koch 1604 (KANU, OKLA); 7 mi. W of Winfield, Koch 1815 (KANU); 7 mi. W of Winfield, Koch 2102 (OKLA). Dickinson Co., At rest stop along Interstate Hwy 70, exit 266, ca. 10 mi. W of Abiline, Snow & Koster 5840 (MO); ab. loco, Hitchcock 920 (KSC, RM); 1 mi. S, 1 mi. E of Sutphen, McGregor 37876 (KANU). Doniphan Co., 2 mi. S. of Denton, Denton 141 (KSTC). Douglas Co., 10 mi. N of Lawrence, Ungar 1037 (GH, NCU, SMU, US); 13 mi. S of Lawrence [at] Baldwin Junction, Brooks 12449 (KANU); 3 mi. E of Lecompton, McGregor 29904 (KANU); 4.5 mi. S, 0.5 mi. E of Clinton Reservoir, Freeman 21972 & Freeman (KANU); Clinton Lake State Park, McGregor 41148 (KANU); Clinton Lake, T13S, R18E, NW ¼ Sec. 23, McGregor 39597 (KANU); ca. 0.2 mi. ESE of the Bowersock Dam, Freeman 2718 (KANU). Edwards Co., 3 mi. SW of Kinsley, McGregor 10877 (KANU). Elk Co., 12 mi. E, 6 mi. N of Howard, McGregor 28613 (KANU). Ellis Co., Saline Riv., Dutt 104 (MO). Ellsworth Co., Kanapolis Reservoir north shore, McGregor 37906 (KANU); ab. loco, Weber 275 (KSC). Finney Co., 1 mi. E, 6 mi. N of Kalvesta, Stephens 63120 (KANU); South edge of Garden City, Stephens 87367 (KANU); Garden City, Condray s.n. (KSC [0027307]). Ford Co., 6 mi. E, 1 mi. S of Dodge City, Stephens 50479 (KANU); 1 mi. NW of Ford, McGregor 10901 (KANU). Geary Co., Grandview Plaza, 1.5 mi. NE Ft. Riley Military Reservation, Main Post, N side of Kansas River just W of Henry Drive, Freeman 19538 (KANU); Ogden, 0.5 mi. S., Freeman 12864 (KANU); Burel City, E side of town, Oxbow of Smoky Hill River on NW side of I-70, Freeman 19383 (KANU). Gove Co., West edge of Quinter, Stephens 86677 (KANU). Graham Co., Hill City, S edge of town along US Hwy 283 at the South Fork Solomon River, Freeman 8440 (KANU). Gray Co., 0.5 mi. S of Cimarron, Stephens 50420 (KANU, NY). Greeley Co., 9 mi. E of Tribune, Brooks 8570 (KANU). Greenwood Co., Roadside along Hwy 99 near Verdigris River, Hauser 808 (KSTC). Fall River Reservoir, McGregor 28636 (KANU). Hamilton Co., 1 mi. S of Kendall, Stephens 50385 (KANU); 0.5 mi. S of Coolidge, Stephens 87414 (KANU). Harvey Co., 3 mi. E, 2 mi. N of Burrton, Stephens 19162 (KANU). Haskell Co., 6 mi. N, 10 [mi.] W of Sublette, Stephens 42506 & Brooks (KANU). Jefferson Co., Old town area of Perry Lake, McGregor 39627 (KANU); SW edge of Perry Bank of Deleware River, McGregor 29776 (KANU); Newman, 0.7 mi. S, 0.5 mi. W, Russell & McGregor 474 (KANU). Jewell Co., 3 mi. S of Lovewell, Stephens 86129 (KANU); Lovewell State Park, Lindstrom 149 (KSC). Johnson Co., 4.5 mi. E., 4 mi. N of DeSoto, Brooks 12809 (KANU). Kearney Co., 1 mi. S of Lakin, Brooks 10645 & Hauser (KANU); 4 mi. NE of Lakin, Stephens 87391 (BRIT, KANU); McKinney Lake, just NE of Lakin, Thieret 16259 (MU). Kiowa Co., 0.5 mi. N of Haviland, Stephens 63278 (KANU). Labette Co., Parsons, 1 mi. E, 4 mi. S, KS Army Ammunition Plant, Freeman 6639 (KANU); Parsons, E-central part of town, Freeman 20530 (KANU); vicinity of Parsons, Gwartney 11 (KSC). Leavenworth Co., Ft. Leavenworth Military Reservation, E of S end of Sherman Army Airfiled, Freeman 7467 (KANU). Logan Co., Chalk Bluff & Smokey Hill River, Wilson 5885 (KSTC); Logan County State Lake, Brooks 7298 (KANU); Russell Springs, 8.5 mi.E, 2 mi. S of Smoky Valley Preserve, Freeman 13904 (KANU). Lyon Co., 0.5 mi.N, 1 mi. E of Emporia, Schaefer 1057 (OKL); Emporia, Willis s.n. (KSTC 011264); 1 mi. S of Emporia, Eckert 147 (KSTC); 12 mi. N of Emporia, lake bottom, Osborne 110 (KSTC); 1 mi. N of Emporia on Rd to Reading, Wilson 2630 (KSTC); ½ mi. E of Emporia, Wilson 2716 (KSTC); Peter Pan Park, King s.n. (KSTC 011282); McKenney Marsh, 15 mi. SE of Emporia, Greiner 249 (KSTC); ½ mi. N of US 50 bypass at edge of pond along Burlingame Rd, Lickteig 49 (KSTC); N of 18^th^ St and E of Hwy K-99, Emporia, Spohn 32 (KSTC); N of KS State Teaching Collecge campus waste area, Spohn 33 (KSTC); McKenney Marsh Bank, Nelson 153 (KSTC); Emporia, East Lake area, Husband 79 (USCH); Hartford, 1.5 mi. N, 3 mi. W, Freeman 18097 & Morse (KANU); 0.5 mi. W., 1 mi. N of Reading, Stephens 83330 (KANU). Marion Co., Marion Lake, T19N, R3E, NE ¼ Sec. 22, McGregor 39729 (KANU). Marshall Co., West edge of Marysville, Stephens 86039 (KANU). Meade Co., 0.75 mi. S of Meade, Horr 4457 (GH); 0.75 mi. S of the E side of Meade, Horr & McGregor 4069 (US); 8 mi. S of Meade, Stephens 63022 (KANU); ¾ mi. S of Meade, Horr 4457 (KANU). McPherson Co., McPherson County State Park, McGregor 25894 (KANU). Mitchell Co., 15 mi. S, 2 [mi.] W of Beloit, Stephens 87131 (KANU). Morris Co., Council Grove Reservoir, T16N, R8E, NW-1/4 sec. 2, McGregor 39693 (KANU). Montgomery Co., Eastep Property, ca. 8.5 km NW of Cherryvale, Snow 10978 (KSP); Walnut, Holland 510 (KSC). Morton Co., Cimarron Nat. Grassland, T33S, R41W, sec. 27, McGregor 38416 (KANU); 10 mi. N of Elhart, K-27, Garner 924 (KSTC). Nemaha Co., Nemaha County State Lake, McGregor 29837 (KANU). Neosho Co., 1-1/2 [mi.] S of Erie on levee near Neosho Riv., Holland 663 (KSP); 2 mi. E, 0.25 mi. N of Chanute on K39, Holland 9975 (KANU, KSC); 2 mi.W, 1-3/4 mi. S of Walnut, Holland 1518 (KANU); ca. 3.5 mi. SE of St. Paul, Mission Township, Neosho Wildlife Refuge, Holland 9490 (KANU, KSC). Ness Co., Center of Ness City, McGregor 33518 (KANU). Norton Co., 5 mi. SW of Norton, McGregor 30475 (KANU); Near Almena, Bockelman 2473 (KSC). Osage Co., 4 mi. N of Quenemo, Brooks 12419 (KANU). Ottawa Co., Minneapolis, S edge of town, T11S, R4W, sec. 1, SE ¼, Freeman 9804 (KANU); 4 mi. SW of Wells, edge of State Lake, McGregor 23585 (KANU). Pawnee Co., 1.5 mi.W of Rozel, Stephens 63178 (KANU). Pottawotomie Co., In Roadside ditch 1 mi. N of Louisville, Bartley 1187 (US); County State Lake No. 2, McGregor 29621 (KANU). Pratt Co., Iuka, 4.0 mi. N on US Hwy 281, Freeman 5338 (KANU, OKL); 11 mi. N of Pratt, Barker 1793 (KANU). Rawlins Co., 14 mi. N of McDonald, McGregor 30836 (KANU). Reno Co., Reno-Harvey county line, Wilson 8415 (KSTC); 1.5 mi. W of Nickerson, Stephens 34632 & Brooks (KANU). Republic Co., Sec. 28, T4S, R2W, Elliot 1159 (KANU). Rice Co., 2-3/4 mi. S of Alden, Brooks 17036 (RM); 1 mi. S of Raymond, Stephens 34586 & Brooks (KANU). Riley Co., ca. 77 m W of Kansas State Hwy 18, ca. 3.5 mi. N of Interstate Hwy 70 and ca. 7.3 mi.SW of Manhattan, Snow 6122 (KSP, MO); 11 mi. N of Manhattan, Cove of Tuttle Creek Reservoir, McGregor 29648 (KANU); Sandy floodplain of the Kansas Riv. W of Manhattan, Gates 13642 (MO, MU); Ogden, ½ mi. WSW of Fort Riley Military Reservation, Funston Monument, Freeman 19342 (KANU); Manhattan, Johnston s.n. (KSC [0027275]). Rooks Co., Woodston, 1.7 mi. W of Woodston Diversion Wildlife Area, Freeman 10342 (KANU); Damar, 5 mi. E, 4 mi. N, south side of Webster Reservoir, Freeman 10094 (KANU); E edge of Damar, Stephens 86882 (KANU). Rush Co., 1 mi. S of Bison, McGregor 30548 (KANU). Russell Co., 8 mi. S of Lucas, Wilson Reservoir Area, McGregor 29376 (KANU). Saline Co., 5 mi. S, 5 mi. E of Brookville, Elliot 1766 (KANU); northeast of Kipp, Hancin 507 (KSC); T15S, R4W, Sec 27, NE4, Barnard 1318 (KSC). Sedgewick Co., 0.5 mi. N of Cheney Reservoir, Hauser 2558 & Brooks (KANU); Along Arkansas Riv. at 21st and West Street near I-235 bridge, Walter & Sisco 9600 (MO); Along the Santa Fe railroad in Wichita, Bartley 1224 (US); S. side of the Kansas Riv. in Lawrence, ca. 0.2 mi. ESE of the Bowersock Dam, Freeman 2718 (RM). Sheridan Co., Hoxie, Hauser 3009 (KANU); Selden, Weber 13 (KSC). Seward Co., 5 mi. W of Kismet, Stephens 45749 (KANU); Cimarron River, NE of Liberal, Lindstrom 625 (KSC). Stanton Co., 2 mi. SW of Johnson, Stephens 87436 (KANU); 2 mi. N., 1 mi. E of Saunders, Stephens 87455 (KANU). Sumner Co., T32S R4W, Sec 36, NW ¼, Birkholz 1018 (KSTC) and Birkholz 1332 (KSTC); T30S R2E, Sec 21 SW ¼, Birkholz 1006 (KSTC) and Birkholz 1609 (KSTC); 6 mi. S of Geuda Springs, Brooks 11033 & Hauser (KANU). Wichita Co., 7 mi. N of Leoti, Brooks 8560 (KANU); Wichita, County Park S of 21^st^ Street and E of Ridge Road, Freeman 12938 (KANU). Shawnee Co., Topeka, Smyth s.n. (KSC [0027306]); 1 mi. NW of Tecumseh, McGregor 29740 (KANU); ca. 3 mi. W of Richland, Sec. 25, T13S, R16E, McGregor 40909 (KANU). Wabaunsee Co., At roadside off Exit 338 along Interstate Hwy 70, ca. 27 mi. W of Topeka, Snow & Koster 5841 (MO); Lake Wabaunsee, McGregor 29575 (KANU). Wallace Co., 2.5 mi. N of Sharon Springs, Stephens 45682 (KANU). Woodson Co., Woodson County State Lake, Brooks 12772 (KANU); 2 mi. S. of entrance to Lake Fegan recreation area, at concrete river forge, Wilson 2227 (KSTC); Lake Fegan, E. side by dam, Woodson 6498 (KSTC). Wyandotte Co., ¾ mi. NW of Pomeroy, Brooks 11855 (KANU). Kentucky: Campbell Co., Silver Grove railroad yard, Buddell 439 (NCU, NY); Silver Grove RR yard, Buddell 439 (MU). Fulton Co., Hickman, edge of Pond Slough 2.5 mi .on Ky 971 from Ky 94, Athey 3796 (FSU, WKY); Hickman, sandy roadside ditches, KY 94, 2.4 mi. W of junction of KY 1099, Athey 2015 (GH, NCU, NY). Hickman Co., Railroad shipping yard at Famers Gin in Clinton, Grubbs 11781 (GA). Kenton Co., River Rd and Traverse St., Ludlow, Cusic 36106 (MU). Ohio Co., “Beaver Dam”, Hils 95 (NCU). Louisiana: Calcaseiu Par., 3.0 mi.S of junction of LA Hwys 27 and 14 along LA Hwy 27, ca. 10 mi. SE of Lake Charles, Snow 5804 (MO). Cameron Par., 2.2 mi. W of Rockefeller Refuge Headquarters, Reese 6119 (FSU, SMU); Edge of fresh marsh ca. 2 mi. S of Gibbstown bridge along Hwy 27, McKenzie 143 (MO); On beach, along Holly Beach, Correll & Correll 9606 (GH, US). LaSalle Par., Old Riv. beside US Hwy 84, 9 mi. SE of Jena, Rogers 2548 (NCU). Morehouse Par., Along railroad tracks S of Hayne Avenue and E of Washington St. (LA Hwy 139) in Bastrop, Thomas 121085 (GREE); Railroad yard N of US 165 and Cypress St and E of US 425 and Washington Plaza in Bastrop, Thomas 135981 and Cheatham (MU). Natchitoches Par., N side of Red Riv. at the LA 6 bridge N of Natchitoches and Grand Ecore, Thomas 114844 (MO, NY). Orleans Par., Lake Ponchatrain, Brown 2384 (US). Plaquemines Par., LA Hwy 23, 2.5 mi. N of Mytrle Grove, 12.9 mi. S of US Naval Air Station, Belle Chase, Mississippi Riv. Alcohol Company grounds, in ditch along entrance road, Snow 5786a (MO). West Baton Rouge Par., West Mississippi Riv. Banks, directly across from Baton Rouge, 0.5 mi. E of LA Hwy 1, Snow 5800 (MO). Maryland: Allegany Co., S side of Rt I-68 at Exit 34, St Rt 68, S of Frostburg, Cusick 37524 (MU). Garrett Co., S side of Rt I-68 at Exit 4, St. Rt 42 ramp to Friendsville, Cusick 37517 (MU). Worcester Co. Sand flats bordering Assawoman Bay, Ocean City, Hermann 10725 (GH, MO); wet sandy border of brackish marsh, 1.75 mi. N of Ocean City, Fogg 11411 (GH). Massachusetts: Nantucket Co., Nantucket Island, Sesachacha Pond, MacKeever 810 (US). Suffolk Co., Boston, near Audubon Road, Swan s.n. (SMU); Boston, Swan s.n., 17 Sep 1887 (MU 000078391). Michigan: Berrien Co., NE of Niles, in Penn Central RR classification yards, Schulenberg 73-433 (BRIT). Monroe Co., Public access site on Halfway Ck., near center E 1/2 sec. 33 Erie Twp., ca. 3.5 mi. SE of Erie, Reznicek & Voss 7842 (MICH); 1 mi.W of Azalia, in median of US 23 immediately S of the Cone Rd. interchange, Reznicek 5743 (MICH). Lenawee Co., Along railroad N of Maple St., E of Dean St., Adrian, Smith 2740 (MICH). Saginow Co., SE side of the Saginaw Riv. on disturbed Roadside of M–13 ca. 1.5 NE of Zilwalkee Bridge, Garlitz 3375 (MICH). St. Clair Co., Port Huron Twp., NE 1/4 Sect. 17, large railyard on SE side Port Huron, Reznicek & Schultz 5415 (MICH). Washtenaw Co., Ann Arbor, N side of Maiden Lane just W of junction with Maiden Lane Ct., Reznicek 4954 (MICH).Wayne Co., 3 mi. NE of Romulus, median of Interstate 94, ca. 0.25 to 2 mi. W of the interchange with Merriman Rd., Reznicek 4973 (GH, MICH); Riv.view, W Jefferson Ave., at Grosse Ile tollbridge, Katz 787 (MICH). Minnesota: Pipestone Co., Pipestone, Fellows 56a (CM, US); Edge of city park, Pipestone, Moore 22961 (US). Wilkin Co., ca. 10 mi. S of Foxhome, W of Rte. 19, Wheeler 12062 (MICH). Mississippi. Harrison Co., Cat Island, Tracy et al. 149 (MO); Cat Island, Tracy 9195 et al. (SMU). Jackson Co., Fontainebleau, Demaree 34099e (GH, US). Washington Co., E of Prisilla, Elmore s.n. (FLAS). Missouri: Atchison Co., Edge of a parking lot, in Rock Port, Henderson 94–504 (MO); Along railway grades, near Watson, Palmer 18903 (KSC). Barry Co., 2.5 mi. N of Purdy, Palmer 66390 (GA, SMU). Buchanan Co., Sugar Lake, 3 mi.E of East Atchison, Steyermark 15222 (MO); St. Joseph, 0.5 mi. WNW, French Bottom, Freeman 23576 (KANU). Butler Co., S side of State Hwy 142, ca. 1.2 mi. W of junction with County Rd. 341, ca. 3.5 mi.E of US Hwy 67 in Nellyville, Yatskievych et al. 94–212 (MO). Cameron Co., Burlington Lake, 1 mi. S of Cameron, Steyermark 14927 (MO). Cape Geirardeau Co., Missouri Honkers boat dock, Brooks & Gaither 7223 (BRIT, KANU). Carroll Co., Mud flats along Missouri Riv., 9 mi. S of Carrollton, Steyermark 14864 (MO). Chariton Co., Swan Lake Nat. Wildlife Refuge entrance ca. 5 mi. S of Swan Lake, Harmon 339 (GREE); ca. 2.5–3 mi.S, 0.5-1.5 mi. E of Sumner, Swan Lake National Wildl. Ref., Morse 11706 (KANU). Christian Co., 2.5 mi. W of Sparta, Palmer 57969 (NY, SMU). Clay Co., Missouri City, common on muddy shores, MacKenzie s.n. (MO 2972233). Cole Co., Jefferson City rail yard, near Missouri Riv., Raveill 2160 (MO). Cooper Co., in saline soil around Chouteau Springs, 10 mi. SW of Boonville, and S of Lamine, near Choteau Ck., Steyermark 15915 (MO). Dunklin Co., Campbell, low woods, Kellogg s.n. (MO 2194418). Greene Co., Vicinity of Battlefield, Standley 9581 (US). Holt Co., Big Lake State Park, 3 mi.W of Bigelow, Steyermark 15125 (MO). Howard Co., Around saline springs at Boonslick, 3 mi. W of Boonsborough, Steyermark 14791 (MO). Jackson Co. Kansas City city limit, intersections of Marsh Ave and E Truman Rd., ca. 150 m E of intersection of E Truman Rd. with I-435, Snow & Koster 5823 (MO). Jasper Co., Near “Oakland”, 3 mi. NE of Joplin, Palmer 32551 (MO, NY); Joplin, Palmer 2509 (NY, US). Lafayette Co., Along a railroad right-of-way at Co. Rd. Z, at the S edge of Bates City, Henderson 92-404 (MO); Lewisville, Sundel 12326 (MU). Lincoln Co., North Platte, bed of North Platte Riv., Kiener 17457 (UC); Prairie Slough Conservation Area, ca. 5 mi. NE of Elsberry, E of Rt. P and NNW of Westport Island on the Miss. Riv., McKenzie & Jacobs 1628 (MO). Mississippi Co., US Hwy 60 on Missouri side of Mississippi River, Harmon 1747 (GREE). Moniteau Co., 6 mi.NE of Jameston on a sandy island in the Missouri Riv., Harmon 1774 (GREE). New Madrid Co., near La Forge, Steyermark 8853 (MO). Newton Co., Grand Falls, 3 mi. W of Redings Mill, Palmer 58866 (GA, KANU, KSC). Marion Co., Bear Ck. bottoms, Hannibal, Davis 3449 (MO). Pemiscot Co., Intersection of I–155 and Co. Rd. U overpass, McKenzie 1163 (MO). Pettis Co., Along a railroad right–of–way at MO 127, in La Monte, Henderson 92–388 (MO); 4.5 mi. W of Steele on Hwy 164 at junction with C, Summers 5370 (MO). Pike Co., At Clarksville, Evers 89860 (MO). Platte Co., Low Roadside, along MO 45, ca. 1 mi. S of Farley, Henderson 94–522 (MO). Ralls Co., Around margin of salt–sulphur spring lake at Spalding, Steyermark 25692 (MO). Randolph Co., Around saline soil of lake area at Randolph Springs, between Clifton Hill and Huntsville, Steyermark 16040 (MO). Saline Co., Missouri Riv. bottoms, opposite Glasgow, Steyermark 9391 (MO, UC). Scott Co., 1 mi. N of Commerce, Brooks & Ohmart 7597 (BRIT, KANU). St. Charles Co., ca. 1.5 mi. W of Defiance, along high sand bars of Missouri Riv., Snow 5845 (MO); Below US Hwy 40 bridge over Missouri Riv., first floodplain terrace on N bank of Riv., Snow 6003 and 6016 (MO). St. Clair Co., Shore of White Sulphur Spring, 4.5 mi. NE of Iconium, Steyermark 24416 (MO, NY, POM, US). St. Genevieve Co., Us Rt 61, S of St. Mary post office, Cusick 33293 (MU). St. Louis Co., Jefferson Barracks County Park, adjacent to railroad tracks paralleling Mississippi Riv., Snow 5781, 5783 (MO); St. Louis, Junction of I–170 and State Hwy 340 (Olive Blvd), Snow 5820 (MO); Along railroad tracks ca. 50 m N of track’s junction with Stein Street, just W of Mississippi Riv., Snow 5821 (MO); Along railroad tracks, E edge of Jefferson Barracks County Park, beyond end of Gark Rd., Snow 5822 (MO); St. Louis, along railroad tracks paralleling Manchester Rd., just S of intersection with Sublette Ave., Snow 5996 (KSP, MO, MU). Stoddard Co., along railroad tracks at the grain elevator in downtown Dexter, Ladd et al. 13604 (MO); T25N, R12E, near centre point E boundary Sec. 12, Brant and Gereau 1125 (MO). Stone Co., Piney Creek Wilderness, around Table Rock Lake, SW ¼, Sec. 19, R24W, T21N, Rebman 493 (ASU). Montana: Big Horn Co., ca.10 mi. S of Custer, Lesica 8038 (MONTU). Phillips Co., Ephemeral pond near Beaver Ck. ca. 25 mi. SE of Malta, Lesica 4590 (MONTU, NY); Malta, D. Messner s.n. (MONT). Richland Co., Seven Sisters Wildlife Management Area, ca. 15 mi. E of Crane along Yellowstone River, Heidel 1568 (MONT). Roosevelt Co., SW end of Horstead Lake, 5 mi. NW of Froid, Hotchkiss 6965 (MONT). Yellowstone Co., W edge of Billings just N and E of intersecton of Danford Rd and 56^th^ St., McEldowney s.n. (MONT). Nebraska: Burt Co., Uct of US Rt 75 & St Rt 51, Cusick 36392 (MU). Cass Co., N of Louisville on Hwy 50, along the Platte Riv., Churchill s.n. (MO 2619370). Clay Co., 1 mi. W and 1.5 mi. N of Ong, Churchill and Albert 2079 (MO). Cuming Co., 0.5 mi. W of West Point on Hwy 32, along Elkhorn Riv., Churchill 4809 (MO). Dawson Co., Channel of Platte Riv., S of Lexington, Kiener 17236 (GH). Hall Co., 5 mi. E of Grand Island, Tolstead 41638 (UC). Jefferson Co., 4 mi. NNW of Gladstone, Churchill 7989 (BRIT, KANU, MO). Kearney Co., Hapeman s.n. (POM); “Prairies and sandhills”, Rydberg 6512 (NY, RM); Minden, Hapeman s.n. (ARIZ 147351; MU barcode 000078455; OKLA 11887). Keith Co., S fork of Platte River, near Ogallala, Lindstrom 926 (KSC, OKLA). Lancaster Co., E shore of Oak Lake, just W of Lincoln, Churchill 2631 (MO); Lincoln, 27^th^ St and Hwy 80, Ungar 1178 (KANU). Merrick Co., Just S of Silver Creek on Hwy 39, T15N, R3W, sec. 4, Churchill 6648 (KANU). Richardson Co., Lake bed of the Verdon Lake, W of Verdon, Reynolds 2285 (MO); East shoulder of Rulo-White Cloud Road, Shildneck C-6922 (KANU); SE bank of cut-off lake between levee and west bank of Missouri River, SE corner of S29, R18E, T2N, Shildneck C-6802 (KANU). Thomas Co., On Middle Loup Riv., near Thedford, Rydberg 1713 (NY, US). Webster Co., 1 mi. S of Red Cloud, Stephens 51200 (KANU). Nevada: Churchill Co., Lahontan Valley, Stillwater Point Reservoir Diversion Canal, Tiehm 12743 (ASU, MONTU). Clark Co., Las Vegas, Corner of Mayflower and Falcon Lanes, Bostick 5002 (ARIZ). Douglas Co., Carson Valley, near Minden, Archer 6746 (GH, NA, NY). Elko Co., Hot Springs 0.5 mi. S Elko along Humboldt Riv., Holmgren 1872 (ARIZ, NA, UC, W). Humboldt Co., Sheldon Nat. Wildlife Refuge, Virgin Valley, Pond 21, 0.7 air mi. SE of Range HQ Duffurena Ranch, Tiehm & Rogers 4684 (NY). Nye Co., Nevada Test Site, Vicinity of drainage channel from Warm Springs, Rt. 6, base of S Hot Ck. Range, Beatley 11826 (NY, US). Washoe Co., Pyramid Lake Indian Reservation, Tiehm 13411 (ASU, MONTU); Reno, Tracey s.n. (BRIT, KSC). New Jersey: Atlantic Co., Salt marsh pond, Ventor, Mackenzie 7372 (MICH, MO); Brackish meadows, Atlantic City, Parker 10925 (MO). Cape May Co., In sand, Cape May Point, Brown 102 (GH, MU). Salem Co., Clayey cornfield along Alloways Ck., 3.5 mi. W of Hancocks Bridge, Fogg 7734 (MO); tidal marsh along Alloways Ck., 3 mi. W of Hancock’s Bridge, Fogg 7730 (GH). New Mexico: Chaves Co., Roswell, Earle and Earle 330 (MO, NMC, NY, US, W). Dona Ana Co., Las Cruces, NW corner of NMSU campus, Spellenberg 4336 (NY) Franklin Mts., Anthony Gap, Worthington 17211 (NY); 1.5 Road mi. SW of the old Mt. Riley train watering stop at playa, Worthington 12715 (NY, UTEP); Potrillo Mts, W Potrillo Mts, playa along Columbus to El Paso Rd., ca. 2.5 km SW of the old Mt. Riley Station, Worthington 22368 (NY, UTEP). Eddy Co., Carlsbad, Tracy 8172 (NY). Grant Co., Phelps Dodge 1, Plot 14M, N32.89725, W108.59708, Loring 2823 (KANU). Guadalupe Co., 6.5 mi. N of Santa Rosa along the Pecos Riv., Higgins 8915 (ASU, NY). Hidalgo Co., Diamond A Ranch, Turner 2002-25 (ARIZ); Gray Ranch, just east of Headquarters along main road, Allred 6005 & Roalson (ARIZ); Gray Ranch, Turner & Turner 2004-35 (ARIZ); 7 mi. S and 2.4 mi. SE of McAllen, Shinners 17154 (SMU); Animas Valley, junction of NM 338 with NM 79, Worthington 14909 (NY); Vicinity of Lordsburg Playa on NM Hwy 338 immediately S of the Interstate–10 interchange, McIntosh 2257 (US); Upper San Simon Valley near Rodeo, ca. 6 mi. E of Portal Peak (AZ), Moir 11 (ASU). Lea Co., Playa lake S side of Hwy 62 and 180, ca. 10 mi. W of Hobbs, Correll & Correll 36060 (NY); 5 mi.N of Tatum, Goodding 2505 (ARIZ, MU). McKinley Co., 5.5 mi. E of US Hwy 666 on Najavo Hwy 9 to Crownpoint, Spellenberg 4835 (NMC, NY). Roosevelt Co., 10.5 mi. E of Elida, Stephens 80162 (KAN). Sandoval Co., Along NM 44, ca. 23 mi. NW of Bernalillo, Henderson 69–352 (FSU, MO, NCU); 2.4 mi.N of San Ysidro on Hwy 44, 32 air mi. NW of Albuquerque, Shultz & Shultz 1296 (GH, NY). San Miguel Co., Bell Ranch, Schiebout 8166 (GREE). Socorro Co., Socorro, Vasey 523 (US). Union Co., Clayton Lake State Park, Schiebout 7401 (GREE); USFS Rd. 34, Schiebout 7655 (GREE). New York: Cayuga Co. Salt flats, Montezuma, Metcalf 5717 (GH, MO, NY, US, W). Chataqua Co., Weedy ground at junction of State routes 5 and 76, N of Ripley, Cusick 29143 (MICH). Erie Co., Deadend of Fuhrmann Blvd. at Coast Guard Station, Buffalo, Cusick 29162 (MICH). Onondaga Co., Pleasants Beach, Onondaga Lake, Rowlee & Smith s.n. (MO 2972206). Rockland Co., Beside Continental Can Company parking lot adjacent to Piermont pier, Oragnetown Township, Lehr 1212 (NY). North Carolina: Brunswick Co., Smith Island complex, pond on Bluff Island, LeBlond 4073 & Weakley (NCU). Dare Co., Roanoke Island, Manteo, waste area around warehouse, Crutchfield 5474 (NCU); Bodie Island salt marsh, Burk s.n. (NCU). Davidson Co., 2.8 mi. S of Southmont, Ahles & Leisner 18728 (FSU, NCU). Hyde Co., Low marsh, usually exposed during summer, Lake Mattamuskeet, Rudolph 13–84 (GA); 5.9 mi. S of Fairfield, E of NC 94, Leonard 8559 (FLAS, FSU, NCU); Alligator River at NC 94, LeBlond 3030, 12 Sep 1992. North Dakota: Burleigh Co., Long Lake, Moffit, Stevens 380 (GA, OKL, UC, W). Cass Co., Fargo, Stevens 1587 (KANU, KSTC, NCU, UC, US); Fargo, Stevens 2517 (KANU, UC, US). Emmons Co., Mouth of Badger Creek adjacent to the Oahe Reservoir, 13 mi. W, 1 mi. N of Hazelton, Williams 1414 (KANU); 3 mi.S of Glencoe Church, Williams 653 (KANU). La Moure Co., W side of Hwy 281, SE 1/4 of the NE 1/4 of Sec. 3, Golden Glen Township, Edgeley, Moore & Moore 10071 (KANU, GH, NY, OKL, SMU, UC). Logan Co., Ashley, Stevens 120 (CM, NA, NCU, UC, US). McIntosh Co., 3.5 mi. W of Ashley, Larson 3901 (KANU); Lake Hoskins, 3 mi. W of Ashley, Williams 3201 (KANU). Sargent Co., 5 mi. S of Cayuga, Larson 3791 (KANU). Williams Co., 5 mi. N and 2 mi. E of Williston, Hegsted 8525 (KANU). Ohio: Ashtabula Co., Junction of State Rts. 193 and 531 E of Kingsville–on–the–Lake, Kingsville, Cusick 23976 (MICH, MU). Auglaize Co., Median of US Rt 33 at juct of Rt I-75, SE of Wapakoneta, Cusick 25821 (MU). Butler Co., Behind Middletown High School, Breiel Blvd near Mahcester Rd, Vincent 15657 (MU); Bermof Rt I-70, N of Tylerville Rd exit, Cusick 24814 (MU). Columbiana Co., St Rt 170 at Dickinson Rd, SE of Petersburt, Cusick 36720 (MU). Crawford Co., Along the Pennsylvania railroad in Bresline, Bartley 2493 (NY). Cuyahoga Co., W side of Cuyahoga Riv. between Washington and Winslow streets, Cleveland, Cusick 24014 (MICH); Rt I-71 at exit to W 150^th^ St, city of Clevland, Cusick 24017 (MU). Fulton Co., berm of entrance ramp to Ohio Turnpike at St. Rt. 108, N of Wauseon, Cusick 25824 (MU). Guernsey Co., Berm of US Rt 22, SW jct of St Rt 513 at Antrim, Cusick 25806 (MU). Hancock Co., Ashland Oil Co. tank farm, S Sandusky St. and NE junction of Rt. I–75 and State Rt. 15 Findlay, Cusick 21882 (MICH). Hamilton Co., Junction of Lawrenceburg and Sand Run roads, Cusick 25551 (MU, NY); Weedy railroad yards, S of jucntion of Herring Way and Stadium Rd., city of Cincinnati, Cusick 26048 (KE, NY). Harrison Co., berm of US Rt. 22 at Nottingham Church, Cusic 25805 (MU). Higland Co., Jct of US Rt 50 and Cave Rd, W of Bainbridge, Cusick 35657 (MU). Jackson Co., City of Jackson, Pontious & Bartley 25 (NY). Lake Co., Entrance to Morton Salt Property, deadend of State Rt. 44, Grand Riv., Cusick 23967 (MICH); Rt I-90, ½ mi.E, jct of state route 306, city of Mentor, Cusick 23971 (MU). Lawrence Co., Junction of US Rt. 52 and Solida Rd., South Point, Cusick 24644 (MICH, NU, NY); 4 mi.SW of Hoxie, Taylor s.n. (BRIT). Lee Co., Along RR, 20^th^ St, Fort Madison, Cusick 35566 (MU). Lorain Co., Berm on S side of St Rt 2 at rest area, W of bridge over Vermilion Riv., SE of Vermilion, Cusick 29738 (CM); Lake Erie, E mouth of Black Riv., city of Lorain, Cusick 31314 (VPI). Lucas Co., Between Maumee Riv. and Ottawa St. at Anthony Wayne Bridge, Toledo, Cusick 27185 (CM); Ohio Turnpike, W of Exit No. 4, weedy ground at service plaza, Cusick 25823 (NY); Manhattan Blvd, at Bolevard Station, Toledo, Cusic 25906 (MU). Montgomery Co., Road berm at jct of Pinnacle and Vance Rds, city of Moraine, Cusick 25651 (MU). Muskingum Co., berm on S side of Rt I-70, 2 mi.E of St Rt 13 exit, W of Brownsville, Cusick 36964 (MU). Preble Co., Berm of US Rt 127 at bridge over Rt I-70, Cusick 24723 (MU); Berm of Rt I–70 exit ramp to US Rt 127, N of Eaton, Cusick 31362 (GH). Raleigh Co., Rt I–77 at service area, N of St. Rt 3 exit, NW of Beckley, Cusick 29859 (CM). Stark Co., RR crossing, Broadway Ave, S of state route 153, Louisville, Cusick 36972 (MU). Summit Co., N side State Rt. 18 directly W of junction of Rt. I–77, Montrose, Cusick 24019 (KE, MICH). Tuscarawas Co., RR crossing at St Rt 211 and 3^rd^ St, Dover, Cusick 35713 (MU). Warren Co., St Rt 123 at juct [of [Rt I-71, SE of Lebanon, Cusick 24813 (MU). Oklahoma. Alfalfa Co., Near head of Smith Cove, S side of Sal Plains Lake, Penfound 115 (OKL, OKLA); R10W, T27N, sec. 24, Ungar 1139 (KANU); 2 mi. N, 8-1/4 E of Cherokee, Stephens 71291 (KANU). Beaver Co., From SH 83 & 270 2.7 mi. E, Hoagland & McCarty 99-258 (OKL); Cimarron River, Smith 96 (OKL); Beaver Wildlife Mgmt Area (T4N R22E Sec 12), Hoagland & McCarty 2065 (OKL). Beckham Co., 3.6 mi. S of Carter under Hwy 34/Red River bridge, Buthod & Hoagland AB-10177 (OKL). Blaine Co., From 8a and 51a (W of Roman Nose SP) N ca. 1.7 mi, Hoagland & McCarty 99-7 (OKL); Watonga, 7 mi. N on Hwy 51A from intersection of Hyws 51A and 8, then 2.5 mi. W, Hoagland 0118-92 (OKL). Bryan Co., Hwy 70 bridge across Lake Texoma, Taylor & Taylor 1891 (OKL); Lake Texoma Shore at Johnson Creek, just NE of H; Sand dune area 3 mi. S, 1 mi. E of Albany, Taylor & Taylor 1544B (OKL); across Lake Texoma from Okla. Univ. Biol. Sta., Anderson 321 (OKL); Near Denison Dam of Lake Texoma, Riggs s.n. (OKL [01-0072171]). Canadian Co., T10N, R7W, S11, N/2 of NE/4, T. Albers (landowner), McCarty 727 (BRIT) and McCarty 728 (OKL). Choctaw Co., ca. 4 mi. NE of Swink, Taylor s.n. (BRIT 18811). Cimarron Co., 3 mi. NE of Kenton at base of a canyon in the E end of Black Mesa, Talyor & Taylor 25082 (BRIT); Near Lake Etling in Black Mesa State Park, Taylor & Taylor 2415 (OKL); Rocky canyon that runs to the W from Lake Carl Etling in the State Park, Taylor 16820 (OKL). Cleveland Co., 2 mi. N of Norman, Hopkins 767 (OKL); Canadian Riv. bed, Ten Mile Flats, Lawson and Goodman 578 (NCU, OKL); sandy bed of Canadian Riv., 3 mi. S of Norman, Goodman 4506 (NY, OKL). Comanche Co., Quanah Parker Lake, S end of island Wichita Mts. Wildlife Refuge, McMurry 835 (US); Geronimo Hill, Ft Sill Reservation, Thompson S0873 et al. (BRIT, OKL, OKLA); ibid, Thompson & Rudman S0601 (OKL, OKLA). Cotton Co., White’s Lake, 3 mi. N on Hwy 281/277 from intersection with 5A and 7 mi. W, Hoagland & McCarty 2111 (OKL); Randlett, 3 mi. S and 3 m. E on sectionline roads from intersection of Hwy 70 and Hwy 277/281, Hoagland & McCarty BLM0388 (OKL). Creek Co., Keystone WMA, 3.7 mi.W of 48/51 jct on Hwy 51, 2.0 mi. N to WMA, & 1.0 mi. E, T19N R8E Sec. 14, Hoagland et al., KEY-279 (OKL). Custer Co., Washita National Wildlife Refuge, T13N R19W Sec. 5, Buthod & Hoagland AB-3355 (OKL). Ellis Co., Four Canyon Nature Preserve, Hoagland 4C-430 & Buthod (BRIT) and 4C-431 (BRIT, OKL); Lake Lloyd Vincent, T9N R26W S34, McCarty 518 (OKL). Garfield Co., Near S end of Runway 35R, Johnson & Brown VAN0159 (OKL). Garvin Co., 1.5 mi.SE of Paul’s Valley, Pohl 5205 (SMU); T3N R3W Sec 20 SE/1/4, Crawford & Crawford 1355 (OKL). Grady Co., 6 mi. NW of Chickasha, Engelman s.n. (OKL [01-0072208]); 3 mi. NW of Minco along the South Canadian River, Pearce 1259 (OKL, OKLA, SMU); Alex Marsh, Anderson Farm, 1 mi. W on SH 19 & 1.2 N of Alex, Magrath et al. 19985 (OKL). Grant Co., 3 mi. W of Jefferson, Hoagland & McCarty 1769 (OKL). Greer Co., 1 mi.E of Plainview, Higgins 7678 (ASU, NY); 1.5 mi. S & 6.0 mi. W of Jester on EW Rd 160, Buthod et al. AB-3354 (OKL). Harper Co., From SH 64 & 34 6.0 mi. W and 6.8 mi. N, Hoagland & McCarty 99-238 (OKL); bridge over West Sandy Creek on US Rt 160, 6 mi. W of Attica, Vincent 10104 (MU); Alkali bottoms N of Laverne on Beaver River, Engleman s.n. (OKLA); Plainview, 5 mi. W along US Hwy 64, Freeman 18237 & Morse (KANU, OKL); Along the Beaver (Canadian) River, 15 mi. W and 4 mi. S of Buffalo, Taylor & Taylor 32695 (BRIT, OKL). Jackson Co., N boundary, 200 m E of active runway, Johnson & Proctor ALT0046 (OKL); 0.5 km NE of calibration hardstand, T2N R20W Sec 11, Johnson & Browning ALT0127 (OKL). Jefferson Co.Waurika, S side of Hwy 79 bridge at the Red River crossing, Hoagland et al. BLM464 (OKL). Johnston Co., ca. 4 mi. SE of Tishomingo on the Tishomingo Nat. Wildl. Refuge, Taylor & Taylor 4153 (BRIT); ibid, ca. 1 mi.NW of Reeves Lake, Thieret 41160 (MU). Kay Co., 6 mi. N. of Newkirk, Stephens 71090 (KANU); Blackwell, city park, Buthod & Hoagland AB4202 (OKL). Kingfisher Co., R9W T19N, Sec. 22, 1 mi. SE on Hwy 51 from bridge over Cimarron, Palmer 1647 (OKLA); 4.6 mi. NW of Kingfisher at Lake Elmer, Buthod & Hoagland AB-2509 (OKL). Kiowa Co., Just outside entrance to Quartz State Park, Smith 298 (OKL). Logan Co., N of Crescent on Hwy 74, Buthod et al. AB-4405 (OKL). Love Co., Edge of a branch of Lake Texoma called Wilson Creek, ca. 10.7 mi. NW of Marietta, Taylor & Taylor 26010 (BRIT). Marshall Co., Fobb Bottom State Hunting Area of Willis Island, Perkins & Perkins 585 (OKL); Margin of Lake Texhoma, Univ. of Oklahoma Biol. Station, Goodman 5758 (GH, KANU, OKL, SMU, UC); E side of Lake Texoma in Fobb Bottom Unit of Lake Texoma Public Hunting Area, Tyrl & Estes 65 (OKL); sandy margin of Lake Texhoma, OK. Univ. Biol. Station, Willis, Schaefer 386 (KSC). McClain Co., Wet sandy river bank, in bottomlands, just outside Purcell, Hopkins 635 (GH, OKL). Muskogee Co., 3.5 mi. SW of Ft. Gibson, Wallis 5471 (OKL, OKLA, SMU). Nowata Co., 3 mi. E from intersection of Hwys 169 and 60, 1 mi. S and 0.5 mi. E., Oolagah Wildlife Mgmt Area, Hoagland et al. OOL663 (OKL). Okfuskee Co., Near Okemah, 0.7 mi. W on Hwy 56 from junction with Hwy 48, Hoagland & Shannon 2692 (OKL). Oklahoma Co., North Canadian River below the dam of Lake Overholser, Taylor 22918 (BRIT). Okmulgee Co., Deep Fork Unit, Eufaula WMA, Benesh et al. DF0156 (OKL). Osage Co., Oklahoma R8E T28N S 25 SE ¼, Palmer 2286 (OKLA); S of Pawhuska, Engelman 1713 (OKL); South of Pawhuska, Engleman 300 (OKL). Payne Co., Sandy flood plain of Cimarron Riv., 1 mi.N of Coyle, Hutchinson 54 (NCU); 2 mi.N of Stillwater, Hendrix 21 (OKLA); Ripley, Learn 69 (OKLA, SMU); 2 mi. W of Stillwater, Ward 164 (OKLA). Pontotoc Co., Pontotoc Ridge Preserve, Folley & Johnson PON0432 (OKL). Roger Mills Co., Southwest floodplain, Washinta Battlefield National Historic Site, Buthod & Hoagland WAS-134 (OKL). Sebastian Co., Arkansas River floodplain, Sec 25, T8N, R31W, Thompson C1074 et al. (BRIT, OKL). Sequoyah Co., Arkansas River, 1 mi. SW of Gore on US Hwy 64, Wallis 1830 (OKL, OKLA). Texas Co., 2 mi. NW of Hardesty, Stephens 79407 (KANU); Guymon, 3 mi. E on Hwy 3 from intersections of Hwys 3 and 136, Hoagland 1140 (OKL). Tillman Co., 4.5 mi. E on Hwy 5C, from intersection with Hwy 5, then 2 mi. N and 0.5 E on section line arods, McCarty m9.137 et al. (BRIT); Hackberry Flat, Hoagland & Wagoner HF0135 (OKL). Tulsa Co., Juniper Hills Nursery, 1-1/5 mi. W of Jct US Hwy 64 & 121 St., Alleman 446 (OKLA). Washington Co., 2 mi. E and 1.2 mi. N of the S jct of US Hwy 60 and US Hwy 70, McDonald 991 (OKLA). Washita Co., 4 mi. W of Dill City, Johnson et al. e455 (OKL); 4.0 mi. S & ca. 3.0 mi. W of Burns Flat, OK, on Hwy 152, Hoagland et al. AB-2728 (OKL); ca. 4.4 mi. NE of Canute at Clinton Lake, Buthod & Hoagland AB-3352 (OKL). Woods Co., Near Arvard, Stevens 1698 (GH, MO, NY, OKL, OKLA); NW of Alva ca. 8 mi., Nighswonger 887 (NCU, OKL, SMU); ca. 9 mi. NW of Alva, Nighswonger 3410 (KSC, OKL); Ca. 9 mi. NW of Alva, Nighswonger 3849 (OKL, OKLA); Ca. 7 mi. NW of Alva, Nighswonger 4010 (OKL). Woodward Co., From SH 51 & US 281 N 6.0 mi. to blacktop, W 6.5 mi, Hoagland & McCarty 99-200 (OKL). Oregon: Malheur Co., Wet alkaline areas near Ontario, Bailey s.n. (GH). Pennsylvania: Allegheny Co., low ground between railroad tracks, 31st St. bridge, N of Smallman St., city of Pittsburgh, Cusick 25780 (CM, MICH); Between RR and Freeport Rd, 0.25 mi. N of Aspinwall, Cusick 36604 (MU). Bedford Co., berm of Penn. Turnpike just W of Exit 11, NW of Bedford, Cusick 26725 (CM); margin of PA turnpike, ¼ mi.W, exit to US Rt 220, N of city of Bedford, Cusick 37486 (MU). Berks Co., S side of Rt-178, Exit 19, St Rt 183, N of Strausstown, Cusick 37936 (MU). Cumberland Co., road berm, S side of Penn. Turnpike, just W of Exit 16, NE of Carlisle, Cusick 27798 (CM). Erie Co., 2 mi. NNE US Rte 20 exit on Rt I–90, Cusick 29147 (CM); E side of St. Rt. 18, just N of junction of Rt. I–90, Cusick 23991 (CM); Rt I-90 E at exit 32, Station Rd, SE of Wesleyville, Cusick 38241 (MU). Franklin Co., road berm, S side of Penn. Turnpike at Exit 14 ramp, 1.5 mi. SE of Willow Hill, Cusick 27800 (CM). Jefferson Co., Entrance ramp, St Rt 28 on N side of Rt I-80, NE of Brookville, Cusick 37896 (MU). Lawrence Co., Edge of US Rt 422 at Moravia Rd exit, S of New Castle, Cusick 36721 (MU). Lehigh Co., US Rt 22 at Krocks Rd, SW of Wescosville, Cusick 37943 (MU). Lucerne Co., St Rt 309, just S, jct of Rt I-80, N of village of Honey Hole, Cusick 38210 (MU). Monroe Co., St Rt 611 at entrance to Rt I-80, town of Delaware Water Gap, Cusick 38532 (MU). Northumberland Co., RR crossing, jct of state Rts 144 & 405, Watsontown, Cusick 37031 (MU). Somerset Co., Berm of Penn. Turnpike at tollgate, Somerset exit, Cusick 26095 (CM, MICH); RR crossing, Stoystown Rd, N of Pleasant Ave, city of Somerset, Cusick 37089 (MU). Washington Co., Berm of US Rt. 40 at junction of St Rt. 231 at Claysville, Cusick 25725 (CM). Westmoreland Co., Berm of Penn. Turnpike at tollgate, St. Rt. 31 exit, Donegal, Cusick 26094 (CM). Rhode Island. Kent Co., East Greenwich, Black Island, Congdon s.n. (POM); Warwick, Guttenberg s.n. (CM 352504); Warwick, Congdon s.n. (KSC). Newport Co., [...] Crescent Beach, Block Island, Fernald et al. 8765 (US). South Carolina. Beaufort Co., Sea Islands, Cuthbert s.n. (FLAS); St. Helena, Cuthbert s.n. (FLAS 10298). Berkeley Co. Santee Wool Combing Mill, Jameston on SC Rt 45, Ahles & Haesloop 25886 (NCU, RSA); ibid., Ahles & Haesloop 30831 (NCU); ibid., Ahles and Haesloop 47043 (NCU). Horry Co., Myrtle Beach, Thorne & Muenscher 8938 (RSA). Georgetown Co., Cat Island, edge of Winyah Bay at Lower Goose Pasture, just W of Rockfish Canal, Nelson 9379 (GA, USCH); Tom Yawkey Wildlife Center, marshy area just behind maritime forest off E side of Beach Rd., near its terminus on South Island, Nelson 9665 (USCH). South Dakota: Beadle Co., Huron, Williams s.n. (MO 2972215); T113N, R61W, Sec. 25, NE-1/4, Larson 9515 (RM); Iroquois, railroad ditches, Thornber s.n. (ARIZ 147347). Brown Co., Dry ponds, Aberdeen, Griffiths 111 (UC); T12N, R61W, Sec. 15, NW ¼, Fairlee 206 & Sieverding (KANU). Butte Co., T9N, R1E, Sec. 15, NW 1/4, Larson 8286 (RM). Kingsbury Co., Iroqouis, Thornber s.n. (GH, UC 120943). Lake Co., 3 mi.W of Madison, Garson 7007 (KANU). Marshall CO., T12N, R59W, Sec. 18, W ½ of NW ¼, Larson 11742 & Fairlee (KANU). Mellette Co., 1 mi. W of Kary, Tolstead 4–481 (GH, UC). Spink Co., Vicinity of Redfield, Ricksecker 209 (MONTU, MU, POM, UC); T115N, R65W, Sect. 18, NE-1/4, Larson 9606 (RM). Tennessee. Lake Co., Reelfoot Lake, off Hwy 78, Rogers 33668 (APSC). Shelby Co., Memphis railroad yard, Rogers & Bowers 8183 (NCU). Texas. Andrews Co., At waters edge and in low wet places in SE part of country, Scudday s.n. (LL). Aransas Co., NE of Fulton Beach, Correll & Correll 18953 (GH, LL, MO, NCU, SMU, UC); 3 mi. NE of Rockport, Jones 899 (SMU). Archer Co., Near the town of Holliday, Clark 355 (OKL). Armstrong Co., 0.5 mi.E of Washburn on Hwy 287, Whitehouse 17247 (MICH, SMU). Atascosa Co., Poteet, Silveus 145 (LL, TAES, TEX). Bailey Co., Lower Goose Lake, Muleshoe Nat. Wildlife Refuge, 5–1/2 mi. S of Needmore, Crutchfield & Evans 385–B (LL); 20 mi. S of Muleshoe, Stephens 73108 (KANU). Bastrop Co., Ab loco, Duval 393 (TEX). Bexar Co., San Antonio, Clemens & Clemens 454 (MO). Brewster Co., Ranger Canyon, W of Alpine, Steiger 1588 (NY); Big Bend N.P., Chisos Mts., Boot Spring Canyon, Warnock 12398 (TAES); Chisos Mts., Boot Ck., Parks & Cory 30353 (TAES); 9 mi. N of Terlugua, Sperry T–1265 (TAES); Devil’s Rio, W of Del Rio, Silveus 2377 (TAES); Bullis Gap Ranch, SW of Bullis Range, ca. 25 mi.S of Sanderson, Butterwick & Lott 3691 (LL); 20 mi. tank, S of Marathon on road to Big Bend N.P., Warnock 9976–A (LL, SMU); Boot Canyon, Correll 13723 (SMU); Stream above Boot Springs, Chisos Mts., Warnock 9744 (LL, SMU); Upper Cattail Falls, Chisos Mts., Warnock 20231 (TEX); Alpine, on golf course, creek bottom, Warnock 20059 (TEX); Along Hwy between Alpine and Ft. Davis, Teague T43 (LL, SMU). Brisco Co., 1 mi. E of Silverton, Stephens 72245 (KANU). Brooks Co., SW part of county on Rd. no. 755, Johnston 541463 (TEX). Burnet Co., ca. 200 ft W of Co. Rd. 122, 0.3 mi. N of RM 1431, Carr et al. 9142 (TEX). Cameron Co., El Jardin in the bottom of Resaca del la Rancho Viejo, Hitchcock 93 (TEX). Carson Co., 12 mi. W of Panhandle, Stephens 81545 (KANU). Castro Co., 12 mi. S of Dimmit, Johnston & Walker 6888 (LL). Chambers Co., 0.5 mi. NE of Lake Robinson, Hartman & Price 1094 (TAES). Coleman Co., Farm Rd 503, 11 mi.S of Voss, Nixon 238 (BRIT). Culberson Co., 5 mi. W of Kent, Warnock 9271 (TEX); S McKittrick Canyon, Guadalupe Mts., Warnock 10912 (SMU, TEX). Deaf Smith Co., Tierra Blanca Ck., south of Dawn, Waller 1169 (ASU, SMU); 5 mi. S of Glenrio, Waller 1244 (ASU, SMU). El Paso Co., Three Sisters Hills, ca. 1.5 air mi. N of jct. I–10 with N. Mesa, Worthington 17425 (TAES); El Paso, Hitchcock 1274 (GH, NY, P, TEX, UC, W); Hueco Tanks, 30 mi. NE of El Paso, Warnock 5834 (TEX); Hueco Mountains, Hueco Ttanks, Waterfall 6616 (SMU); El Paso, M.E. Jones 4203 (ARIZ, MU, P); Hueco Mts., Hueco Tanks, Worthington 5248 (TEX); ca. 3 mi. E of Jess Harris Road along Wingo Reserve Road, Gutierrez 1266 (ASU, UTEP). Floyd Co., 4.5 mi. S of Lockney, Stephens 80442 (KANU). Guadalupe Co., Seguin, Kellogg 47255 (TAES, TEX). Hansford Co., 4 mi. S., 8.5 mi. W of Gruver, Stephens 82401 (KANU); N of Spearman ca. 14 mi. along Palo Duro Creek, Higgins 12332 (ASU). Harris Co., Ab. loco, Geib 12 (TAES). Hemphill Co., 1–3/4 mi.NE of Canadian, Cory 35300 (TAES). Hartley Co., 8 mi.E of Hartley, Stephens 82115 (KANU). Hockley Co., 7 mi.N of Levelland, Stephens 72981 (KANU). Hidalgo Co., Jct of Hwy 281 and Hy 336 on the NW corner, Jones & Jones 865 (ARIZ). Hudspeth Co., Red Light Mills near the Red Light Draw road just E of the Quitman Mts., Butterwick & Lamb 2811 (TEX). Along Hwy 2 mi. E of Sierra Blanca, Warnock 13559 (TEX). Hutchinson Co., Near Fritch along Hwy 136, Higgins 9661 (ASU, NY). Jeff Davis Co., Fern Canyon, Davis Mts., Cory 9612 (GH); Madera Springs, Parks & Cory 17528 (TAES); Madre Springs, Cory 17528 (GH). Mt. Livermore, Hincley 331 (NY, TEX); Little Aguja Canyon, Davis Mts., Warnock et al. 8146 (TEX); ca. 22 mi. W of Ft. Davis, Warnock 9311 (TEX); Limpia Canyon, Davis Mts., 1 mi. above Ft. Davis, Warnock 7952 (LL, SMU); 1 mi. W of MacDonald Observatory, Davis Mts., Turner 2966 (SMU, TEX); Limpia Ck., Sperry T1411 (TAES). Jefferson Co., J.D. Murphree Wildlife Mgmt. Area, SW of Port Arthur, Stutzenbaker 142 (TEX, UC); Sea Rim State Park, Fleetwood 12215 (SMU). Jim Wells Co., Along US Hwy 281, ca. 35 air mi. WSW of Corpus Cristi, ca. 9.0 mi. N of Premont, Snow 5896a (MO). Karnes Co., 1 mi. NW of Panna Maria, Johnson 1265 (TAES). Kinney Co., 1 mi. SSW of Ruckman School, Johnson 1282 (TAES); 9 mi. S of Spofford, Cory 16730 (GH). Kleberg Co., Padre Island Nat. Seashore, Lemke 2995 (TEX). Knox Co., N of Benjamin on the S Fork of the Wichita Riv. breaks, Higgins 6079 (NCU); Kingsville, along FM Rd. 1356, ca. ¼ mi.W of US Hwy 77, Snow & Evans 4391–a (GREE); 2 mi. N of Mundy, Sumanth 312 (SMU). Lee Co., Lake Somerville near picnic pavilion, ca. 3500 ft ENE Of park headquarters, Nails Creek Unit, Carr 6124 (BRIT). Llano Co., Enchanted Rock, Sandy Ck., Butterwick and Lamb 2946 (TEX); Enchanted Rock, Tharp 7641 (TEX). Lubbock Co., Near Lubbock, Silveus 218 (TEX). Lynn Co., US Hwy 87, 2 mi.S of Tahoka, Harvey 7539 (MONTU). Martin Co., 5 mi. SE of Stanton, Jordan s.n. (SMU, accession and barcode lacking). Mason Co., Mason Mt Wildl. Mgmt Area, Middle Pasture, 0.3 mi. SE of gate into Headquarters Pasture, Sanchez 4037 (BRIT). Menard Co., Landers Ranch, 11 mi.S of Menard, Landers 5228 (TAES). Mitchell Co., Sect 10, J.P. Smith Survey, Pohl 4580 (SMU). Newton Co., Off TX Hway 87, ca. 8 mi. S of Bleakwood, Allen 20409 et al. (BRIT). Nueces Co., Beach near Corpus Cristi, Wolff 2345 (SUM). Ochiltree Co., Perryton sewage disposal pond, Headlee 115 (TEX); 3.5 mi. S of Perryton, Stephens 71605 (KANU, NY); 5.5 mi. N of Perryton, Stephens 71581 (KANU); 14 mi. S, 9 mi. W of Perryton, Stephens 82494 (KANU). Orange Co., 1 mi. S of Bridge City, Gould 11990 (TAES). Pecos Co., Flowering Wells, N of Ft. Stockton, Warnock T465 (ARIZ, GH, SMU, TEX). Palo Pinto Co., George Morre Farm (sandy upland), Caddell s.n. (BRIT). Potter Co., 0.2 mi. E of Hwy 11, N of Imperial, Druse & Dale 203–42 (TAES); 27 mi. E of Amarillo, Tharp 4165 (TEX). Presidio Co., Near pool in Brocks Canyon, Hinckley 2996 (TAES); Sierra Tierra Vieja, W branch of ZH Canyon near Vieja Pass, Hinckley 1998 (NY, SMU, TEX). Randall Co., Palo Duro Canyon, ca. 2 mi. NE of Canyon off Interstate 87, Higgins 11419 (NY); Ca. 2 mi. SE of Canyon, Higgins 11553 (NY); Buffalo Lake Nat. Wildlife Refuge, ca. 13 mi. W of Canyon, Higgins 12542 (ASU, NY); Banks of Buffalo Lake NWF, 2 mi. S of Umbarger, Crutchfield 3572 (TEX); 2.5 mi. S of Umbarger, Buffalo Lake NWF, Stephens 73347 (KANU); Canyon, Palmer 14085a (B, MO); 5 mi. SW of Bushland, Cory 16478 (GH). Reagan Co., 21 mi. N of Big Lake, Bacon 479 (TAES); 2.5 mi. S of Big Lake, Swallen 32763 (TAES); 5.5 mi. SW of Brushland, Parks & Cory 16477 (TAES); Bishop Ranch, NW of Stiles, Cory 4919 (GH). Reeves Co., ca. 15 mi. S of Pecos towards saragosa, Warnock 10178 (LL, SMU). San Patricio Co., Aransas Pass, corner of Conn Harbor Rd. and Hwy 361, Jones & Jones 727 (TAES); Welder Wildlife Area, 9 mi. NE of Sinton, Box 58 (TAES); ibid., Section 31, Lot 10, ca. 0.3 mi. E of entrance gate on US Hwy 77, Snow 5921 (MO). Roberts Co., 29 mi. S of Perryton, Stephens 71772 (KANU). Sherman Co., 14 mi. E, 12 mi. S, 4.5 mi. E of Stratford, Stephens 82391 (KANU). Swisher Co., Kress, Shinners 21130 (TAES); 12 mi. SE of Tulia, Stephens 81216 (KANU); 5 mi. E of Tulia enar Lake Tuila, Whitehouse 9969 (SMU). Taylor Co., Lake Lytle near Abilene, Tolstead 7559 (MO, SMU, TAES). Terrell Co., 3 mi. E of Dryden, Warnock 11897 (LL, SMU). Travis Co., Shoal Ck., N of Greenlawn Parkway bridge, Austin, Carr and Price 9212 (TAES); Lake Austin flood plain, Tharp 5143 (TEX); Colorado Riv., Austin, Tharp 3085 (TEX). Val Verde Co., Devil’s Riv., W of Del Rio, Silveus 2377 (TEX); W of Del Rio, Devil’s Riv., Silveus 269 (TEX). Webb Co., State Hwy 359, Lobo Ck., 14 mi. E of Laredo, Ramirez et al. 8678 (OKLA, TEX); Laredo, Shinners 17223 (SMU); State Hwy 359, Lobo Creek, 14 mi. E of Laredo, Ramirez 8687 et al. (SMU). Wichita Co., S side of Wichita Falls, Shinners 15847 (SMU). Zapata Co., End of Texas FM 496, ca. 2.5 mi. W of Zapata, gravelly shores of Falcon Lake, Snow 5900 (MO); ca. 250 m W of end of Texas FM 496, ca. 2.5 mi. W of Zapata, mucky shores of Falcon Lake, Snow 5901 (MO); Falcon Lake State Park, mowed grassy area in camping area, Snow 5903 (MO). Utah. Cache Co., Shallow water in marsh 5 mi.W of Logan, Holmgren and Woolf 8261 (UC). Grand Co., 2 mi. due NNE of Moab, sand bars of the Colorado Riv., Welsh 16370 (NY); ca. 0.5 mi. W of Dewey Bridge, Colorado Riv. Canyon, ca. 30 mi. from Moab, Welsh et al. 9469 (NCU, NY). Juab Co., 18.5 km ESE of Lynndyl, Utah, along Hwy 132, Goodrich 21876 (NY). Salt Lake Co., Salt Lake City, W bank of Jordan Riv. at 2300 So., Arnow 3745 (MO). Utah Co., East shore of Utah Lake near mouth of Provo River, Harrison 9882 (ARIZ). Washington Co., St George, S of town along Virgin River Parkway, Higgins 23563 (MU). Virginia. Isle of Wight Co., Sand–beach of James Riv., W of old Fort Boykin, Fernald and Long 13529 (GH). Princess Anne Co., Inner border of brackish to fresh marsh along Back Bay, at E margin of Long Island, Fernald & Long 10522 (GH, MO); Tidal marsh by Cobham Bay, James Riv., NW of Chippokes, Fernald & Long 12540 (GH); [...] Bay, E of Munden, Fernald and Long 1083 (VPI); [...] Back Bay, E of Creeds, Fernald & Long 10884 (GH). Surry Co., Fish House Bay, Hog Island State Waterfowl Refuge, Ware 6024 (FLAS, NCU); Fresh to brackish tidal marshes, Hog Island, Fernald & Long 12541 (GH). Washington: Klickitat Co., Damp sandy Riv. shore at Bingen, Suksdorf 11939 (GH, K, MO, P, UC); Damp sandy Riv. shore at Bingen, Suksdorf 10296 (K, UC, US). Whitman Co., Sandy bank of Snake Riv., Wawawai, St. John 6734 (US). West Virginia: Mason Co., berm of St. Rt 2, 0.5–0.7 mi. SW, US Rt. 35 bridge at Henderson, Cusick 24591 (MICH, NCU, NY). Ohio Co., berm of US Rt 40 at E limits of Triadelphia, Cusick 25726 (CM, MICH, NCU, NY); Nearly barren berm of US Rt 40 at NE limits of Valley Grove, Cusick & Shelton 29794 (CM). Wisconsin: Dane Co., Madison, Lake Wingra, Vilas Beach, Iltis 28184 (NY). Milwaukee Co., In cinders, Menomonee Valley railroad yards at 16th St., Milwaukee, Shinners 2623 (GH, NY). Richland Co., Seminary Street sidewalk at the railroad tracks, Richland Center, Nee 3358 (UC). Wyoming: Crook Co., T55N, R60W, Sec. 4, SW ¼, Larson 8278 (KANU). Big Horn Co., Yellowland Wildlife Management Area, Heidel 2123 (MONTU); Goshen Co., Springer Reservoir, ca. 15 air mi. SSW of Torrington, Snow 6072 (GREE, KSP, MO); Springer Reservoir, ca. 1.5 air mi. S of Yoder, ca. 13.5 air mi. SSW of Torrington, Nelson 2498 (KANU, NY, RM); Hawk Springs Reservoir, ca. 6.5 air mi. SE of Hawk Springs, ca. 25 air mi.S of Torrington, Nelson 2522 (NY, RM). Platte Co., Glendo State Park, Glendo Reservoir, receding shorline of Whiskey Gulch, Snow 6082 (KSP, MO). **Venezuela**. Falcon: Coro, margen de charca entre el aeropuerto y las dunas, Wingfield 5867 (MO, TAES). **US Virgin Islands**. St. Croix, St. John, Ricksecker 306 (GH, MO, UC).

#### Diplachne fusca subsp. uninervia

Taxon classificationPlantaePoalesPoaceae

(J. Presl) P.M. Peterson & N. Snow. Ann. Bot. 109: 1327. 2010.

Figures 7 , 8

Megastachya uninervia J. Presl, Reliq. Haenk. 1: 283. 1830. Poa uninervia (J. Presl) Kunth, Enum. Pl. 1: 344. 1833. Eragrostis uninervia (J. Presl) Steud., Syn. Pl. Glumac. 1: 278. 1854. Brizopyrum uninervium (J. Presl) E. Fourn., Mex. Pl. 2: 121. 1886. Leptochloa uninervia (J. Presl) Hitchc. and Chase, Contr. U. S. Natl. Herb. 18(7): 383. 1917. Diplachne uninervia (J. Presl) Parodi, Revista Centro Estud. Agron. 18: 147. 1925. Leptochloa fusca (L.) Kunth subsp. uninervia (J. Presl) N. Snow, Novon 8: 79. 1998. Diplachne fusca (L.) Roem. & Schult. var. uninervia (J. Presl) P.M. Peterson & N. Snow, Phytoenuron 2012-72: 12.
Tridens virens Nees, Fl. Bras. Enum. Pl. 2: 476. 1829. **Type.** BRAZIL. Habitat in graminosis ad fluvim S. Francisci in provincia Bahiensi et Permambucana, ad Joazeiro et alibi”, Collector unknown, Nees Herb. (holotype: BR?, n.v.; isotypes: BAA, fragment ex US [BAA00002970]!, US, fragment ex M [01231703]!).
Diplachne verticillata Nees and Meyen, Nov. Actorum Acad. Caes. Leop.–Carol. German. Nat. Cur. 19: Sup. 1: 27. 1841; 159. 1843. Nom. Nud. Tridens verticillatus Meyen, Reise Erde 1: 408. 1834, nom. nud. Uralepis verticillata (Nees and Meyen) Steud., Syn. Pl. Glumac. 1: 248. 1854. **Type.** CHILE. Copiapo, Mar 1881, FJF Meyen s.n. (holotype: B†; isotype: US, fragment ex B [00513498]!).
Fetuca glyceroides Steud., Berberid. Amer. Austral. 56. 1857. Nom. nud. **Type.** PERU. In camp. Medicag. det. Tacna, Lechler 1574 (holotype: P [P00740200]!; isotypes: BAA, fragment (?) [BAA00003539]!; LE [LE00000734]!, GOET, n.v., P [P00740201, P00740202]!, US, fragment ex GOET [00513498]!, W [0032101]!).
Atropis carinata Griseb., Abh. Konigl. Ges. Wiss. Gottingen 24: 291. 1879. Diplachne carinata (Griseb.) Hack., Bol. Acad. Ci. (Córdoba) 16: 253. 1900. Puccinellia carinata (Griseb.) Ponert, Feddes Repert. 84(9–10): 739. 1974. **Type.** ARGENTINA. Jujuy: El Volcán, 12-13 May 1873, Lorentz & Hieronymus 741 (lectotype, here designated: GOET [GOET006599]!; isolectotypes: BAA, fragment ex CORD [BAA00001517]!, CORD [CORD00004678]! , S, fragment [S05-10049]!).
Leptochloa imbricata Thurb., Bot. California 2: 293. 1880. Diplachne imbricata (Thurb.) Scribn., Bull. Torrey Bot. Club, 10(1): 30. 1883. Rabdochloa imbricata (Thurb.) Kuntze, Révis. Gen. Pl. 2: 788. 1891. **Type.** United States of America. California. Southern part of San Diego County, Larken’s Station, 1875, E Palmer 404 (lectotype, here designated, NY [00019497]!; isolectotypes: GH [00062450]!, US, fragment ex GH [00386253]!).
Leptochloa virletii E. Fourn., Mex. Pl. 2: 147. 1886. Leptochloa virletii E. Fourn. ex Hemsl., Biol. Cent.-Amer., Bot. 3: 566. 1885. Nom. nud. **Type.** MEXICO. Prox de San Luis Potosí, Virlet 1404 (holotype: P; fragment of holotype, US [00478610]!; isotype: BAA [BAA00002178]!.
Diplachne uninervia var. typica fo. abbreviata Parodi, Revista Fac. Agron. Univ. Nac. La Plata 6: 36. 1927; Revista Fac. Agron. Veterin. 6: 18. 1927. **Type.** ARGENTINA. Buenos Aires, Pergamino, Basualdo, 13 Noviembre 1925, leg. LR Parodi 6703 (lectotype, here designated: BAA!). The specimen chosen for the lectotype was also cited by Parodi, Rev. Fac. Agron. Veterin. 6: 19. 1927.
Diplachne festuciformis H. Scholz, Willdenowia 11(1): 98. 1981. **Type.** LIBYA. C Ricceri & CH Steinberg 129 (holotype: FI!).

##### Type.

MEXICO. Haenke 101 (lectotype, designated by Snow [Novon 8: 79. 1998]: PR!; isolectotypes: LE, W [0028807]!, US, fragment [00513245]!).

##### Description.

Plants annual (or sometimes weakly perennial in regions with infrequent freezing). Culms (15–) 25–110 cm tall, 2.0–5.0 mm wide at base, round, erect, ascending, often branching; nodes glabrous; internodes 2–11 cm long, soft, hollow. Leaf sheaths longer or shorter than the internodes, often flattened below, glabrous on sides and margins; ligules 5–8 mm long; blades (2–)5–37 cm long, 2.0–5.5 mm wide, linear, usually densely scabrous above, sparsely to densely scabrous below. Panicles 10–57 ×(0.5–) 3–18 cm, branches (3–)10–60 and most or all exserted at maturity; branches (0.3–)2–11 cm long, mostly alternate along the rachis, ascending to erect, stiff or slightly flexuous, minutely scabrous, axils glabrous. Spikelets 5–10 mm long, rarely distant to normally imbricate (sometimes tightly so); florets (3–)6–10; callus sparsely sericeous; lower glumes 1.0–2.6 mm long, narrowly triangular, narrowly ovate, or ovate, glabrous or scabrous on midnerve, acute to aristate or mucronate; upper glumes 1.8–2.8 mm long, obovate to widely ovate, glabrous or scabrous on midnerve, obtuse, acute, or rarely mucronate; lemmas 2.0–3.6 mm long, ovate or elliptic, light brown to very dark green or somewhat plumbeous, lateral nerves more or less prominent and generally extending to edges, sparsely sericeous below on lateral nerves and often midnerve, glabrous between nerves, apex broadly acute, more commonly obtuse to truncate, sometimes bifid or mucronate; paleas subequal or slightly longer than lemma, elliptic, sericeous along nerves; apex obtuse. Stamens 3; anthers 0.4–1.0 mm long, yellow. Caryopses 1.0–1.5 mm long, 0.7–0.8 mm wide, elliptic, ovate, or obovate in hilar profile, transversely elliptic in transverse section, hilar groove lacking, smooth or slightly rugose, brown; pericarp weakly adnate to endosperm.

**Figure 7.**
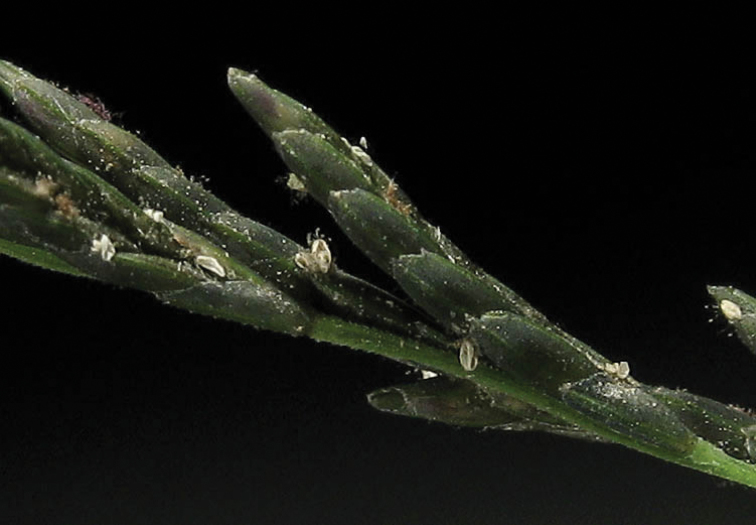
Spikelets of Diplachne fusca subspecies uninervia. The dark olive-green or often plumbeous color of spikelets is common for this subspecies, but rarely occurs in its Neotropical counterpart D. f. subsp. fascicularis. Photo with permission by Anthony Valois, Santa Monica Mountains National Recreation Area, California (no voucher).

**Figure 8.**
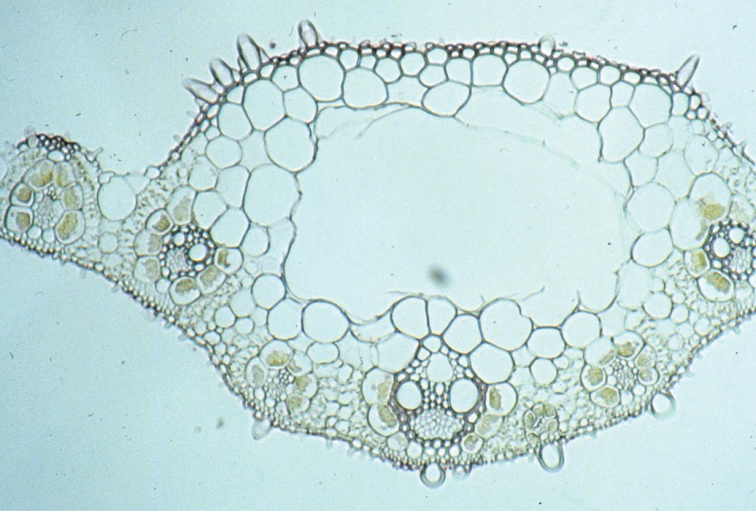
Cross section of leaf of Diplache fusca subsp. uninervia (Snow 5916-F) displaying nearly identical midrib anatomy as that of subspecies muelleri (see Fig. 4).

##### Leaf anatomy.

As reported above for D. fusca subsp. fascicularis. (Vouchers [at MO]: Snow 5916–F [USA]; Snow & Simon 7387 [Australia].)

##### Stem anatomy.

Internodes hollow. Inner sclerenchymatous ring present. Peripheral sclerenchymatous ring present. Peripheral sclerenchymatous girders connected to the outermost vascular bundles present or absent. Intervascular peripheral sclerenchymatous pillars not associated with outermost vascular bundles absent. Inner sclerenchymatous ring canal tissue absent. Kranz sheath cells present. Kranz sheath cell canal tissue absent or present. Vascular bundles nested in outer portion of Kranz sheath cell canals not applicable. Sclerenchymatous rings surrounding vascular bundles located inside inner sclerenchymatous ring absent. Sclerenchymatous rings (5–10 cells thick) surrounding outermost primary vascular bundles absent.

##### Chromosome number.

*n*=10 (Gould 1958); 2*n*=20 (Gould 1958, 1968).

##### Phenology.

Flowering throughout the year in both Northern and Southern hemispheres as limited by cold or moisture availability.

##### Distribution.

The native and non-native distributions of Diplachne fusca subsp. uninervia are uncertain, particularly regarding the southwestern USA west of Texas and east of the Mississippi River (but excluding Florida), where it is considered to be non-native based on relatively few older collections (early 20^th^ Century or earlier). In contrast, the occurrences north of Virginia are certainly all non-native. Given its greater abundance in parts of the USA, México and South America (e.g. Argentina, Paraguay), it is hypothesised that this taxon is an amphitropical disjunct, although it was not reported as such previously (Peterson et al. 2007). **Native**: In the New World mostly south of Latitude 37°N, south to Argentina, occasionally adventive in Old World; open mesic areas, agricultural lands, saline flats, mangrove swamps. Elevation from sea level to 1200 m (TDWG: 74: OKL; 77: TEX; 78: ARK, LOU, FLA; 79: MXC, MXE, MXG, MXN, MXS, MXT; 80: BLZ, COS, HON, NIC; 81: CUB, PUE; 82: VEN; 83: BOL, CLM, ECU, PER; 84: BZC, BZE, BZN, 85: AGE, AGS, AGW, PAR, URU). **Non-native**: Australia (Snow & Simon 1997), New Zealand, Jordan, Saudi Arabia, Spain (TDWG: 11: CZE-CZ; 12: SPA; 34: PAL-JO; 35: SAU; 50: NSW, NTA, QLD, SOA, TAS, WAU; 51: NZN; 63: HAW-HI; 71: BRC; 73: COL, ORE; 74: ILL, KAN, MSO, OKL; 75: MAI, NWJ; 76: ARI, CAL, NEV, UTA; 77: NWM. 78: ALA, GEO, MSI, NCA, SCA, TEN, VRG).

##### Conservation status.

Least Concern (IUCN 2012) given its wide distribution.

##### Etymology.

*Uninervia*, meaning one-nerved, may be a reference to the prominent nerve on the glumes.

##### Vernacular names.

Mexican sprangletop. Argentina: hierba paymilla, (Aguilar 1 [NY]). México: zacate salado mexicano; zacate gigante peruano. Venezuela: paja de ratón (Campos 519 [US]).

##### Comments.

Diplachne fusca subsp. uninervia has a greater tendency to become invasive and weedy compared to other taxa in the genus (Snow and Simon 1999; Perez et al. 2010).

The panicle of *D. f. subsp. uninervia* generally is completely exerted and (typically) narrowly elliptic to elliptic in profile. Some specimens from Arizona and California have highly contracted panicles with erect branches (Peebles & Harrison 5063 [US]; Lemmon & Lemmon 360 [UC]). However, most others have more open and diffuse panicles (Pedersen 4071 [M [MO]; Guaglianone et al. 358 [SI]; Snow 6598 [MEXU, MO]). The often truncate (or sub-truncate) and frequently mucronate apex of the lemma and smoky white glumes can make this subspecies appear similar to the southern African form some have called *D. cuspidata* (e.g. Geiss & Van der Walt 12632 [M, MO]; see Gibbs Russell et al. 1991), which the authors include as a synonym of *D. f. subsp. fusca*.

The report of *D. fusca* from the Canary Islands (Scholz and Böcker 1996) applies to *D. f. subsp. uninervia*.

Rusts can cause abnormal expansion of florets in South American specimens (Jörgensen 2445 [SI]; Krapovickas et al. 18507 [P, UC]; Hartley SH126 [US]; Stuckert 18787 [MO, NY, W]).

##### Specimens examined.

**Argentina**. Buenos Aires: Dpto. Fred G. Meyer, General Pacheco, en el bañado del río “Las Conchas”, Krapovickas 2723 (MO); Becca, Burkart 26443 (US); Retiro, Parodi 8102 (GH); Vicente López, Parodi 7693 (GH). Chaco: Dpto. Resistencia, entre Barranqueras e Isla Antequeras, Krapovickas & Cristóbal 12747 (SI, UC); Las Palmas, Jörgensen 2448 (GH, NY, US); Dpto. San Martín, Ea. La Leonor, Schinini 26276 (GH, MICH, MO). Córdoba: Salinas Grandes, Sánchez & Arriaga 1271 (MU). Dpto. A. M. Anton, La Francia, alrededores de la Estación YFP, Ariza & Astegiano 2502 (NY); Dpto. Cruz del Eje, Salinas Grandes, ruta 38, sobre el límite con La Rioja, Hunziker et al. 15876 (NY); Dpto. Río Primero, Estancia San Teodoro, Stuckert 14834 (MO) Dpto. Marcos Juárez, M. Juárez, Stuckert 14794 (MO, NY); Dpto. Justo, 13 km E de La Francia, Krapovickas et al. 18507 (P, UC). Corrientes: Dpto. Capital, Ruta 12, 11km W de Corrientes, camino a Sta. Ana, Shinini 16056 (US); Dpto. Itatí, Ramada Paso, Krapovickas and Quarín 20928 (NY); Dpto. Mburucuyá, Estancia “Santa Teresa”, Pedersen 1880 (GH, MO, NY, P, US); 50 km al N de Mercedes, Zuloaga et al. 3348 (MO); Dpto. San Cosme, Paso de la Patria, Nicora 8679 (MO); Dpto. San Miguel, 12 km S de Caa–Catí, Ahumada 1710 (MO); Dpto. Paso de los Libres, Parada Pucheta, Ruta Nac. no. 127, Ahumada 2547 (MO); Dpto. Saladas, Rio San Lorenzo, Schwarz 9408 (US); Dpto. Empedrado, Estancia “La Yela”, Pedersen 11671 (A, MO, NY). Entre Rios: Dpto. Federación, Río Mocoretá, ruta 14, Burkart & Gamerro 21599 (SI, US); Dpto. Gualeguaychú, Puerto Constanza, Burkart 21640 (SI); Dpto. La Paz, distrito Banderas, Estancia La Esperanza, Burkart & Bacigalupo 21041 (SI); Gualeguaychú, Troncoso and Bacigalupo 3231 (MU). Formosa: Dpto. Boca del Bermejo, Estancia “Herradura”, Pedersen 1237 (P, US); Dpto. Patiño, ca. 30 km SW Villa General Güemes, Chapin and Eskuche AC20319 (SI); Brazo N del río Pilcomayo, a la altura de Monte Lindo y en las Pampas, entre Puesto Whassich y Parada, Cordini 74 (GH, SI, US); Ruta 11 km 110, Morel 3676 (US); Ruta 11 vieja, al N de Ayo. Francesa Cué, Guaglianone et al. 264 (LP, SI); Dpto. Pirané, Pierotti 4138 (MO, OKL); Ab. loco, Jörgensen 2445 (SI); Ruta 11 vieja, al N de Ayo. Francesa Cué y S de Ea. La Emilia, Guaglianone 358 (SI). La Roja: Vinchina, Burkart 12229 (MU). Jujuy: Dpto. San Pedro, San Lucas, Cabrera et al. 27531 (SI). La Pampa: Dpto. Catrilo, Uriburu, Fortuna 9 (UC, US); Uriburu, Fortuna 55 (UC, US); 1 km N of Volcan and 6 km S of Tumbaya on Hwy 9, Peterson & Annable 10265 (K, MO, NY, RSA, US); Sierra Lihuel–Calel near campground in Park, along creek, Peterson & Annable 11237 (K, US). Mendoza: Dpto. Lasalle, Finca “La Holanda”, Dawson and Pujals 1459 (LP); Prov. de San Luís, Estación Jarilla, Leal 9134 (ARIZ); At Mendoza, Beetle 628 (UC); Sta. Rosa, Jensen–Haarup 1905 (US); Guaymallén–corralitos, Semper 309 (MICH). La Rioja: Dpto. Gral. Samadrid, Villa Castelli, Meyer 4112 (GH, UC). Dpto. Rosario Vera, Chelcos, Peñaloza, Stuckert 18787 (MO, NY, W); Chelcos, Peñaloza, Stuckert 18755 (NY); Dpto. San Martín, Ulapes, Stuckert 17072 (MO, NY); Dpto. Gral. Belgrano, alrededores del paraje Cortaderas, cerca de la Ruta Nac. no. 79, Biurrun & Pagliari 6313 (ARIZ). Neuquén: Capital, Parodi 2711 (BAA). Rio Negro: Vicinity of General Roca, Fischer 49 (GH, MO, NY); Isla Choele–Choel, Clos 3591 (BAA). Salta: Dpto. Ancash, fuera de pampa de grama, Aguilar 1 (NY); Rosario de la Frontera, Lillo 3901 (GH, NY, US, W). San Juan: Dpto. Calingasta, Calingasta, Fabrus & Marchionni 2398 (LP); at km 1137, 26.7 km SE of Molinos on Hwy 40, Peterson et al. 10178 (K, MO, US); Dpto. Calingasta, Agricultural road E of Hwy 412 and W of Rio Castano Viejo, 16 km N of Calingasta, 10 km S of Puchuzun, Peterson et al. 19255 (K, MO, US); Dpto. Iglesia, Villa Nueva, Castellanos 15395 (US); Dpto. Sarmiento, camino de Mendoza a San Juan, cerca de Media Agua, Nicora 4285 (SI); Dpto. Zonda, Sierra Alta de Zonda, km 29 del camino a Calingasta, Nicora et al. 8446 (NY, US). San Luís: Alto Pencoso, Baruch–Carette s.n. (NY); Between Chomes and Alto [P]encosa, Beetle 573 (SMU). Santa Fe: Río Salado, Job 1118 (NY, US); Río Salado, Isla Sivori, Job 619 (US); Dpto. Gral. Obligado, entre ruta 11 y Villa Guillermina, Pensiero & Vegetti 2585 (MO); Dpto. 9 de Julio, 2 km E de ruta 95, a 50 km N de Tostado, Pensiero & Vegetti 2735 (MO); Dpto. General Obligado, Pensiero and Vegetti 2611 (MO); Dpto. San Cristóbal, Esteban Rams, en tereno adjacente a la estacción, Krapovickas 657 (NY, SI). Santiago del Estero: Dpto. Banda, ab loco, Wahnish 2056 (SI); Dpto. Capital, Capital, Bianchi 35554 (GH); Dpto. Carlos Pellegrini, Rio Ureña, bañados del río, Venturi 5661 (SI); Dpto. Capital, alrededores cuidad Sao, Río Dulces, Ulibarri 1775 (SI); Dpto. Cope, San José de Boquerón, Luna 1239 (US); Dpto. Pellegrini, Rio Urueña, Venturi 5661 (NY, US); Dpto. Robles, Colonia Jaimez, Luna 1380 (MO); Dpto. de Silipic, Bartlett 20430 (US). Tucumán: Dpto. Burruyacu, Cerro del Campo, Venturi 7654 (SI); El Puestito, Venturi 7672 (MICH, SI); Dpto. Cruz Alta, orilla del rio Salí, Venturi 760 (GH, NY, US); Ab loco, Monetti 1028 (GH, NY). Dpto. Leales, Chañar Pozo, Venturi 439 (GH, LIL, SI, US); Dpto. Monteros, Acharal, orillas del río Arenilla, Venturi 2543 (SI, LIL, US); Dpto. Trancas, Tapia, Rodríguez 194 (BAF, LIL, NY, P, SI, US). **Australia**. New South Wales: Newington Naval Arms Depot, Homebush Bay, Jacobs 6546 (NSW). Northern Territory: Elparpa [= Ilparpa] Swamp, Latz 7607 (NSW); Palm Valley, 12 mi. SW of Hermannsburg, Mission, Lazarides 5290 (NSW). Queensland: Moreton Dist., Brisbane, Pinkenba, Blake 23075 (CANB, NSW); Indooropilly, Brown 243 (BRI, CANB [n.v.], MEL); North Kennedy Dist., Town of Bowen, in ditches along Roadside, Snow & Simon 7387 (BRI, MO); Awonga Dam, Iveragh Reach, 15 km SE of Calliope, Gibson TOI347 (BRI). South Australia: Barker Inlet South Wetland, Adelaide, Green 1988 (BRI); Water’s Bolivar Sewage Treatment Works, Bolivar and St. Kilda, Adelaide, Green 1993 (BRI). Tasmania: Woodbury, Black 1270.635 (MEL, US). Western Australia: 123 km S of Mt. Augustus station on Landor/Mt. Augustus Rd at Landor Ck, Peterson et al. 14375 (K, MO, NY, RSA, US); Tank near Milbillillie H/S, Craven 5383 (CANB, MO); Kimberely Research Stn., Kununurra, Parker 471 (BRI); Carawine Gorge, ca. 140 km SE of Shay Gap, Newbey 10463 (CANB); Corong Ck., Woodstock Stn., S of Port Hedland, Burbidge 5845 (CANB); Dept. of Agric. Exper. Farm, Kununurra, Gilbey s.n. (CANB). **Belize**. The Fort Belize, Smart 46 (US). **Bolivia**. Cochabamba: Prov. Cercado, City of Cochabamba, the NW shore of Laguna Alalay, Ritter 745 (NHA); Cochabamba, Steinbach 8746 (MO, NY); San Pedro, San Pedro, Sahonero 19 (MO); LA PAZ: Colcapirhua (Cochabamba) a 7 kms, Adolfo 155 (US); Punata–Cochabamba, Spiaggi 39 (US). **Brazil**. Amapá: Rio Araguarí, entre Apurema e Uruguaina, Black & Froes 51–12376 (US); São Joaquim, Black & Lobato 50–9399 (US). Mato Grosso do Sul: Faz. Bodoquena, Carandazal, Mun. de Corumbá, Allem et al. 2197 (MO). Pará: Fazenda Camburupy, near Soure, Isla de Marajó, Swallen 4926 (US); Marajó Island, Estate “Gavinho”, Goeldi 173 (KSC, NY, US, W). Rio Grande do Norte: Angicos, Swallen 4704 (BAA). Canary Islands. Gran Canaria, Wasserstellen im Barranco de Maspalomas nähe der Meeresmündung bei El Oasis, Scholz s.n. (B). **Chile**. Atacama: Pica, Pérez 20596 (US). Tacna: Tacna, Werdermann 735 (BAA, GH, LIL, MO, NY, UC, US); desert of Arica, Skottsberg & Skottsberg 1103 (NY). Tatrapacá: Ab loco, Phillippi 3 (US) and Philippi 1888 (W); Arica, Jaffuel 1606 (BAA); Valle de Lluta, Arica, Pfister 9488 (US); Iquique, Barros 716 (BAA); [collected from] ca. 1/2 mi.S of Victoria, Salar del Sul, Norte Grande [raised from seed at Environm. Res. Lab, Tucson], Yensen NPY800428-16 (ARIZ). **China**. Hong Kong: Castle Peak, China Light Ash Lagoon, Hu & But 22508 (A); Hong Kong, Castle Peak, Power Station, Hu & But 22229 (A, MO). **Colombia**. Atlántico: Entre Palmar de Varela y Ponedera, Dugand 5304 (COL, US). Magdalena: Ciénega, alrededores de Aguacoca, Romero–Castañeda 7268 (COL, US). **Costa Rica.** Guanacaste: ca. 1 km E of Rio Tempisque ferry, Crow 6110 (MO, RSA). **Cuba**. Oriente: Near Novaliches Stn, S of Guantánamo, Hioram 1359 (US); Estación de Novaliche, Guantánamo, Hioram 2166 (GH, NY, US). Diego Garcia: Grass in coral sand quarry east of runway, Whistler 9775 (US); in clearing, Field 112 (K). **Ecuador**. Guayas: Guayaquil, near the cement mill, Asplund 7695 (NY, P); Guayaquil, wet clayey ground, Asplund 15991 (NY). **Egypt**. 166 km from Cairo on the desert road to Alexandria, Amin et al. s.n. (H, K, MO); Rice cultivation at Mut, Dakhla Oasis, El Hadidi 623 (K, MO). **Honduras**. Choluteca: 2 km NO San Bernardo, Repulski 503 (MO). **Jamaica**. Hills W of Great salt Ponds, Orcutt 6470 (UC); Port Henderson, Ridley 132 (US); Salt ponds, Harris 12309 (BISH, NY). **Jordan**. Wadi Araba, 50 km S of Ghor Safi, 5–8 km E of the main road to Aqaba, along the lane to Ammarien and Sadien tribes farm, Al–Eisawi 2429 (BM). **México**. Baja California Norte: Mpio. de Ensenada, 3 km N of Maneadero, Moran 24517 (ENCB); Adobe flat in La Mesa, SE of Tiujuana, Moran 18568 (ENCB); Mpio. de Mexicali, ca. 0.3 km S from Prese Morelos, Felger 06-17 et al. (ARIZ); Mpio. de Mexicali, Lower Río Colorado valley, adjacent to irrigated agriculture, Felger 06-38 et al. (ARIZ); Along draw, Chase 5518 (US); Canyon de Guadalupe, Thorne et al. 61679 (RSA); Punta Banda, SW of Ensenada, Beetle M–2825 (RSA) Entrance to San Carlos Canyon S of Ensenada, Beetle & Alcaraz M-6598 (ARIZ). Baja California Sur: Banks of Rio Colorado below Yuma, MacDougal s.n. (NY); Río Comondú, along road to Comondú, 14.25 road mi. NE of E. Francisco Villa, Baker 8740 & Johnson (ARIZ); La Paz, Palmer 134 (GH, NY, UC); El Centenario, 10 km al SW de La Paz, Domínguez L. 381 (ARIZ); Sandy beach near El Centenario, W of La Paz, Reeder & Reeder 6732 (ARIZ, ENCB); Quitovac between Sonoyta & Caborca, Nabhan 161 (ARIZ); Villa Ignacio Zaragosa, ca. 10 km NW of Ciudad Insurgentes, Snow 6484 & Prinzie (MEXU, MO); N of Villa Constitucion, 20 km E of Insurgentes, Beetle M–2477 (MO); Arroyo de San Raymondo, 78 km NW of La Puríssima, Carter et al. 2501 (MEXU, UC, US); San José del Cabo, Purpus 312 (MO, UC, US); Vicinity of San Jose del Cabo, Wiggins 5685B (US). Chiapas: Mpio. Tonalá, E shor of Mar Muerto, N of Paredón, Breedlove & Thorne 20834 (DS, ENCB, MEXU, MICH, MO, NY, RSA); Mpio. Tonalá, NW of Puerto Arista, mangroves behind sand dunes, Breedlove & Davidse 54224 (CAS, MO, NY); Mpio. of Tonalá, mangroves behind sand dunes NW of Puerto Arista, Breedlove 52842 (CAS, MO). Chihuahua: Cerca del rio, Pringle 438 (MEXU). Guanajuato: Irapuato, Hitchcock 7432 (US). Guerrero: Coyuca, Hinton et al. 5543 (MEXU, MO, NY, US). Jalisco: 2 km al N de El Palo Blanco, ejido el Limón, Santana M. 8283 et al. (BRIT); 8 km al S de Acatlán de Juárez, Rzedowski 14516 (ENCB); Rio Blanco, Palmer 331 (GH, MO, NY, US); 9 mi.N of Zocoalco, by the road towards Acatlan, Dorado et al. 1676b (RSA); Laguna de Zacoalco, Díaz Luna 1063 (ENCB, RSA); Lagoon SW of Guadalajara, 20 km despues de Calera, Beetle M-5313 & Guzman M (ARIZ). Morelos: Mpio. Tlaquiltenango, Lagunillas a la altura del Terraplen, Vasquez 660 (ARIZ, NY). Nayarit: Matachen, beyond the point S of San Blas, McVaugh 19450 (MICH, n.v., US). Oaxaca: N of Tuxtepec, Nelson 367 (US). Sinaloa: 14.9 road mi. S of border of Sonora along Hwy 15, Snow 6527 & Prinzie (KSP, MO, MU); Headwaters of the Mazatlan Riv., Wright 1317a (US); Vicinity of Mazatlán, about a salt marsh, Rose et al. 14108 (NY, US). Puebla: Mpio. de Tehuacán, Tehuacán, NW side of town near the highway to Puebla, Steinmann & Steinmann 2401 (ARIZ). México: 10.5 mi.N of Aculco on Hwy 55 towards San Juan Del Rio, Peterson 21305, Saarela & Flores Villegas 21305 (US). Sonora: Sonoyta, northwest side of town ca. 0.5 mi. south of river, Felger 86-402A & Joseph (ARIZ); Wuitovac between Sonoyta aand Caborca, Nabhan 161 (ARIZ); Presa Derivadora, on Río Santo at NE side of town of Sonohyta, Felger 86-297 & Leigh (ARIZ); ca. 3.5 km E of village of Tastiota, Bunnell et al. 20891 (ARIZ); 2.3 mi. on Sonora Hwy 24 N of El Sahuaral, Felger 86-27 & Straub (ARIZ); Mpio. de Pitiquito: Pozo Coyote, small ranch ca. 10 km northwards from El Desemboque along the Arroyo San Ignacio, Felger 83-104 et al. (ARIZ); 3 km E of Puerto Peñasco, empty lot between beach houses, Felger 85-800 (ARIZ); ca. 10 mi. W of Sonoyta on Mex Hwy 2, Felger 20594 et al. (ARIZ); Río Sonoyta, ca. 1.6 km SSW of Quitobaquito, Felger 89-34 & Broyles (ARIZ); Río Sonoyta 21 km on Mex 2 W of Sonoyta, at ca. 1 km W of Quitobaquito and ca. 1 km S of highway, Felger 85-972 & Van Houten (ARIZ); Río Sonoyta at Sonoyta, Felger 85-708 & Dimmitt (ARIZ); Rio Magdalena at Tumutama road in Magdalena, Reina G. 2001-602 et al. (ARIZ); Cienega de Santa Clara, delta region of Rio Colorado, 7.7 mi. along canal road SW of El Golfo-San Luis Hwy, Felger 92-524 et al. (ARIZ); 18 km S of San Luis Río Colorado on road to El Golfo, Felger 85-1042 & Van Houten (ARIZ); San Luis Río Colorado, W side of the city, on the east bank of the Río Colorado, Felger 93-204 & Ortiz Reyna (ARIZ); ca. 2 km NW of Condominios Pilar at ca. 0.5 km W of Estero Soldado, vicinity of Bahía San Carlos, Felger 84-522 (ARIZ); Intersection of Hwys 15 and 128, ca. 25 road mi. W of Navojoa, Snow 6598 (MEXU, MO); Mpio. de Yecora, El Llano de Curea, Reina 2004-539 (ARIZ); 2 mi. NW of Sahural, between Guaymas and Kino Bay, Spellenberg 4603 (NMC, NY); Yaqui Riv., Palmer 5 (MO, US); SE of Guaymas, along Hwy 15, ca. 100 m S of km 82 signpost, Snow 6567 & Prinzie (MEXU, MO); Along Hwy 14 leading NE out of Hermosillo, ca. 37.5 km SW of Ures, Snow 6568 & Prinzie (MEXU, MO); Hermosillo, Hitchcock 3577 (US); 2.3 mi. on Son. Hwy 24 N of El Sahuaral, Felger and Straub 86–27 (MICH). Tamaulipas: Matamoros, km 25 al S de la playa Lauro Villar, Baro 253 (MO); Mpio. Valle Hermoso, “Distrito 025”, Mora–Olivo 5182 (MO); Mpio. González Manuel, Villega–López 271 (MO). Veracruz: Near Ebano, banks of Panuco, LeSueur 662 (ARIZ); Mpio. Panuco, Laguna de Tamos, sobre la carretera Tampico–Panuco, Calzada & Marquez 4503 (ARIZ, ENCB). **New Zealand**. Waikato: Thames, Walker 25084 (US). **Nicaragua**. Managua: S shore of Lago de Managua, ca. km 31 on Carretera Nueva a Leon, near Piedras Azules, Stevens 13157 (CM, MO). **Paraguay**. Boqueron: Estancia Primavera, Ramírez 25 (BAA); Isla Poi, Col. Menno, Laguna Captian, Vanni et al. 2036 (MO); Col. Fernheim, Ea. Laguna Porá, Vanni et al. 2580 (GH, MO); Ruta Trans Chaco, en préstamos al lado del camino con aqua semi–permanente, Schinini & Palacios 25800 (MO); Puerto Casado, Rojas 2305 (US); Puerto Casado, Rojas 8783 (US); FC Casado km 10, Rosengurtt B–5852 (K, SI); Puerto Casado, Hartley SH126 (US); Puerto Casado and vicinity, near Estancia “Guajhó”, Pedersen 4071 (MO). Chaco: Loma Clavel, Hassler 2461 (GH, K, MO, NY, P, UC, W); Puerto Guarani, Rojas 13594 (NY); Estancia Gustafson, Rosengurtt B–5481 (K, US); Dpto. General Pacheco, Partido de Pilar, en el bañado del Río “Las Conchas”, Krapovickas 2723 (SMU, US); Al S de Villa Hayes, Rosengurtt B–5622 (US). Presidente Hayes: Pilcomayo Riv., Morong 981 (GH, MO, NY, UC, US); Concepción – Pozo Colorado, Zardini and Guerro 37529 (MO). **Peru**. Ancash: Prov. Santa, Pampa la Grama, Aguilar s.n. (MO 2995176). Arequipa: 15 km W of Arequipa on Hwy to jct. with Pan Americana, SW of Puente Uchumayo, Peterson et al. 20786 (K, US); Carmana–Calderona, Anderson 833 (US); Camana, Anderson 823 (UC). Cajamarca: Prov. Pacasmayo, Dpto. La Libertad, en el desvío de la carretera a Cajamarca, sobre la Panamericana, Sánchez 2988 (MO). Prov. San Miguel, a 3 km de la localidad de Quindén, sobre la carretera a San Miguel, Sánchez 2749 (MO). Huanuco: Weed in the public garden, Macbride & Featherstone 2443 (US). Huarochiri: Dpt. Lima, San Bartolomé, Asplund 11204 (NY). Ica: Prov. Ica, Orillas de la laguna de Huacachina, Cerrate 899 (US); Rio Ingenio, Angulo 2430 (NY). La Libertad: Prov. Trujillo, Haceinda Tanguchi, Angulo 1876 (US); Chicama Valley, Graywood Smyth 46 (US); Prov. Trujillo; Barraza, Sagastegui 7635 (MO); Prov. Trujillo, Hda. Santa Clara, Gagliardi 6516 (GH). Lambayeque: On E side of Chiclayo, Hudson 927 (MO). Lima: Paramanya, Anderson 415 (US); Prov. Alrededores de La Molina, 13 km Este de Lima, Ferreyra 11113 (US); Prov. Huarochiri, San Bartolomé, Asplund 11204 (US). Loreto: Maynas, Playa de Timicurillo just above (ca. 5 km) Baradero de Mazán, sandy beach of Río Amazonas, McDaniel & Rimachi 23074 (MO, US). Piura: Hacienda Buenos Aires, Anderson 573 (UC, US); Despoblado–kil 970, Anderson 931 (UC); Ab. loco, Haught 116 (US). Rosengurtt B-7410 (BAA, P). San Jose: Barra, Herter 81619 (B, MO, UC, US). **Puerto Rico**. San Juan, vertedero area, García and Quevedo s.n. (NY); Area Vertedero, San Juan, Woodbury s.n. et al. (BRIT). **Saudi Arabia**. Eastern: Adh-Dhulayqiyah, Al-Hasa, King Faysal Univ. Res. Farm, Mandaville 7810 (BM). Northern Hijaz Region: Yanbu al-Sinaiyah, Goddard s.n. (K). Province unknown: Hofuf Agric. Res. Centre, Kasasian 1478 (BM); Riyadh, road to Hair, Collenette 4874 (K); km 106.5 on Mecca bypass, Smith 47 (K); Hofuf Agric. Res. Centre, Parker SA126 (BM). **Spain**. Parc Natural del Delta de l’Ebre, Masip s.n. (fragment, BISH). **United States of America**. Alabama: Mobile Co., SE 1.4 of NE 1/4 of Sect. 16, T4S; R2W, Univ. of South Alabama Property, Mobile, SE of Three Mile Ck., Lelong 6460 (NCU); Along US 90–98 causeway over Mobile Bay, Kral 28449 (US); By Battleship Park, along US 90–98 causeway over Mobile Bay, Kral 28449 (GA); Sandy dock area by truck bypass US 98–90 across Riv. from Mobile, Kral 56599 (GA, MO). Arizona: Cochise Co., Chiricahua Mts., Lemmon & Lemmon 360 (UC); Wilcox, Thornber s.n. (ARIZ 147339). Gila Co., Tonto National Forest, Mazatal Mts, Mt. Ord, 1.0 mi. E of Beeline Hwy (SR 87) along FR 626, Gutierrez 1497 (ASU, UTEP). Graham Co., Clifton, Davidson 261 (RSA); 16 km S of Safford on US Hwy 666, Reeder & Reeder 7297 (ARIZ); ca. 2 mi. E of Solomon along US-70, Reeder & Reeder 9269 (ARIZ). Maricopa Co., Hassayampa River Preserve, S end near rest area, Makings 3739 et al. (ASU); 6 mi. E of Mesa, O.S. Stapley Ranch, McLellean & Stitt 555 (ASU); Tonto National Forest, Superstition Wilderness Area, Tortilla Trailhead, E of Tortilla Flat Post Office ca. 6 mi. on Hwy 88, Rice 1348 & Imdorf (ARIZ, ASU); Tonto National Forest, Verde River, ca. 5 mi.below Bartlett Dam, Landrum 9799 et al. (ASU); Scottsdale, Blakley & McCleary B755 (ASU); Lake Pleasant, Pinkava s.n. (ASU 70803); Sierra Estrella Regional Park, Salt River Bed near park entrance, Sundell & Sundell 349 (ASU); Gila Riv. below Salt Riv. confluence, Marler 2204 (ASU); Rio Salado between junctions of Interstate 10 and 7^th^ Ave., Damrel 1958-B & Pacheco (ASU); Salt River at Central Ave., Makings 3679 & Butler (ASU); Salt River at 115^th^ Ave., Rea 853 (ARIZ); Along I-8, ca. 10 mi. W of Gila Bend, Thieret & Brandenburg 53036 (OKL); East of intersection of Avondale Rd. and the Salt River in Tres Rios wetlands, Wolkis 108 et al. (ASU); Mormon Flats Dam, Goodding 2494 (ARIZ); Papago Park, Pinkava & Lehto 6701 (ASU); NW Phoenix, Wilson 3815 (KSTC); Papago Park, Phoenix, Lehr & Weber 1103 (NY); near Phoenix, Orcutt 2518 (US); Biltimore Golf Course, Featherly s.n. (KANU 187777; OKLA 13739); Vicinity of Tempe, Gillespie 8400 (US); Phoenix, Williams 3032 (US); ibid., Griffiths 5891 (US); 11 mi.E of Gila Bend, Wolf 2310 (POM, RSA); Lower Dripping Springs Canyon area, White Tank Regional Park, Keil 6230 (ASU); Riverbed near Phoneix International Raceway, Keil 874 (ASU 70718); Tempe, Judd s.n. (ASU 70720 & ASU 70721); Tempe, Rural Rd. & S.P. railroad tracks, Lehto 1955 (ASU); Tempe, Double Buttes, Keil 874 & Lehto 874 (ASU 70802); ASU Campus, south of Saguaro Hall, Damrel 1094 (ASU); Gilbert, Riparian Preserve at Water Ranch, between Pond #3 and Pond #2 along Whistling Duck Way, Gutierrez 2047 (ARIZ, ASU); Gilbert, near corner of Greenfield and Chandler Heights roads, Landrum 10941 & Pinto (ASU); Tempe, growing in vacant parking lots, McLellan & Stitt 552 (GH); Tempe, McLellan & Stitt 1016 (ASU); 11 mi. E of Gila Bend, Wolf 2310 (GH); Barry M. Goldwater Airforce Range, Sand Tank Mts, Bender Srping, Felger 95-426 et al. (ARIZ, ASU); Palo Verde, ca. 1 mi. S of Wintersburg, Lehto & Crandell 21 (CM); Palo Verde, end of Palo Verde Rd. on N side of Gila River bottom, Landrum 9500 et al. (ASU); ca. 95 to ca. 105 Ave and Gila River, Schuessler & Lehto 17987 (ASU). Mohave Co., Lake Havasu at Toprock, Hevly s.n. et al. (ARIZ 139296). Pima Co., Sabino Canyon, Santa Catalina Mts., Gould 4631 (ARIZ, UC, US); West Branch of the Santa Cruz (Riv.), Church Wash diversion, Mauz 22-024 (ARIZ); Along the Church Wash diversion, Mauz 21-67 (ARIZ); Coyote Mtns, Mendoza Canyon, Reeder & Toolin 8389 (ARIZ). Pinal Co., E of Maricopa, Rossbach 5203 (UC); 3 mi. W of Coolidge, Parker 8261 (RSA, US); Experimental Farm, Sacaton, Peebles 76 et al. (ARIZ); Along AZ-287, ca. 6.5 km W of Florence, Reeder & Reeder 9388 (ARIZ); Picacho Mtns, Wiens 2005-028 (ARIZ); Coolidge, Goodding 101-41 (ASU). Yuma Co., Yuma, Vasey 540 (P, US); Yuma, Silveus 7640 (SMU); 8.6 mi. SW of Hope, Ahles 8899 (MO); Frontage Rd S of Interstate Hwy 8, 1 road mi.W of Aztec, Yatskievych 81-132 (ARIZ); Colorado Riv. bottom near Yuma, Peebles & Harrison 5063 (US); ca. 2 km N of Laguna Dam; Pratt Agr. Lease to BLM, Felger 06-51A et al. (ASU, TEX); Dome Valley, dirt road at Gila Riv. crossing, 0.3 mi. W of Avenue 19E and County 8th Street, Felger 06-61 et al. (ARIZ, ASU); Centennial Wash, 2 mi. SE of Salome near Buckeye-Salome Rd., Butterwick & Hillyard 6392 (ASU); Fortuna Pond, 32°43'25.6"N, 114°27'14.2"W, Felger 06-55 et al. (ASU); Lower Colorado Riv., Dunfee YLD-39 et al. (ASU); Cibola National Wildlife Refuge, G & SR Meridian, Dodson 16 (ASU). Arkansas. Lafeyette Co., Sandy bank of Red Riv. at Spring Ck. Ferry on Ark. 160 W of Gin City and Bradley, Thomas & Thomas 120762 (NY). Lawrence Co., 4 mi. SW of Hoxie, Taylor s.n. (BRIT). California: County unknown: Salto Basin, MacDougal 50 (ARIZ). Escondido Co., Park Lawn, Escondido, Gander 4689 (US). Fresno Co., Selma, currently undeveloped area S of Dinuba Avenue and east of Thomason Avenue, in Young Pond, Snow & Clark 9973 (GREE). Imperial Co., Between Brawley and Westmoreland, Sanders et al. 8746 (RSA); Holtville, 15 ft. below sea level, Parish 8243 (RSA, US); Palo Verde Valley, 5 airline mi. S of Palo Verde, Holmgren and Holmgren 6502 (NY); near Ranger Station, Picacho State Rec. Area, 22 air mi. N of Yuma, McLauglin 2723 & Bowers (ARIZ); Along Colorado River, Quechan Indian Reservation, McLaughlin 3231 & Bowers (ARIZ); Along CA-115 ca. 2 km NW of exit from I-8 E of Holtville, Reeder & Reeder 8157 (ARIZ, ASU); 6.5 km NW of Niland, Reeder & Reeder 8244 (ARIZ, ASU); 1.3 mi. N of Laguna Dam along Hwy S24, McLaughlin 2854 & Bowers (ARIZ); Roadside near Niland, Barr 67-203 (ARIZ); Ferguson Lake, Imperial Nat. Wildl. Ref., McMurry 1407 (ARIZ). Inyo Co., Cow Ck., Gioman 431 (RSA). Kern Co., ca. 6 mi. N of Bakersfield, Nobs and Smith 469 (FSU); Rosedale, Abrams s.n. (RSA); Section 20, 5–10 mi. NW of Wasco, Braun 8 (CM). Los Angeles Co., W. Adams St., Los Angeles, Davidson 247 (KSC); E end of Malibu Lake, Raven & Thompson 14641 (GH, RSA); San Gabriel Mts, West Fork San Gabriel Riv., ¼ mi. westerly from confluence with North Fork, Wheeler 6331 (ARIZ); San Dimas Canyon Dam, San Gabriel Mts., Wheeler 2339 (RSA); Saline flats, Hasse s.n. (MO 768959); Whittier Hills (Puente Hills, pro parte), seepage ca. 300 ft SW of Colima Rd ×Camino del Sur, Ross 4168 (NY, RSA, UC). Merced Co., Big Water Club, E of Gustine, Nobs & Smith 136 (POM); Whittier Hills (Puente Hills, pro parte), 300 ft. SW of Loima Rod ×Camino del Sur, Ross 4168 (ARIZ); Los Banos Wildlife Refuge, 1.5 N of Los Banos, Nobs & Smith 48 (ARIZ). Orange Co., Roadside drainage gutter, along W side of village of Atwood, Wiggins 20336 (RSA); from Back Bay area at Newport Beach, Sawyer 34 (ASU). Riverside Co., Vail Lake area, Temecula Ck. at confluence with lake and sedimentary hills to the east, Boyd & Ross 3801 (MO, RSA); 2 mi. N of Indio, Nobs & Smith 494 (ARIZ); Hemet, Bautista creek Ranches, 45200 Buatista Rd, near Fairview Ave., Lahti s.n. (MU); NW Palomar Mts, Agua Tibia Mts, Along lower Arroyo Seco, S of Dripping Springs Campground, Banks 0808 & Boyd (MU). Coachella, Salton Basin, Reed 4201 (US); N end of Lake Elsinore, Munz and Johnston 11242 (GH, NY, RSA); Batista Canyon Wash, entering San Jacinto Riv. (Wash), just N of Cedar and Mt., NE end of Hemet, Peterson 4989 (AAU, BISH, K, MICH, MO, NY, P, RSA, TAES, UTC, GH, US, WIS); San Jacinto: 8.8 km W of State Hwy 79 on Ramona Expressway towards Lakeview, Peterson & Peterson 8123 (BISH, K, MO, NY, RSA, US); ca. 2 km S of Ripley, Reeder & Reeder 8054 (ARIZ, ASU). San Bernadino Co., San Bernadino, Wilder 1128 (US); Arrowhead Hot Sprgs, San Bernadino Mts., Sanders et al. 13792 (RSA); Loma Linda, Munz and Johnston 8904 (GH); near San Berandino, Parish 2118 (NY); near San Bernadino, Wright s.n. (ARIZ 114027). San Diego Co., Encinitas, Gander 8801 (US); San Diego, Spencer 901 (MU). Larkin's Station, Palmer 404 (MEXU, NA, NY); Irrigating canals, Calexico, Abrams 4000 (GH, MO, NY); San Mateo Canyon Wilderness Area, “Miller Canyon", Boyd et al. 7554 (RSA). San Luis Obispo Co., Estrella Plains, Hwy US 466, 7 mi. E of Paso Robles, Twisselmann 9204 (RSA); San Luis Creek near junction of S Higuera Rd and US Hwy 101, Keil s.n. (ASU 75319). Santa Barbara Co., Roadside ditch ca. 1 mi. E of Buelton, Pollard s.n. (RSA); Iola Vista Tract, Goleta, Pollard s.n. (ARIZ 131363); Along shore of Lake Cachuma near the dam, Santa Ynez River, Smith 5664 (ARIZ). Sierra Co., Gold Lake, Barker 803 (RSA); Santa Barbara R.R. yards, Pollard s.n. (SMU). Tulare Co., Ab loco, Twisselmann 11614 (RSA). Sutter Co., Along Nicolaus Ave., 2.2 mi. NW of the Placer Co., line, 4.4 mi. ENE of Nicolaus, Helmkamp 15559 (ARIZ). Ventura Co., Pool in Ventura Riv. near Mill School, Ventura Ave., S of Foster Park, Pollard s.n. (SMU [no accession number], US 2460463); California Prep. Schol Grounds, Ojai Valley, Pollard s.n. (ARIZ 3317); Canada Larga Creek, near Southern Pacific RR trestle, ca. 1 mi. S of Foster Park, Pollard s.n. (ARIZ 175945). San Antonio Ck. at Hermosa Rd. crossing, Ojai Valley, Pollard s.n. (MO 1970989); San Antonio Ck., between Royal Oaks Dairy and Country Club Drive, Ojai, Pollard s.n. (RSA). Colorado. El Paso Co., Manitou and vicinity, Griffiths 6728 (US). Florida. Bay Co., Roadside on S 77A from US 231, Athey s.n., (WKY). Calhoun Co., Exposed shores of Apalachicola Riv. at Ocheesee Landing, Godfrey 76104 (GA, FSU). Dixie Co., Vicinity of Fanning Spgs, Godfrey 65872 (FSU). Escabmia Co., Spray field of University Mall sewage treatment plant, Burkhalter 4903 (FLAS). Highlands Co., SR 78 1 mi.W of intersection with US 27, Lake Placid, Herndon 1508 (FLAS, RSA). Hillsborough Co., FLA 37 at Polk and Manatec Co. line, 30 mi. S of Lakeland, Baltzell 5318 (FLAS); N of Tampa ca. 7 mi. NE of USF Campus, area between Cypress and Trout creeks, Lakela 24328 (US); Old Memorial Hwy ca. 1.8 mi. N from 580, Lakela 25515 (FSU, GH, NCU, SMU). Indian Riv. Co., Vero Beach, Boudet s.n. (FLAS 59171). Lee Co., N. Fort Myers, graded area on N side Caloosachatchee Riv., beside US 41, Koch 7122 (FSU); Along Caloosahatchee Riv., Ft. Myers, Godfrey 65429 (FSU). Leon Co., ca. 2 mi. E of the intersection of Pensacola and the truck route, Wooten & Sullivan 285 (US); Vicinity of crossing of Centerville Rd. over Interstate 10, Godfrey 72336 (FLAS, FSU, GA, MO, NY, SMU). Levy Co., FLA 24, 3.5 mi. NE of Cedar Key, Baltzell 6897 (FLAS). Manatee Co., N of FLA 70 and W of FLA 674, N of Myakka City, Hendricks s.n. (FLAS 159894). Orange Co., Jones Ave., Zellwood, Scudder 1608 (FLAS). Palm Beach Co., Corbett Wildlife Mgmt. Area, NW of Loxahatchee, Kral 5705 (FSU); ibid., Kral 5707 (SMU). Pinellas Co., Shores of hammock margins, S of Tierra Verde, N of Cunningham Key, Ft. DeSoto State Park, Lakela 27371 (FLAS, RSA). Polk Co., SE corner of Jct of FLA 60 and Doherty Drive in Nalcrest, just N of Lake Weohyakapka, Wheeler s.n. (FLAS 157430). Sarasota Co., SW intersection of Palmer Blvd and Raymond Rd. in Sarasota, Parrish s.n. (FLAS 117137). Seminole Co., Oviedo, Scudder 12 (FLAS). Georgia. Camden Co., Kings Bay Submarine Base, Etowah Park, Carter 13600 (VDB). Chatham Co., White Marsh Island, E of Savannah, Duncan 23451 (GA). Greene Co., Roadside berm 3 mi. NW of Greensboro, Duncan 23177 (GA). Hawaii. Hawaii: Roy Wall Ranch, along jeep trail to Monokaa, Lehuula Mauka Tract, North Kona (PuuLehua Quadrangle), Kawasaki 2 (US); Private lotus farm, Waialuu Beach Rd., Waialua, Imada et al. 92–36 (US). Maui: southern coast of isthmus, Kealia Pond National Wildlife Refuge, Imada et al. 98–22 (US); Lahaina Distr., Honokowai, S end of Honokowai Beach Park, Oppenheimer H89916 (US). Oahu: Kawainui Air Park, Kapaa Quarry Rd., O'Connor s.n. (US 3277650); Kawainui Marsh, entrance to Kapaa Quarry, O'Connor s.n. (US 477673); West Lock U.S. Navy Ammunition Storage Area, just S of West Lock, Pearl Harbor, Eva District, Fosberg 65090 (US). Kansas. Rice Co., 2-3/4 mi. S of Alden, Brooks 17036 (KANU). Illinois: McDonough CO., 1 mi. E of Colchester, Henry 4332 & Scott (MU). Louisiana. Acadia Par., roadside ditch near Bayou Plaquemine, along road between Maxie and Rayne, ca. 7 mi. NW of Rayne, Thieret 18349 (FSU, GA, SMU); Disturbed area on LSUE campus off LA 755 southwest corner of Eunice, Allen 15675 (MO, NLU, NY). Assumption Par., [...] beside LA Hwy 662 ca. 1.5 mi. N of US Hwy 90 at the Amelia Bridge, Thomas & Allen 123995 (GREE, KANU). Beauregard Par., Flat pine woods beside US Hwy 171, 2 mi.S of Longville, Thomas et al. 14488 (ARIZ, GREE, NLU). Bossier Par., Frequent on sand bars along E bank of Red Riv. ca. 7 mi. W of Plain Dealing on LA 2, Allen & Vincent 8525 (GA). Caddo Par., Median strip of Clyde Fant Parkway, NW of Shreve City shopping centre, Shreveport, MacRoberts 1325 (ASU). Calcasieu Par., LA Hwy 14, at junction with Linkswile Rd, 2.1 mi. S of Interstate 210, in Lake Charles, Snow 5803 (MO); Near Interstate 10, Lake Charles, Lonard 2086 (KSTC, SMU). Cameron Par., Back Ridge, Sabine Nat. Wildlife Ref., Valentine s.n. (FSU 72340); Along Gulf of Mexico ca. 1 mi. W of Holly Beach, Thieret & Reese 10039 (FSU, SMU); [...] S of LA Hwys 27 and 82 just E of Holly Beach, Thomas et al. 84819 (GREE, NLU). East Baton Rouge Par., LSU Campus, N of Nicolson Extension and CEBA overflow lot, McKenzie 157 (GA, BRIT); Ben Hur Rd, ca. 200 m NE of jct with Nicholson Rd. (LA 30) in Baton Rouge, Pruski 2687 (US). Grant Par., Red Riv., ca. 0.2 mi. NE of Boyce on LA 8, Vincent 56 (GA); Red Riv. ca. 0.2 mi. NE of Boyce off LA 8, Vincent & Allen 7025 (NCU). Jefferson Par., Grand Island, Town of Grand Island, sandy saline flat, Thieret 25254 (FSU, GA, SMU). La Salle Par., ca. 10 mi. NE of Holloway, Thieret 33501 (FLAS). Livingston Par., 4.4 mi. W of Albany, Shinners 29892 (SMU). Natchitoches Par., Along N bank of Red Riv. at the LA 6 bridge between Clarence and Natchitoches, Thomas & Allen 94291 (NLU, NY); [...] N side of Red Riv. at the LA 6 bridge N of Natchitoches and Grand Ecore, Thomas 114845 (GREE, NLU) and Thomas 114846 (BRIT, FLAS, MO, MONTU, MU, NCU, NLU, NY, OKL, USCH, VPI). Orleans Par., Reclaimed sandy beach, Lake Ponchatrain, Brown 2386 (US); W side of Paris Rd. immediately S of Intracoastal Waterway, Lemaire 1100 (US); Irish Bayou, near junction of I-10 and Hwy 11, Montz 5474 (BRIT); N edge of Irish Bayou, Jctn of Hwy 11 and I-10, SE shore of Lake Pontchatrain, Urbatsch 2553 et al. (ASU). Ouachita Par., Ditch banks beside US 165, just N of Monroe, Thomas & Scurria 36887 (ARIZ, KANU, MICH, MU, NCU). Plaquemines Par., Between levee and Mississippi Riv., just E of Parish Rd. 11, 0.9 mi. from junction with LA Hwy 23, 1.8 mi. S of Port Sulphur, Snow 5789 (MO); S side of LA 39, 0.2 mi. W of the St. Bernard Par. line and 2.1 mi.E of Braithwaite, Thomas & Allen 123787 (MO, NLU, NY); Fort Jackson along the edge of the Mississippi River about 3 mi. SE of Triumph, Taylor & Taylor 21450 (BRIT). Red River Par., 1.5 mi. W of Crichton and about 9 mi. northwest of Coushatta, Peterson 9551 (BISH, K, MO, US); E bank of Red Riv. 1.5 mi. SW of Crichton, ca. 9 mi. NW of Coushatta, Thieret 20615 (A, FSU, GA, SMU, US); Along W bank of Red Riv. S of US 84 bridge at Coushatta, Thomas 114935 (MO, NLU, NY). St Charles Par., ca. 2 mi. E of Bonnet Carre Spillway along S side of IC railroad near LaBranche, Montz 5266 & Cali (BRIT). St. Landry Par., ca. 4 mi. E of P. Barre, on road to Baton Rouge, Thieret 32328 (GA, FSU, NCU); 2.5 mi. E of Port Barre, Thieret 31454 (SMU). St. Mary Parish, Cote Blanche Island, Thieret 16315 (MU). St. Tammany Par., N shore of Lake Ponchatrain near the mouth of the Techfunte River ca. 1 mi. S of Madisonville, Allen 2921 (BRIT, MU). Tensas Par., [...] west bank of the Mississippi Riv. S of the end of LA Hwy 3078 at Port Gibson Ferry, N of St. Joseph, Thomas 86789 (GREE, NLU). Vermilion Par., Along railroad tracks W of LA 82 and S of LA 14 in Abbeville, Thomas 122365 (NLU, NY). West Feliciana Par., Roadbank of LA Hwy 10 ca. 1.4 mi. E of US Hwy 61 and St. Francisville, Thomas 118023 (GREE, NLU). Mississippi. Hancock Co., Miss. Test Facility (NASA) Rogers et al. 3996–a (SMU) and 3996–b (NCU) and 3996–c (MO). Harrison Co., ca. 4 mi. N of Biloxi along Hwy to Hattiesburg, Lasseigne 2811 (MO); NE Biloxi, Lasseigne 2852 (MO); Biloxi, along beach, Rogers 3439-b (NCU). Jackson Co., At I–10, ca. 1.5 mi. W of Alabama State Line, Brooks & McDaniel 530 (FSU). Pearl River Co. Picayune, railroad yard and adjacent places, Rogers 8393 (NCU). Missouri. St. Louis Co., right-of-way of the Missouri Pacific Railroad, S of Loughborough St., Mühlenbach 3910 (MO); Lesperance Street Freigh Yard of the Missouri-Pacific R.R., Central switching tracks, Mühlenbach 2407 (ARIZ). New Jersey. Camden Co., Camden, Parker s.n. (GH). New Mexico. Eddy Co., Carlsbad, Hitchcock 13476 (US). Sandoval Co., Low Roadside, along NM 44, ca. 23 mi. NW of Bernalillo, Henderson 69–352 (USCH). Nevada. Clark Co. Logadale, State Rte. 169 and Whipple Avenue, Gregor 48 (NY); Intersection of Washington and 23rd St., Las Vegas, along drainage ditch at Fremont and Jones, Bostick 3561 (ARIZ); Las Vegas, Hogan 8 (ARIZ); Las Vegas, Lake Mead Blvd., 120 m SE of the intersection of Ranch Rd., Lathrop P–8 (NY); Nevada Test Site, Amargosa Drainage Basin, E of Spring Meadows Farm Hdqtrs, Beatley 13450 (US); Lake Mead, Tilley et al. 1605 (BRY). North Carolina. Guilford Co., West Greensboro, Blonquist 328 (US); W of Greensboro, Blomquist 799 (GH). Orange Co., Soil Conservation Service Nusery, Chapel Hill, Mathews B9#32 (NCU). Oklahoma. Cleveland Co., Near Canadian River S of Hwy 9 off Jenkins St in T8N R8W Sec. 8, Burgess & Larsen 32 (OKL); Canadian River bed, Ten Mile Flats, Lawson & Goodman 609 (OKL); S of Norman near Canadian River at the old municipal landfill, Burgess s.n. (OKL [01-0093243]). Hughes Co., Roadside, 4 mi. SE of Holdenville, Dossett 38 (OKLA). Jefferson Co., Waurika, south side of Hwy 79 bridge at the Red River Crossing, Hoagland et al. BLM0464 (OKL). Love Co., Shoreline on N arm of Hickory Creek, Lake Texhoma, E of Marietta, Harris s.n. (OKLA, SMU); Branch of Lake Texoma called Wilson Creek, ca. 10.8 mi. NW of Marietta, Taylor & Taylor 26010 (BRIT). Pawnee Co., On sand bar in Arkansas Riv., 200 yards N of OK Hwy 18 at bridge at Ralston, Tyrl & McDonald 732 (MO, OKLA). Waggoner Co., 5 mi. N of Muskogee along Arkansas River, Kelting s.n. (OKL [01-0072347]). Washington Co., 0.5 mi. and 2.2 mi. E of Ramona, McDonald 1029 (OKLA). Oregon. Polk Co., ca. 2 mi. N of Monmouth along Hwy 99W, Halse 2330 (NCU); ca. 2 mi. N of Monmouth along Hwy 99W (as weed in greenhouse), Halse 2330 (ARIZ). South Carolina. Berkeley Co., Waste ground around the Santee Wool Combing Mill, Jamestown on SC Rt. 45, Ahles & Haesloop 47009 (NCU) and Ahles & Haesloop 42992 (NCU) and Ahles & Haesloop 38180 (NCU) and Ahles & Haesloop 35618 (NCU) and Ahles & Haesloop 53794 (NCU). Charleston Co., Hydrologic Spoil Area along W bank of Clouter Ck., Charleston Harbor, Porcher s.n. (NCU). Florence Co., Wellman Wool Combing Mill, N of Johnsonville on SC Rt. 41, Ahles & Haesloop 49154 (NCU) and Ahles & Haesloop 46951 (NCU) and Ahles 42896 (NCU). Georgetown Co., Just N of roadbed of old bridge over Great Pee Dee Riv., N side of existing US 17, Waccamaw Point at upper end of Winyah Bay, E side of Georgetown, Nelson & Horn 14619 (GA, USCH). Tennessee. Davidson Co., Weedy on drawdown of borrow pit by Cumbeland River, Metro Center, Nashville, Kral 73090 (APSC). Leon Co., Vicinity of crossing of Centerville Rd. over Interstate 10, Godfrey 72336 (NCU). Shelby Co., Hwy 78 S of Memphis at right across bridge at Drainage Canal, Rogers 33505 (GH). Rutherford Co., Railroad yards by Farmer's Coop, Luvergne [La vernge], Kral 74564 (APSC). Texas. Aransas Co., Rockport, Reverchon 1993 (MO). Brazos Co., Next to Agronomy greenhouses, Texas A & M Univ. campus, Snow & Jensen 184 (TAES); 3 mi. S of Bryan, Ward 165 (OKLA). Brewster Co., Along Rio Grande near mouth of Tornillo Ck., Sperry 1537 (US); bank of Rio Grande at Ogle Spring, Sperry 1348 (US); along Rio Grande nera Mariscal Canyon, Warnock 811 (GH, US); Mariscal Canyon, Chisos Mt. area, Warnock 811 (ARIZ); Boquillas-Chisos Mts, Marsh 140 (SMU); near the Rio Grande, Hot Springs, Big Bend National Park, Goodman & Waterfall 4600 (OKL). Cameron Co., 15 mi.W of Boca Chica along Hwy 14, Anderson 4551 (RSA); Between Tex. Hwy 4 and the beach of Brazos Island, Henderson 78–22 (MO, USCH); 7.3 mi. S of Ed Carey Blvd of Harlingen along US Hwy 83, Snow 5907 (KSP, MO); Brownsville, Benke 5328 (US); Brownsville, Silveus 2565 (SMU); Resaca park, near the girl scouts camp, Runyon 2172 (US); Padre Island, 5 km NE of Port Isabel and across from the Sunchase Mall and McDonalds, Peterson et al. 11157 (BISH, US). Chambers Co., Along brackish swale just off High Island, Gould 7442 (SM, UC); Along entrance to Anahuac Nat. Wildl. Ref., Kessler 5894 (BRIT). Culberson Co., South McKittrick Canyon, Guadalupe Mts., 5 mi. N of Pine Springs, Fischer s.n. (OKLA; OK-13738). Galveston Co., 13 mi.N of Galveston, Gould 11984 (UC, US); Near plant entrance P. H. Robinson Generating Station near Bacliff, Waller 2604 (GH, MO); Tidal flats beneath SH 146 drawbridge over Clear Ck. channel at Kemah, Waller 2607 (GH); West beach in Galveston, ca. 200 m from Gulf of Mexico, Kessler 5603 (BRIT); Coastal marsh at Texas City, Van Devender & Van Devender 88-262 (ARIZ). Harris Co., Morgans Point, Palmer 11963 (UC); Houston, Fisher s.n. (ARIZ 147207); Near the Houston Ship Channel in the San Jacinto State Park, Brown 2557 (ARIZ); East side of Houston, Thieret 30813 (SMU). Hidalgo Co., East of Mission, Coover 1336 (ARIZ); Alamo, Clover 918 (MICH); Anzalduas County Park, ca. 2.7 mi.W of Mission, ca. 5 mi. W of McAllen, Snow 5906 (KSP, MO); ca. 1.4 mi. E of US Hwy 281 on Rd. 490, Snow & Evans 4393 (GREE, RM); [...] FM Rd. 2062 just N of Bensten State Park and W of Mission, Thomas et al. 39476 (GREE). Jefferson Co., Coastal marsh, Tharp s.n. (GH). Jim Wells, Co., Along US Hwy 281 ca. 35 air mi.WSW of Corpus Cristi, ca. 4.5 mi. N of Premont, Snow 5897 (KSP, MO). Kenedy Co., Sarita, Hitchcock 5447 (US); Sarita, Hitchcock 5463 (US); Alazan Well on Payne Ranch (Julian Quadrangle), Carr 20282 et al. (BRIT); 0.5 mi. S of Sarita in drainage between divided Hwy 77, Peterson 9555 (BISH, GH, K, MO, NY, P, RSA, TAES, US, UTC); Near Las Norias, Runyon 3829 (RSA). Kleberg Co., Banks of Tranquitos Ck., first 150 metres, W of US Hwy 77 in Kingsville, Snow 6414 (KSP, MO); ca. 30 air mi. SSW of Corpus Cristi, inside Seawind RV Resort, adjacent to Kaufer–Hubert Memorial Park, Snow 5916 (KSP, MO, MU); Kingsville, Reed 6 (US). La Salle, Cotulla, Planck 32 (US); Rivera, Silveus 186 (SMU). Matagorda Co., 5 mi.E [of] Wadsworth, Wilson 15144 (KSTC). Nueces Co., Corpus Cristi, Silveus 126 (MU, US); ibid., Silveus 2407 (SMU). Red Riv. Co., On sand bar, Old Pine Bluff ferry crossing across Red Riv., ca. 3.5 mi. W of Kanawha, Correll 37906 (NY). San Patricio Co., 2 mi. E of Taft, Harvey & Elliot 7351 (MICH); Shore of Lake Mathis, Gould 11467 (UC); 5 mi. W of Aransas Pass, Webster 7079 & Rowell (SMU); South banks of Chiltipin Ck., just E of US Hwy 77 overpass, ca. 2 air mi. W of Sinton city centre, Snow 6394 (KSP, MO); 0.4 mi. S of Jct of FM 892, ca. 4.7 mi. S of Robstown, Snow 5919 (KSP, MO); US Hwy 77, along Chiltipin Ck. just N of Sinton, Snow & Evans 4389 (RM); Gregory, Reed s.n. (FLAS 167640, USCH); Big Lake area of Welder Wildlife Foundation, Penn 53 (GA); S side of Corpus Cristi, Jones 1142 (NCU, SMU). Starr Co., Town of Garciasville, along RR tracks, Snow 5904 (MO); Rio Grande City, Griffiths 6486 (US). Val Verde Co., Mouth of the Pecos Riv., Hinckley and Hinckley 412 (SMU, US). Victoria Co., Along US Hwy 77, ca. 1.1 mi. N of Coleto Ck., a few air mi. SW of Victoria, Snow 6372 and 6373 (MO). Webb Co., ca. 4 mi. SE of Laredo, Zapata 20 (BRIT); Casa Blanca Lake, 3 mi. NE of Lardo, Alvarez 7432 (OKLA); Lake Casa Blanca, 6 mi. NE of Laredo, Ramirez 2 (SMU); Laredo, Fisher 252 (US); Lake Casa Blanca, 6 mi. NE of Laredo, Lozano 17 (BRIT); ibid, McCart et al. 1 (OKLA); Laredo, Orcutt 5574 (MO); Lake Casa Blanca, 6 mi. E of Laredo, García et al. 8731 (FSU, GA); Lake Casa Blanca, 6 mi. E of Laredo, Ramírez 6 (BRIT); Bank of Rio Grande near Lardo, Barkley 13E016 (NCU). Willacy Co., 2–1/4 mi.N of Raymondville, Cory 51477 (MICH, NY, SMU); Route 497, 19 mi. E of Raymondville, Correll and Johnston 17879 (MO, NY, TEX, US). Zapata Co., Saline arroyo 3 mi. N of San Ygnacio, Correll & Johnston 18083 (NCU, SMU, US); End of Texas FM 496, ca. 2.5 mi. W of Zapata, gravelly shores of Falcon Lake, Snow 5899 (MO); ca. 250 m S of end of Texas FM 496, ca. 2.5 mi. W of Zapata, Snow 5902 (KSP, MO); 11 mi. N of San Ygnacio, Shinners 17668 (SMU). Utah. Utah Co., North Park, Provo, Mengies 8001 (BRY, MO). Virginia. King William Co., End of Eltham Bridge and railroad, VA 30 and 33 in West Point, Bradley 24033 (GA). Warrick Co., Chrome ore piles, Newport News, Reed 44052 (US). **Uruguay**. San Jose: Barra, Herter 519 (GH, M, NY). **Venezuela**. Anzoategui: Guanta and Puerto La Cruz, Tamayo 2057 (US); Dsto. Bolívar, Swamp at Boca de Tigre, at the intersection of the road to Bergatín and Barcelona–el Crucero Rd., Davidse and González 20010 (MO, NY). Falcon: Coro, 3 km E del centro, Wingfield 5184 (MO); 12 km SE de Coro, entre La Negrita y Siburúa, Wingfield 5095 (MO). Sucre: Lagunas Litorales de Cumaná, Cumana Campos 519 (NY, US).

#### Diplachne gigantea

Taxon classificationPlantaePoalesPoaceae

Launert, Bol. Soc. Broteriana, ser. 2a, 47: 394. 1974.

Figures 9 , 10

Leptochloa gigantea (Launert) Cope and N. Snow, Novon 8: 79. 1998.

##### Type.

ZAMBIA. Mbala (Abercorn), Saisi River (near Jerico), 27 Feb 1958, floodplain (in swamp) in ditch at side of embankment, LDF Vesey-Fitzgerald 1551 (holotype: K!; isotype: BM!, K!).

##### Description.

Plants perennial. Culms to nearly 300 cm tall, 5–10 mm wide at base, round, erect, arising from thickly rhizomatous root crowns, branching; nodes glabrous; internodes mostly 5–18 cm long, firm but spongy (aerenchymatous) in centre. Leaf sheaths usually much longer than internodes, sericeous but becoming glabrous with age, the margins ciliate; ligules to 14 mm long, membranous, attenuate but becoming lacerated; blades 30–65 ×0.5–0.65 cm, linear, tightly involute on drying, glabrous to slightly scabrous above and below. Panicles 50–80 ×ca. 25 cm wide, exserted with 19–44 branches; the branches 12–18 cm long, alternate or whorled along the rachis, somewhat reflexed to ascending, flexuous, minutely scabrous, the axils scabrous to short pilose. Spikelets 8–13 mm long, distant near base of branches to imbricate at tips; florets (6–)10–15; callus glabrous; lower glumes 2.7–3.4 mm long, narrowly ovate to ovate, scabrous on midnerves, acute to obtuse; upper glumes 2.5–4.0 mm long, otherwise similar to lower glumes; lemmas 3.6–4.8 mm long, narrowly ovate to ovate, dark green, the lateral nerves faint, appearing glabrous but sparsely sericeous upon close inspection, apex acute to attenuate, awnless or with mucros to 1.3 mm long; paleas subequal to lemma, elliptic to narrowly ovate, sparsely sericeous on nerves; apex acute. Stamens 3; anthers 1.9–2.5 mm long, purple or yellow. Caryopses ca. 1.8 mm ×ca. 0.6 mm, elliptic in hilar side profile, depressed obovate in transverse section, hilar groove lacking, smooth but slightly uneven; pericarp apparently weakly adnate to the endosperm.

**Figure 9.**
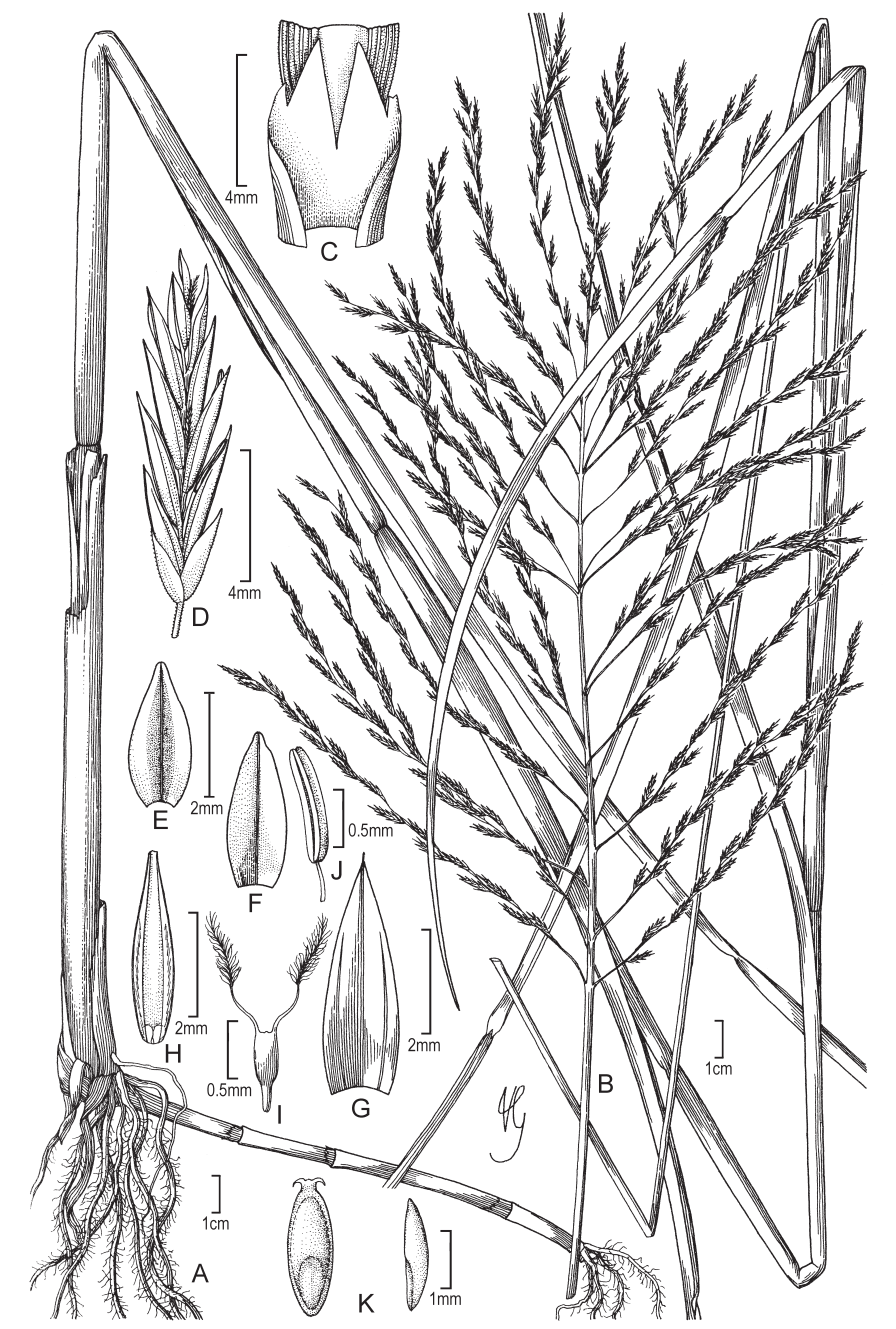
**A–K** *Diplachne gigantea* Launert **A** habit **B** inflorescence **C** ligule **D** spikelet **E** lower glume **F** upper glume **G** lemma **H** palea and lodicules **I** ovary and stigmas **J** stamen **K** caryopsis, dorsal view (left) and lateral view (right). Drawings from McCallum Webster A305 (K), from Zambia.

##### Leaf anatomy.

Not studied.

##### Stem anatomy.

**Figure 10.**
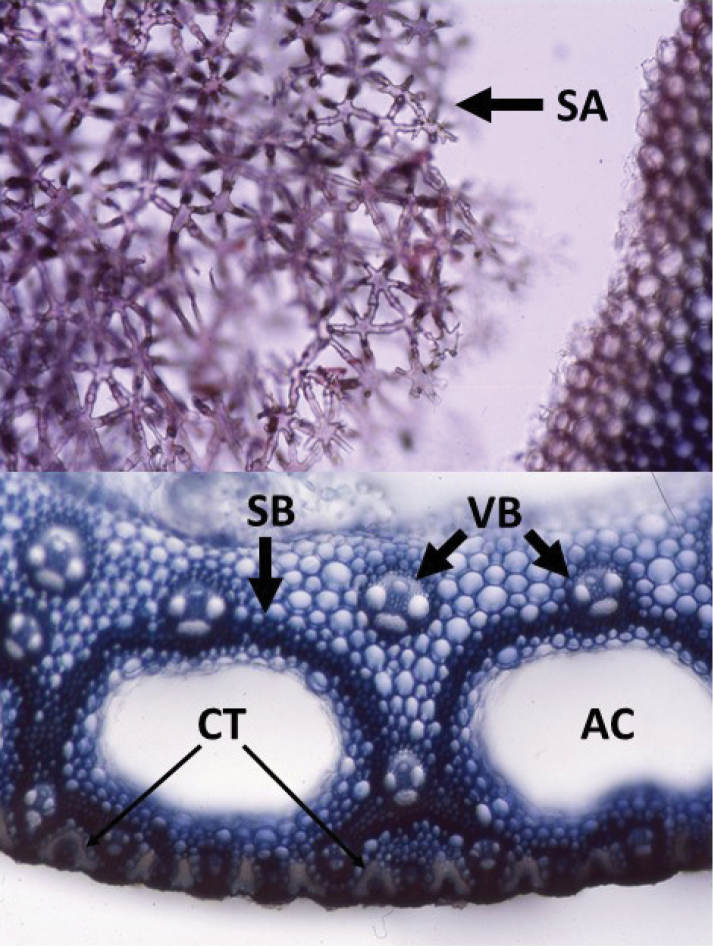
Stem anatomy of *Diplachne gigantea*. Upper: Stellate aerenchyma (**SA**) that occupies much of the central portion of the culm (voucher: Smith 4126 [MO]). Lower: Outer portion of stem showing air canals (**AC**), chlorenchymatous tissue (**CT**), vascular bundles (**VB**), and sclerenchyma bands (**SB**) surrounding the air canals (voucher: Simon & Williamson 2025 [BM]).

The stellate aerenchyma of *D. gigantea* probably reflects adapations for its emergent growth habit in aquatic situations. A series of relatively large, elliptic air canals are subjacent to the epidermal layers (Fig. 10). Chlorenchymatous tissue is also present near the surface. Peripheral sclerenchymatous ring present and linked to outermost vascular bundles via girders. Inner sclerenchymatous ring canal tissue present. Sclerenchymatous rings surrounding vascular bundles located inside inner sclerenchymatous ring present. Kranz sheath cells present, with vascular bundles present in outer portions of sheaths; Kranz sheath cell canal tissue present. Vouchers: Simon & Williamson 2025 (BM); Smith 4126 (MO).

##### Chromosome number.

Unknown.

##### Phenology.

Flowering December through May.

##### Distribtution.

**Native**: A few widely scattered collections from Botswana, Tanzania and Zambia. Growing in edges of swamps and margins of slow moving rivers. Elevation 1250–1710 metres, although upper and lower ranges uncertain. (TDW: BOT, ZAM, TAN). **Non-native**: See below (cultivated) in Zimbabwe.

##### Conservation status.

Data Deficient (IUCN 2012). However, detailed localities are provided below given the evident rarity of *Diplachne gigantea*, which apparently has not been collected since 1983. Focused attempts by the first author in January of 1996 to re-collect at the two localities of Pete Smith in the Okavango region of Botswana were unsuccessful, even after speaking with Smith regarding his knowledge of the species. To the best of the authors' knowledge, Smith is the last person to have collected the species. He knew of no vernacular name for the species in Botswana which, if it existed, might help in relocating efforts and indicated that each of the two populations from which he had collected were small. He confirmed, as some herbarium specimens labels and the stellate culm anatomy suggest, that the species is an emergent aquatic that can continue to grow on sandbars.

More specifically, on 28 and 30 January 1996 the first author hired a boat and driver at Drotskys Cabins in Botswana, located approximately 20 km south of the Namibian boarder (Caprivi Strip) and approximately 7 km southeast of the small town of Shakawe (Highway A 35). One entire day each was devoted to searching for *D. gigantea* moving southeast downstream on the Okavango River and returning by the same route on the opposite bank of the river and upstream and back from the same point of departure. No individuals were seen. Although only speculative, two hypotheses concerning the evidently limited distribution in relation to its habitat merit consideration. First, the stellate aerenchyma of the culm likely contributes to a relatively soft texture, which may render it particularly palatable for large grazers such as hippopotamus (*Hippopotamus amphibius*), which are common in the Okavango Delta and which consume large quantities of food. Second, given the extensive potential habitat for *D. gigantea* in the Okavango Delta, *D. gigantea* may occur more frequently than herbarium records suggest. However, given the paucity of collections to date, it likely is uncommon anywhere in its range.

*Diplachne gigantea* was cultivated in the early 1980s at the Lakeside National Botanic gardens in Zimbabwe (*Browning 8*; A, P). Gibbs Russell et al. (1991: 118) indicate that *D. gigantea* has been collected at the western edge of the Caprivi Strip in Namibia but the authors have not seen a voucher.

In summary, the data suggest that *Diplachne gigantea* is rare and additional knowledge of its distribution, ecology and relative abundance are critical for making a recommendation following IUCN standards.

##### Etymology.

The word *gigantea* refers to the large stature of the species.

##### Vernacular name.

Giant diplachne.

##### Comments.

*Diplachne gigantea* is easily diagnosed from *D. fusca* by its much taller stature at maturity, its evidently obligate and usually emergent growth in hydric habitats, the stellate aerenchyma of the culms, the absence or near-absence of lemmatal macrohairs and the relatively lax panicle branches. The collection from cultivation at the Lakeside Botanic Gardens (see below) is indicated as growing from a “dense mat at the base", presumably meaning the culms arose from short rhizomes.

The fourth author personally recalled the species as being fairly commonly locally at the time of his collection in 1983 (Simon & Williamson 2025).

##### Specimens examined.

**Botswana**. Ngamiland: Okavango River, sandbank just recovering from being inundated by high waters during the previous six weeks, 18°34.25'S, 22°06.3'E, 8 May 1985, Smith 1387 (BRI, K, MO); In water 1 m deep in a backwater off the mainstream of the Okavango River among water lillies, reeds, 18°20.2'S, 21°49.9'E, Smith 4126 (K, PRE, SRGH [n.v.]). **Tanzania**. Reported but not confirmed from the Iringa District (Phillips 1974). **Zambia**. Kabompo: Bog by river 100 yards from Customs point [near the Tanzanian border], McCallen Webster A305 (BM, K). Mbala (Abercorn): 70 mi. S of Mwinilunga on Kabompo Rd., rooted in water, +/- 1250 metres elev. (ca. 4,100 ft), 25 Dec 1969, Simon & Williamson 2025 (K, BM, SGRH [n.v.]). **Zimbabwe**. Cultivated at Lakside Botanic Gardens in 1983 from an unknown source, Browning 8 (A, P [P06795263]!).

### Excluded names

The following names, for which the authors have confirmed the type specimens, are not in *Diplachne*.

***Diplachne alopecuroides*** Hochst. ex Steud., Syn. Pl. Glumac. 1(3): 248 (1854) = **Leptocarydion vulpiastrum** (De Not.) Stapf.

***Diplachne andropogonoides*** (Steud.) Nees., Fl. Afr. Austral. Ill. 258 (1841) = **Triraphis andropogonoides** (Steud.) E. Phillips.

***Diplachne arenaria*** Nees ex Steud., Nomencl. Bot. [Steudel], ed. 2. i. 514; et ex Hochst. in Flora, xxxviii. (1855) 427, nom. inval. = **Trichoneura mollis** (Kunth) Ekman

***Diplachne aristata*** Baker, J. Linn. Soc., Bot. 22: 534 (1887) = **Neyraudia arundinacea** (L.) Henrard.

***Diplachne barbata*** Hack., Oesterr. Bot. Z. 52: 240. 1902. **Type.** Brazil. Pernambuco, Boa Viagem, in arenoosis, Schenck 4310 (holotype: W-1916-0017694!; viewed digitally); isotype (fragment): US [00478602]!) = **Gouinia barbata** (Hack.) Swallen.

***Diplachne baueri*** R. Br. ex Desv., Mém. Mus. Nat. 5: 272 (1819), nom. inval.

***Diplachne biflora*** Hack. ex Schinz., Bull. Herb. Boissier iii: 387 (1895). **Type.** South Africa. Transvaal, Makapansberge, Streydpoort, A. Rehmann 5386 (holotype: W!; isotype: K [K000366556!, K000366557!], US [fragment; US-865880]!) = **Bewsiabiflora** (Hack. ex Schinz.) Gooss.

***Diplachne biflora*** var. ***buchananii*** Stapf, Flora Capensis 7: 593 (1900). **Type.** Letsotho (“Basutoland"), Leribe, *Buchanan 219* (holotype: K [K000366556]!) = **Bewsiabiflora** (Hack. ex Schinz.) Gooss.

***Diplachne brandegei*** Vasey, Proc. Calif. Acad. Sci., ser. 2, 2: 213. 1889. **Type.** Mexico. Baja California Sur, Magdalena Island, Brandegee 11 (lectotype, designated by Hitchcock (1913): US [00133605]!) = **Enteropogonbrandegei** (Vasey) Clayton

***Diplachne brevifolia*** J. Presl, Reliq. Haenk. 1 (4‒5): 261 (1830). **Type.** Peru. Habitat in montanus huanoccensibus, T. Haenke s.n. (holotype: PR; isotypes: MO (MO-2114159; MO-2114160 [line drawing]; US, fragment [2875386]) = **Festuca rigescens** (J. Presl) Kunth.

***Diplachne bulgarica*** J. Presl, Reliq. Haenk. 1(4-5): 261 (1830). **Type.** unknown.

***Diplachne caudata*** K. Schum., Pflanzenw. Ost-Afrikas C. 113 (1895). **Type.** Kenya. Nairobi, R.A. Dümmer 1981 (holotype: K [K000366659]!) = **Dinebracaudata** (K. Schum.) P.M. Peterson & N. Snow.

***Diplachne bulgarica*** (Bornm.) Roshev., Fl. USSR 2: 230, 751 (1934). **Type.** = **Cleistogenesserotina** (L.) Keng.

***Diplachne cearensis*** Ekman, Ark. Bot. 10(17): 32, t. 5, f. 3, t. 6, f. 18 (1911) **Type.** Brazil: Ceara, in silvis caatinga dictis viam entre Crato et Barbalha, Loefgren 672 (holotype: S, n.v.; isotype: US, fragment [00478599]!) = **Gouiniacearensis** (Ekman)

***Diplachne chloridiformis*** Hack. ex Stuck., Anales Mus. Nac. Hist. Nat. Buenos Aires 13: 498 (1906). **Type.** Argentina: Córdoba: Río Seco, Stuckert Herb. Arg. 2329a (lectotype, designated by Nicora 1995; isolectotypes: W!) = **Leptochloachloridiformis** (Hack. ex Stuck.) Parodi.

***Diplachne dominguensis*** (Jacq.) Chapm., Fl. South. U.S. (ed. 3) 609 (1897). **Type.** Dominican Republic: Annonymous s.n. (holotype [probable]: W [W-0027107]! Laegaard (in 1990, see specimen label) interpreted this collection to be the holotype, which appears sound given “Hb. Jacq." on what appears to be the oldest affixed label, and “Herbar, Jacquin Fil.", on an evidently younger label, referring to Jacquin's son J.F. Jacquin = **Leptochloavirgata** (L.) P. Beauv.

***Diplachne dubia*** (Kunth) Scribn., Bull. Torrey Bot. Club 10(1): 30 (1883). **Type.** Mexico: Humboldt and Bonpland 4172 (lectotype [designated by Snow et al. 2008]: P!; isolectotypes: K, K-microfiche, US-865876 (fragment ex P!)) = **Disakisperma dubium** (Kunth) P.M. Peterson & N. Snow.

***Diplachne dubia*** var. ***aristata*** Vasey, Proc. Calif. Acad. Sci., ser. 2, 2: 213 (1889). Nom. nud. = **Disakisperma dubium** (Kunth) P.M. Peterson & N. Snow.

***Diplachne dubia*** var. ***kurtziana*** Kuntze, Revis. Gen. Pl. 3(3): 349 (1898). **Type.** Argentina: Córdoba, Kurtz 6647 (holotype: NY; fragment, US-86574!) = **Disakisperma dubium** (Kunth) P.M. Peterson & N. Snow.

***Diplachne dubia*** var. ***pringleana*** Kuntze, Revis. Pl. 3(3): 349 (1898). **Type.** U.S.A. Arizona: Sierra Tucson, 27 April 1884, Pringle s.n. (holotype: MSC-8177; isotypes: GH, US, VT) = **Disakisperma dubium** (Kunth) P.M. Peterson & N. Snow. (See Mauz (2011) for discussion compared to Snow (1997a) and Peterson et al. (2001)).

***Diplachne fleckii*** Hack., Bull. Herb. Boissier 4 (App. 3). 25. 1896. **Type.** Namibia. Rehoboth, Fleck s.n. (holotype: Z [Z000074830]!) = **Pogonarthriafleckii** (Hack.) Hack.

***Diplachne gatacrei*** Stapf, Bull. Misc. Inform. Kew 1989(141): 229. **Type.** Pakistan [“North India"], Gatacre 17626 (holotype: K [000245007]!) = **Kengiagatacrei** (Stapf) T.A. Cope

***Diplachne guatemalensis*** Hack., Oesterr. Bot. Z. 52: 275. 1902. **Type.** Guatemala, Friedrichsthal 1748 (holotype: W! [W-17685]!) = **Gouinialatifolia** Vasey var. **guatemalensis** (Hack.) J.J. Ortíz.

***Diplachne halei*** Nash, New York Bot. Gard. 1: 292. 1889. **Type.** U.S.A. Louisiana, Hale s.n. (lectotype, here designated, NY [00019504]!; isolectotypes: GH [GH00023584]!, US [US00133607]!) = **Dinebrapanicoides** (J. Presl) P.M. Peterson & N. Snow. The selected lectotype is from Nash's own herbarium, purchased by NY in 1911.

***Diplachne latifolia*** (Griseb.) Hack., Oesterr. Bot. Z. 52: 274 (1902). **Type.** Argentina, Córdoba, prope Aschochinga, Lorentz & Hieronymous 256 (lectotype: GOET), designated by Swallen, Amer. J. Bot. 22: 39 (1935) = **Gouinialatifolia** (Griseb.) Vasey.

***Diplachne loliiformis*** (F. Muell.) Benth. = **Tripognella loliiformis** (F. Muell.) P.M. Peterson & Romasch (see Peterson et al. 2016)

***Diplachne mendocina*** (Phil.) Kurtz, Bol. Acad. Ci. (Cordoba) 15: 521 (1897). **Type.** Argentina, Mendoza (holotype: SGO-PHIL-356; isotypes: BAA, SGO, US (US-899255 [fragment ex USDA Herb. ex SGO-PHIL) = **Disakisperma dubium** (Kunth) P.M. Peterson & N. Snow

***Diplachne mexicana*** (Scribn.) Hack., Oesterr. Bot. Z. 52: 275 (1902). **Type.** Mexico, San Luís Potosí, Tamasopo Cañon, Pringle 3252 = **Gouiniamexicana** (Scribn.) Vasey.

***Diplachne menyharthii*** Hack., Bull. Herb. Boissier ser. 2. 1: 772. 1901. **Type.** Mozambique. Boruma am Sambesi, L. Menyhart 1162 (holotype: W; isotype: US, fragment [01231630]! = ***Pogonarthria menyharthii*** (Hack.) Hack.

***Diplachne monticola*** (Chase) McNeill, Brittonia 31(3): 401 (1979). **Type.** Haiti. Vicinity of Furcy, common on summit of Pic de Brouet, vicinity of Furey, Leonard 4751 (holotype: US! [00134132]; isotypes: B!, NY!, US! [00516790]) = ***incertae sedis***. Its correct generic placement may be within *Gouinia* given the general resemblance of its spikelets to other species of this genus.

***Diplachne panicoides*** (J. Presl) McNeill, Brittonia 31(3): 402 (1979). **Type.** Mexico, Habitat in Mexico ad Acapulco, verosimiliter in lutosis, Haenke s.n. (holotype: PR!; isotypes: LE-TRIN-2121.02!, MO-2109567!, US, fragment [US78688!] = **Dinebrapanicoides** (J. Presl) P.M. Peterson & N. Snow.

***Diplachne patens*** (J. Presl) E. Desv., Fl. Chil. 6: 371 (1854). **Type.** Chile. Coridellra chilensibus, Haenke s.n. (holotype: PR; isotype: US-A78816!) = **Disakisperma dubium** (Kunth) P.M. Peterson & N. Snow.

***Diplachne peacockii*** Maiden & Betche, Agric. Gaz. New South Wales 15: 925, with plate (1904). **Type.** Australia. New South Wales. Coolabah, 4 Dec 1904, Maiden & Boorman s.n. (lectotype: NSW-126631!, designated effectively by Lazarides (1980); isolectotypes: BM!, BRI!, K!, W!) = **Dinebradecipiens** (R. Br.) P.M. Peterson & N. Snow subsp. **peacockii** (Maiden & Betche) P.M. Peterson & N. Snow

***Diplachne* “poaeiformis**" Hochst., uncertain if name published (see: http://apps.kew.org/herbcat/getImage.do?imageBarcode=K000366402) = **Bewisa biflora (Hack.) Goossens**?

***Diplachne pringlei*** Vaset ex Beal, Grass. N. Amer. 2: 436 (1896), nom. inval. pro syn *Leptochloa pringlei* Beal = **Disakisperma dubium** (Kunth) P.M. Peterson & N. Snow.

***Diplachne pungens*** Hack., Bull. Herb. Boiss. 4, app. 3: 25. 1896. **Type.** Namibia. Horebis, Fleck s.n. (holotype: W!, fragment of type: PRE!; isotypes: US! [00478606, fragment]; W-1916-0017605!) = **Odyssea paucinervis** Stapf

***Diplachne reverchonii*** Vasey, Bull. Torrey Bot. Club 13(70: 118 (1886). **Type.** U.S.A. Texas. Llano Co., collected on granite rocks, Reverchon 1613 (lectotype, here designated: US [00133610]!; isolectotypes: CM!, MO! [123188, 123189], VT) = **Tripogon spicatus** (Nees) Ekman. A previous worker annotated the numbered collection (Reverchon 1613) at MO as isotype or probable isotype, which was incorrect given that no specimen was cited in the protologue.

***Diplachne rigida*** Vasey, U.S.D.A. Div. Bot. Bull. 12(2): t. 44 (1891). **Type.** U.S.A. Texas. Reverchon 30 (holotype: US [00133611]!) = **Eragrostis sessilispica** Buckley.

***Diplachne scabra*** (Nees) Nicora, Hickenia 2(19): 91 (1993). **Type.** Brazil. Parà, in ripa fluminum Amazonum, von Martius s.n. (holotype: M!; isotypes: BAA-1514 (fragment ex M), US-88699! (fragment ex M) = **Dinebra scabra** (Nees) P.M. Peterson & N. Snow.

***Diplachne*** “sessilis Pilg. ms." Listed as type at Z but the name evidently never published.

***Diplachne serotina*** (L.) Link var. **chinensis** Maxim., Bull. Soc. Imp. Naturalistes Moscou 54: 80. 1879. **Type.** Japan, Maximowicz s.n. (lectotype: LE, chosen by Phillips and Chen [Novon 15(3): 464 {2005}]) = **Kengiaserotina** Link var. **chinensis** Maxim.

***Diplachne simplex*** Döll var. ***simplex*** Döll, Fl. Bras. 2(3): 97 (1878), nom. inval. **Type.** Brazil. Minas Gerais. Habitat in prov. Piauhy, Gardner 2367 (US, fragment!) = **Tripogon spicatus** (Nees) Ekman.

***Diplachne simplex*** var. ***uralepedia*** Döll, Fl. Bras. 2(3): 98 (1878). **Type.** Brazil. Minias Gerais, in prov. Minas Gerais, Widgren s.n. (isotype: US, framgent [US 00478068]!) = **Tripogon spicatus** (Nees) Ekman.

***Diplachne sinensis*** Hance, J. Bot. 8: 76 (1870). **Type.** China. Hebei, Beijing, “in collibus", Aug. 1865, Williams 12572 (holotype: BM [BM 000514995], not seen) = **Cleistogenes hancei** Keng.

***Diplachne scirpifolia*** J. Presl, Reliq. Haenk. 1(4-5): 261. 1830. = **Festuca dolichophylla** J. Presl (see Catalán and Müller 2012).

***Diplachne songarica*** Roshev., Japanese J. Bot. 18: 540 (1992). **Type.** China. Nei Mongol.: Baileng temple, Keng 3378 (holotype: NAS, not seen) = **Cleistogenes songorica** (Roshev.) Owhi.

***Diplachne spicata*** (Nees) Döll. ex Benth., J. Linn. Soc., Bot. 19: 111. 1881 [1882]. **Type.** Brazil, Piauí, habitat in campis, Campo mimoso dictis, Martius s.n. (holotype: LE-TRIN-2313.01, not seen) = **Tripogonella spicata** (Nees) P.M. Peterson & Romasch (Peterson et al. 2016).

***Diplachne squarrosa*** (Trin. ex Ledeb.) Maxim. Bull. Soc. Imp. Naturalistes Moscou 54(1–2): 71 (1879). **Type.** China. Altai Mountains, Bongard s.n. (isosyntype [possible]: MO! [MO-2114888, viewed digitally]) = **Cleistogenessquarrosa** (Trin. ex Ledeb.) Keng.

***Diplachne squarrosa*** var. ***longiaristata*** Rendle, J. Linn. Soc., Bot. 36(254): 411–412 (1904). **Type.** China. Syntypes at BM need to be examined and a lectotype chosen.

***Diplachne tectoneticola*** Backer, Bull. Jard. Bot. Buitzenzorg, sér. 3, 2: 326 (1920). **Type.** Indonesia. Java, Kangean, in tectonetis, Backer 27726 (lectotype, here designated, BO!) = ***incertae sedis*** (molecular placement within Chlorideae unknown). Remaining syntypes: Backer 27770, Beumee 4716.

***Diplachne thoroldii*** Stapf ex Hemsl., J. Linn. Soc., Bot. 30: 121. (1894). **Type.** China (Xizang), Thorold 120 (holotype: K! [K000907432, viewed digitally]) = **Orinus thoroldii** (Stapf ex Hemsl.) Bor.

***Diplachne tolucensis*** (Kunth) Sprengl., Syst. Veg. (ed. 16) [Sprengel] 1: 351 (1824). **Type.** Mexico, inter Islahuaca et Toluca, Humboldt & Bonpland s.n. (holotype: P [P00669425]!; isotypes: B, BM, P [P00128981]!, [P00128982]!, P[P00625338]!) = **Festucatolucensis** Kunth.

***Diplachne virgata*** (J. Presl) Hack., Oesterr. Bot. Z., 52: 275 & 276 (1902). Bromus virgatus J. Presl., Reliq. Haenk. 1 (4-5): 263. (1830). **Type.** Mexcio. Guerrero, Acapulco. Haenke s.n. (holotype: PR, not seen; isotypes: MO-134696 [MO-144080]!) = **Gouiniavirgata** (J. Presl) Scribn.

***Diplachne viscida*** Scribn., Bull. Torrey Bot. Club 10(3): 30 (1883). **Type.** status unconfirmed = **Dinebra viscida** (Scribn.) P.M. Peterson & N. Snow.

***Diplachne vulpiastrum*** (De Not.) Schweinf, Abh. Preuss. Akad. Wiss. (1894) 35 et 38. Rabdochloa vulpiastrum De Not., Flora Capensis 7: 648 (1900). **Type.** status unconfirmed = **Leptocarydion vulpiastrum** (De Not.) Stapf.

### Unconfirmed names

The following names have been associated with various subspecies of *Diplachne fusca* (Snow 1997a), but the authors cannot confirm them at the present for want of having seen type specimens.

Names associated with Diplachne fusca subsp. fusca:

*Diplachne alba* Hochst., Flora 25(1), Beibl. 134. 1842. Nom. nud.

Diplachne capensis (Nees) Nees var. pauciflora Nees, Fl. Afr. Austr. 257. 1841. **Type.** South Africa. In districtus Caledon montibus. Since Poaceae types of Nees were destroyed at B, selection of a neotype may be necessary.

Diplachne fusca var. lutescens Probst & Thell., Vierteljahrsschr. Naturf. Ges. Zürich 438. 1907. **Type.** R. Probst, s.n. According to TL2, Probst's herbarium is in Städtische Sammlungen Biberach an der Riss (specimen not seen).

*Poa procera* Roxb., Fl. Ind. 1: 334. 1820. Nom. nud., Hort. Bengal. 82. 1814; Fl. Ind. ed. Carye and Wall., 1: 334, 1820.; Fl. Ind. ed. Carey 1: 332. 1832. *Eragrostis procera* (Roxb.) Steud., Syn. Pl. Glumac. 1: 266. 1854.

*Poa senegalensis* Desf., Cat. Pl. Paris, ed. 3: 21, 387. 1829. Nom. nud., non Desv.

*Tridens capensis* Nees, Linneana 7: 324. 1832. *Uralepis capensis* (Nees) Kunth, Enum. Pl. 1: 319. 1833. *Diplachne capensis* (Nees) Nees, Fl. Afr. Austr. 256. 1841. *Triodia capensis* (Nees) Th. Dur. and Schinz, Consp. Fl. Afr. 5: 877. 1895. **Type.** SOUTH AFRICA. “Bei Doornhoogte in der Capschen Fläche, Dec. 1824, Ecklon". No specimen is cited in the protologue; lectotypification is probably needed.

*Uralepis alba* Steud., Syn. Pl. Glum. 1: 248. 1855. Diplachne fusca (L.) Stapf var. alba (Steud.) Chiov., Fl. Somalia 1: 337. 1929. **Type.** SUDAN. Kordofan Mt., C.G.T. Kotschy 200 (holotype: Herb. Steudel, P [P00083376]!; isotypes: B!, BM! K, L! M! (2 sheets), MO! [MO-1742003] P! US! (US-899275, US-3298778) W! (2 sheets)). Many duplicates were given an incorrect designation of lectotype or isolectotypes in Snow (1997a).

*Uralepis drummondii* Steud., Syn. Pl. Glum. 1. 247. 1855. **Type.** AUSTRALIA, J. Drummond s.n. in Herbarium Drummond Coll. IV. s.n.; location unknown. Drummond's types are scattered across many herbaria, especially at K & MEL: e.g., https://www.anbg.gov.au/biography/drummond-james.html).

Names associated with Diplachne fusca subsp. fascicularis:

*Festuca clandestina* Muhl., Cat. Pl. Amer. Sept. 13: 1813; nom. nud.; Descr. Gram. 162. 1817. **Type.** United States of America, New York, N. Eboraco.

*Tridens virens* Nees, Fl. Bras. Enum. Pl. 2: 476. 1829. *Uralepis virens* (Nees) Kunth, Enum. Pl. 1: 319. 1833. *Diplachne virens* (Nees) Parodi, Rev. Fac. Agron. Vetrin. 6: 14. 1927. **Type.** BRAZIL. “Habitat in graminosis ad fluvim S. Francisci in provincia Bahiensi et Permambucana, ad Joazeiro et alibi". **Type.** Location unknown.

*Tridens veralensis* Cat. Guerra, Act. Bot. Cub. 4:4. 1980. **Type.** CUBA. Prov. de Pinar del Rio: Guanahacabibes, El Veral, Catasus 298 (holotype: HAC, n.v.).

Names associated with Diplachne fusca subsp. uninervia:

*Diplachne tarapacana* Phil., Verz. Antofagasta Pfl. 88. 1891. Lectotypification evidently is needed given that two specimens at SGO likely were in the original material (Rahmer s.n. and Philippi s.n.), although neither was cited in the protologue.

*Uralepis anderssonii* F. Aresch., Pl. Itin. Eugenia 119. 1910. **Type.** ECUADOR. Circa Guajaquil et in insula Puna, NJ Andersson 25 (types at S).

## In memorium

We dedicate this paper to Bryan Kenneth Simon (1943‒2015), our colleague, coauthor and friend, who regrettably did not live to see its completion (Snow 2015).

## Supplementary Material

XML Treatment for Diplachne

XML Treatment for Diplachne fusca

XML Treatment for Diplachne fusca subsp. fusca

XML Treatment for Diplachne fusca subsp. muelleri

XML Treatment for Diplachne fusca subsp. fascicularis

XML Treatment for Diplachne fusca subsp. uninervia

XML Treatment for Diplachne gigantea

## References

[B1] Abou-Shanab RA Ghozlan H Ghanem K Moawad H (2005) Behaviour of bacterial populations isolated from rhizosphere of *Diplachne fusca* dominant in industrial sites. World Journal of Microbiology and Biotechnology 21: 1095–1101. https://doi.org/10.1007/s11274-004-0005-6

[B2] Ahmad F (2009) *Leptochloa fusca* cultivation for utilization of salt-affected soil and water resources in Cholistan Desert. Sociedade e Natureza, Uberlândia 22: 141‒149. https://doi.org/10.1590/S1982-45132010000100010

[B3] Aliscioni S Bell HL Besnard G Christin P-A Columbus JT Duvall MR Edwards EJ Giussani L Hasenstab-Lehman K Hilu KW Hodkinson TR Ingram AL Kellogg EA Mashayekhi S Morrone O Osborne CP Salamin N Schaefer H Spriggs E Smith SA Zuloaga F (2012) New grass phylogeny resolves deep evolutionary relationships and discovers C4 origins. New Phytologist 193: 304–312. https://doi.org/10.1111/j.1469-8137.2011.03972.x 22115274 10.1111/j.1469-8137.2011.03972.x

[B4] Ashraf S Asfal M Naveed M Sahid M Zahir ZA (2017) Endophytic bacteria enhance remediation of tannery effluent in constructed wetlands with *Leptochloa fusca* International Journal of Phytoremediation. http://dx.doi.org/10.1080/15226514.2017.1337072 10.1080/15226514.2017.1337072 28621547

[B5] Auquier P Sommers Y (1967) Recherches histotaxonomiques sur le chaume des Poaceae. Bulletin of the Société royale de botanique de Belgique 100: 95–140.

[B6] Beadle NCW (1948a) Studies in wind erosion. III. Natural regeneration on scalded surfaces. Journal of Soil Conservation Service New South Wales 4: 123–134.

[B7] Beadle NCW (1948b) Studies in wind erosion. IV. Reclamation of scalds: general conseriderations. Conservation Service New South Wales 4: 160–170.

[B8] Bir SS Sahni M (1986) SOCGI plant chromosome number reports – IV. Journal of Cytology and Genetics 21: 152–154.

[B9] Breen CM Heeg J Seaman M (1993) South Africa. In: Whigham DF Dykyjová D Hejný S (Eds) Wetlands of the world I: inventory, ecology and management. Kluwer Academic Publishers, Dordrecht, 79–128. https://doi.org/10.1007/978-94-015-8212-4_4

[B10] Brown WV (1975) Variations in anatomy, associations, and origins of Kranz tissue. American Journal of Botany 62: 395–402. https://doi.org/10.2307/2442093

[B11] Brummitt RK (2001) World geographical scheme for recording plant distributions (2^nd^ edn). International Working Group on Taxonmic Databases (TDWG), XV, 153 pp.

[B12] Canfield RH (1934) Stem structure of grasses of the Jornada Experimental Range. Botanical gazette (London) 95: 636–648. https://doi.org/10.1086/334434

[B13] Catalán P Müller J (2012) *Festuca* L. – Flora Argentina: Flora Vascular de la República Argentina. Vol. 3, Tomo II, Monocotyledonae: Poaceae: Pooideae. Graficamente Ediciones, Córdoba, 219–250.

[B14] Chapman GP (1996) The Biology of Grasses. CAB International, Wallingford.

[B15] Clark LG Fisher JB (1987) Vegetative morphology of grasses: shoots and roots. In: Soderstrom TR, Hilu KW, Campbell CS, Barkworth ME (Eds) Grass systematics and evolution. Smithsonian Institution Press, Washington, D.C.

[B16] Clayton WD Renvoize SA (1986) Genera graminum: grasses of the world. Kew Bulletin Additional Series 13. Her Majesty's Stationery Office, London.

[B17] Clifford HT Watson L (1977) Identifying grasses. University of Queensland Press, St. Lucia.

[B18] Denny P (1993a) Wetlands of Africa: Introduction. In: Whigham DF Dykyjová D Hejny S (Eds) Wetlands of the World I: Inventory, Ecology, and Management. Kluwer Academic Publishers, Dordrecht, 1–28. https://doi.org/10.1007/978-94-015-8212-4_1

[B19] Denny P (1993b) Wetland use and conservation. In: Whigham DF Dykyjová D Hejny S (Eds) Wetlands of the World I: Inventory, Ecology, and Management. Kluwer Academic Publishers, Dordrecht, 31–46. https://doi.org/10.1007/978-94-015-8212-4_5

[B20] de Queiroz K (1998) The general lineage species concept of species, species criteria, and the process of speciation: A conceptual unification and terminological recommendations. In: Endless Forms: Species and Speciation. Oxford University Press, 57–75.

[B21] de Wet JMJ (1960) Culm anatomy in relation to taxonomy. Bothalia 7: 311–316. https://doi.org/10.4102/abc.v7i2.1662

[B22] Ebinger JE Carlen JL (1975) Culm morphology and grass systematics. Trans. Illinois State Acad. Sci. 68: 87–101.

[B23] Ellis RP (1976) A procedure for standardizing comparative leaf anatomy in the Poaceae. I. The leaf-blade as viewed in transverse section. Bothalia 12: 65–109. https://doi.org/10.4102/abc.v12i1.1382

[B24] Ellis RP (1977) Distribution of the Kranz syndrome in the southern African Eragrostoideae and Panicoideae according to the bundle sheath anatomy and cytology. Agroplantae 9: 73–110.

[B25] Gibbs Russell G Watson L Koekemoer M Smook L Barker NP Anderson HM Dallwitz MJ (1991) Grasses of Southern Africa. Revised edition. Memoirs of the Botanical Survey of South Africa No. 58.

[B26] Gleason HA (1968) The New Britton and Brown Illustrated Flora of the Northeastern United States and Adjacent Canada. Vol. 1. The Pteridophyta, Gymnospermae and Monocotyledonae. Hafner Publishing Co., New York.

[B27] Gleason HA Cronquist A (1991) Manual of Vascular Plants of Northeastern United States and Adjacent Canada. New York Botanical Garden, NY.

[B28] Gould FW (1958) Chromosome numbers in southwestern grasses. American Journal of Botany 45: 757–767. https://doi.org/10.2307/2439737

[B29] Gould FW (1966) Chromosome numbers in some Mexican grasses. Canadian Journal of Botany 44: 1683–1696. https://doi.org/10.1139/b66-181

[B30] Gould FW (1968) Chromosome numbers of Texas grasses. Canadian Journal of Botany 46: 1315–1325. https://doi.org/10.1139/b68-175

[B31] Gray A (1848) Manual of Botany of the Northeastern United States. J. Monroe, Boston.

[B32] Gray A (1857) Manual of Botany. Revised Edition. G.P. Putnam & Company, New York.

[B33] Hackel E (1887) Gramineae, Vol. 2, Pt. 2, In: Engler A, Prantl K (Eds) Die Naturlichen Pflanzenfamilien. W. Engelmann, Leipzig.

[B34] Hackel E (1900) Nachtrage zu Teil II, Abteilung 2. Gramimeae. In: Engler A, Prantl K (Eds) Prantl, Die Naturlichen Pflanzenfamilien. W. Engelmann. Leipzig.

[B35] Hackel E (1902) Neue gräser. Oesterreichische botanische Zeitschrift 52: 28–31. https://doi.org/10.1007/BF01790821

[B36] Hackel E (1906) Uber Kleistogamie bei den Gräsern. Oesterreichische botanische Zeitschrift 56: 81–88, 143–154, 180–186. https://doi.org/10.1007/BF01790904

[B37] Hackett C Wickens GE (1984) Plant description for practical purposes: A model profile of *Leptochloa fusca* (L.) Kunth, a salt-tolerant grass of arid lands. Technical Memorandum 84/27. CSIRO Institute of Biological Resources, Division of Land and Water Resources, Canberra.

[B38] Haq M Khan MFA (1971) Reclamation of saline and alkaline soils by growing kallar grass. The Nucleus 8: 139–144.

[B39] Hattersly PW Watson L (1976) C4 grasses: an anatomical criterion for distinguishing between NADP-Malic enzyme species and PCK or NAD-Malic enzyme species. Australian Journal of Botany 24: 297–308. https://doi.org/10.1071/BT9760297

[B40] Hitchcock AS (1951) Manual of the grasses of the United States. Second Edition. Revised by Agnes Chase. U.S. Department of Agriculture Miscellaneous Publication 200. Washington, DC. https://doi.org/10.5962/bhl.title.65332

[B41] Hurek T Reinhold B Fendrik I Niemann E-G (1987) Root-zone-specific oxygen tolerance of *Azospirillum* spp. and diazotrophic rods closely associated with Kallar Grass. Applied and Environmental Microbiology 53: 163–169. 16347257 10.1128/aem.53.1.163-169.1987 PMC203620

[B42] Hurek T Burggraf S Woese CR Reinhold-Hurek B (1993) 16S rRNA-targeted polymerase chain reaction and oligonucleotide hybridization to screen for *Azoarcus* spp., grass-associated diazotrophs. Applied and Environmental Microbiology 59: 3816–3824. 7506895 10.1128/aem.59.11.3816-3824.1993 PMC182536

[B43] Hurek T Reinhold-Hurek B van Montagu M Kellenberger E (1994) Root colonization and systemic spreading of *Azoarcus* sp. Strain BH72 in grasses. Journal of Bacteriology 176: 1913–1923. https://doi.org/10.1128/jb.176.7.1913-1923.1994 8144457 10.1128/jb.176.7.1913-1923.1994 PMC205294

[B44] Hurek T Egener T Reinhold-Hurek B (1997) Divergence in trogenases of *Azoarcus* spp., *Proteobacteria* of the β Subclass. Journal of Bacteriology 179: 4172–4178. https://doi.org/10.1128/jb.179.13.4172-4178.1997 9209030 10.1128/jb.179.13.4172-4178.1997 PMC179236

[B45] IUCN (2012) IUCN Red List Categories and Criteria: version 3.1 (2^nd^ edn). IUCN Species Survival Commission, Gland & Cambridge.

[B46] Jacobs SWL (1987) Systematics of the chloridoid grasses. In: Soderstrom TR Hilu KW Campbell CS Barkworth ME (Eds) Grass systematics and evolution. Smithsonian Institution Press, Washington, DC, 277–286.

[B47] Joshi YC Dwivedi RS Bal AR Qadar A (1983) Salt excretion by glands in *Diplachne fusca* (Linn.) P. Beauv. [sic]. Indian Journal of Plant Physiology 26: 203–208.

[B48] Kellogg EA (2015) XIII. Flowering Plants, Monocots, Poaceae. In: Kubitszki K (Ed.) The Families and Genera of Vascular Plants. Springer International, 1–416.

[B49] Kernick MD (1990) An assessment of grass succession, utilization and development in the arid zone. In: Chapman GP (Ed.) Reproductive Versatility of Grasses, Cambridge Univeristy Press, 154–181.

[B50] Koyama T (1987) Grasses of Japan and its Neighboring Regions: An Identification Manual. Kodansha, Tokyo.

[B51] Krause A Ramakumar A Bartels D Battistoni F Bekel T Boch J Böhm M Friedrich F Hurek T Krause L Linke B McHardy AC Sarkar A Schneiker S Syed AA Thauer R Vorhölter F-J Weidner S Pühler A Reinhold-Hurek B Kaiser O Goessman A (2006) Complete genome of the mutualistic, N_2_-fixing grass endophyte *Azoarcus* sp. strain BH72. Nature Biotechnology 24: https://doi.org/10.1038/nbt1243; corrigendum: http://www.nature.com/nbt/journal/v25/n4/full/nbt0407-478d.html 10.1038/nbt1243 17057704

[B52] Larsen A (1963) Studies in the flora of Thailand. 14. Cytological studies in vascular plants of Thailand. Danks Botanisk Arkiv 20: 211–275.

[B53] Lazarides M (1970) The grasses of central Australia. Australian National Univeristy Press, Canberra.

[B54] Lazarides M (1980) The genus *Leptochloa* Beauv. (Poaceae, Eragrostideae) in Australia and Papua New Guinea. Brunonia 3: 247–269. https://doi.org/10.1071/BRU9800247

[B55] Liu Q Nan-Xiang Z Hao G (2004) Pollen morphology of Chloridoideae (Gramineae). Grana 43: 238–248. https://doi.org/10.1080/00173130410000776

[B56] Linnaeus C (1762) Species Plantarum. Second edition. Laurentius Salvius, Stockholm.

[B57] Mauz K (2011) Cyrus Pringle's vascular plant types from western United States and Mexico, 1881–1884. Harvard Papers in Botany 16: 71–141. https://doi.org/10.3100/025.016.0111

[B58] McIntyre S Barrett SCH (1985) A comparison of weed communities of rice in Australia and California. Proceedings of the Ecological Society of Australia 14: 237–250.

[B59] McIntyre S Mitchell S Ladiges PY (1989) Germination and seedling emergence in *Diplachne fusca*: a semi-aquatic weed of rice fields. Journal of Applied Ecology 26: 551–562. https://doi.org/10.2307/2404080

[B60] McNeill J (1979) *Diplachne* and *Leptochloa* in North America. Brittonia 31: 399–404. https://doi.org/10.2307/2806134

[B61] McVaugh R (1983) Gramineae, Vol. 14. Flora Novo-Galiciana: A descriptive account of the vascular plants of western Mexico. The University of Michigan Press, Ann Arbor.

[B62] Myers BA Morgan WC (1989) Germination of the salt-tolerant grass *Diplachne fusca*. II. Salinity responses. Australian Journal of Botany 37: 225–237. https://doi.org/10.1071/BT9890225

[B63] Nicora EG (1995) Los géneros *Diplachne* y *Leptochloa* (Gramineae, Eragrosteae) de la Argentina y países limítrofes. Darwiniana 33: 233–256.

[B64] Nicora EG (2006) 2. *Diplachne* P. Veauv. In: Molina AM Rúgolo de Agrasar ZE (Eds) Flora Chaqueña – Argentina: familia Gramíneas. Colección Científica del Instituto Nacional de Tecnología Acropecuaria 23: 263–272.

[B65] Palisot de Beauvois AMFJ (1812) Essai d'une Nouvelle Agrostographie; ou Noveaux Genres des Gramineés; avec Figures Représentant les Caractères de Tous les Genres. Fain, Paris.

[B66] Parodi LR (1927) Revisión de las gramíneas argentinas del género *Diplachne*. Revista Fac. Agron. Veterin. 6: 21–43.

[B67] Pérez C J L Show N Sánchez GE (2010) Comentario sobre el género *Leptochloa* P. Beauv. (Poaceae, Eragrostideae), en Extremadura y Andalucía (España). Acta Botanica Malacitana 35: 169–172.

[B68] Peterson PM Soreng RJ Davidse G Filgueiras TS Zuloaga FO Judziewicz EJ (2001) Catalogue of New World grasses (Poaceae): II. Subfamily Chloridoideae. Contributions from the United States National Herbarum 41: 1–255.

[B69] Peterson PM Romaschenko K Johnson G (2010) A classification of the Chloridoideae (Poaceae) based on multi-gene phylogenetic trees. Molecular Phylogenetics and Evolution 55: 580–598. https://doi.org/10.1016/j.ympev.2010.01.018 20096795 10.1016/j.ympev.2010.01.018

[B70] Peterson PM Romaschenko K Snow N Johnson G (2012) A molecular phylogeny and classification of *Leptochloa* (Poaceae: Chloridoideae: Chlorideae) *sensu lato* and related genera. Annals of Botany 109: 1319–1327. https://doi.org/10.1093/aob/mcs077 10.1093/aob/mcs077 PMC3359928 22628365

[B71] Peterson PM Romaschenko K Soreng RJ (2014) Invited Paper: A laboratory guide for generating DNA barcodes in grasses: a case study of *Leptochloa* s.l. (Poaceae, Chloridoideae). Webbia: Journal of Plant Taxonomy and Geography 69: 1–12. http://dx.doi.org/10.1080/00837792.2014.927555

[B72] Peterson PM Romaschenko K Herrera Arieta Y (2015) A molecular phylogeny and classification of the Eleusininae with a new genus, *Microachne* (Poaceae: Chloridoideae: Cynodonteae). Taxon 64: 445–467. https://doi.org/10.12705/643.5

[B73] Peterson PM Romaschenko K Herrera Arieta Y (2016) A molecular phylogeny and classification of the Cynodonteae (Poaceae: Chloridoideae). Taxon 65: 1263–1287. https://doi.org/10.12705/656.4

[B74] Peterson PM Romaschenko K Herrera Arrieta Y (2017) Four new subtribes: Allolepiinae, Jouveinae, Kaliniinae, and Sohnsiinae in the Cynodonteae (Poaceae: Chloridoideae). Phytoneuron 2017-44: 1–9. http://www.phytoneuron.net/2017Phytoneuron/44PhytoN-ChloridoidSubtribes.pdf

[B75] Phillips SM (1974) Studies in the Gramineae. XXXV. Kew Bulletin 29: 267–270. https://doi.org/10.2307/4108540

[B76] Phillips SM (1982) A numerical analysis of Eragrostideae (Gramineae). Kew Bulletin 37: 133–162. https://doi.org/10.2307/4114722

[B77] Pohl RW Lersten NR (1975) Stem aerenchyma as a character separating *Hymenachne* and *Sacciolepis* (Gramineae, Panicoideae). Brittonia 27: 223–227. https://doi.org/10.2307/2805893

[B78] Reeder JR (1957) The embryo in grass systematics. American Journal of Botany 44: 756–768. https://doi.org/10.2307/2438397

[B79] Reeder JR (1961) The embryo in grass systematics. Recent Advances in Botany. Vol. 1. University of Toronto Press, Toronto, 91–96.

[B80] Reeder JR (1971) Notes on Mexican grasses IX. Miscellaneous chromosome numbers - 3. Brittonia 23: 105–117. https://doi.org/10.2307/2805426

[B81] Reinhold-Hurek B Hurek T (1997) *Azoarcus* spp. and their interactions with grass roots. Plant and Soil 194: 57–64. https://doi.org/10.1023/A:1004216507507

[B82] Reinhold B Hurek T Niemann E-G Fendrik I (1986) Close association of *Azospirillum* and diazotrophic rods with different root zones of kallar grass. Applied and Environmental Microbiology 52: 520–526. 16347149 10.1128/aem.52.3.520-526.1986 PMC203566

[B83] Reinhold-Hurek B Hurek T Gillis M Hoste B Vancanneyt N Kersters K de Ley J (1993) *Azoarcus* gen. nov., nitrogen-fixing proteobacteria associated with roots of kallar grass (*Leptochloa fusca* (L.) Kunth), and description of two new species, *Azoarcus indigens* sp. nov. and *A. communis*. International Journal of Systematic Bacteriology 43: 574–584. https://doi.org/10.1099/00207713-43-3-574

[B84] Renvoize SA (1984) The grasses of Bahia. Royal Botanic Gardens, Kew.

[B85] Retzius AJ (1789) Observationes botanicae. Sigfried Lebrecht Crusium, Leipzig.

[B86] Sánchez E (1979) Estructura Kranz en tallos de Gramineae (Eragrosteae). Kurtziana 12–13: 113–118.

[B87] Sánchez E (1981a) Desarollo de la estructura Kranz en tallos de Gramineae. Lilloa 35: 37–40.

[B88] Sánchez E (1981b) Variación de la estructura Kranz en el tallo de *Diandrochloa glomerata* (Walter) Burkart. Lilloa 35: 41–6.

[B89] Sánchez E Arriaga MO Ellis RP (1989) Kranz distinctive cells in the culm of *Arundinella* (Arundinelleae; Panicoideae; Poaceae). Bothalia 19: 45–52.

[B90] Sandhu GR Aslam Z Sattar A Gureshi RH Ahmad N Wyn Jones RG (1981) The effect of salinity on the yield and composition of *Diplachne fusca* (kallar grass) plant. Cell Environ 9: 171–181.

[B91] Scholz H Böcker R (1996) Ergänzungen und Anmerkungen zur Grasflora (Poaceae) der Kanaren. Willdenowia 25: 571–582.

[B92] Shivas RG Vánky K (2003) Biodiveristy of Australian smut fungi. Fungal Diversity 13: 137–152.

[B93] Snow N (1996) The phylogenetic utility of lemmatal micromorphological characters in *Leptochloa* and related genera in subtribe Eleusininae (Poaceae, Chloridoideae, Eragrostideae). Annals of the Missouri Botanical Garden 83: 504–529. https://doi.org/10.2307/2399991

[B94] Snow N (1997a) Phylogeny and systematics of *Leptochloa* P. Beauv. sensu lato (Poaceae, Chloridoideae, Eragrostideae). Ph.D. dissertation, Washington University in St. Louis: Missouri.

[B95] Snow N (1997b) Application of the phylogenetic species concept: A botanical monographic perspective. Austrobaileya 5: 1–8.

[B96] Snow N (1998b) Caryopsis morphology of *Leptochloa* sensu lato (Poaceae, Chloridoideae). Sida 18: 271–282.

[B97] Snow N (2000) *Festuca reptatrix* L. In: Cafferty S Jarvis CE Turland NJ (Eds) Typification of Linnaean plant names in the Poaceae (Gramineae). Taxon 49, 250–251.

[B98] Snow N (2003) *Leptochloa* P. Beauv. Flora of North America. Vol. 25. Oxford University Press, 51–60.

[B99] Snow N (2012) *Leptochloa* P. Beauv. In: Zuloaga FO Rúgolo ZE Anton AM (Eds) Flora Argentina, Vol. 3, Tomo 2. Graficamente Ediciones, Córdoba, 131–138.

[B100] Snow N (2015) In memory of Bryan K. Simon: friend and colleague. Australian Systematic Botany Society Newsletter 161: 50–51.

[B101] Snow N Davidse G (1998) (1330) Proposal to reject the name *Poa malabarica* (Gramineae). Taxon 47: 157–159. https://doi.org/10.2307/1224034

[B102] Snow N Simon BK (1999) Taxonomic status and Australian distribution of of the weedy neotropical grass Leptochloa fusca subsp. uninervia, with an updated key to Australian *Leptochloa* (Poaceae, Chloridoideae). Austrobaileya 5: 299–305.

[B103] Snow N Peterson PM (2012a) Systematics of *Trigonochloa* (Poaceae, Chloridoideae, Chlorideae). PhytoKeys 13: 25–38. https://doi.org/10.3897/phytokeys.13/3355 10.3897/phytokeys.13.3355 PMC3391715 22787425

[B104] Snow N Peterson PM (2012b) Nomenclatural notes on *Dinebra*, *Diplachne*, *Disakisperma*, and *Leptochloa* (Poaceae: Chloridoideae). Phytoneuron 71: 1–2. http://www.phytoneuron.net/PhytoN-Leptochloa.pdf

[B105] Snow N Peterson PM (2013) Systematics of *Disakisperma* (Poaceae, Chloridoideae, Chlorideae). PhytoKeys 26: 21–76. https://doi.org/10.3897/phytokeys.26.5649 10.3897/phytokeys.26.5649 PMC3817421 24194669

[B106] Snow N Guymer GP Sawvel G (2003) Systematics of *Austromyrtus*, *Lenwebbia*, and the Australian species of *Gossia* (Myrtaceae). Systematic Botany Monographs 65: 1–95. https://doi.org/10.2307/25027907

[B107] Snow N Peterson PM Giraldo-Cañas D (2008) *Leptochloa* (Poaceae: Chloridoideae) in Colombia. Journal of the Botanical Research Institute of Texas 2: 861–874.

[B108] Soreng RJ Davidse G Peterson PM Zuloaga FO Judziewicz EJ Filgueiras TS Morrone O Romaschenko K (2012) A World-wide Phylogenetic Classification of Poaceae (Gramineae): capim, çimen, çayir, darbha, ghaas, ghas, gramas, gräser, grasses, he ben ke, hullu, kasa, kusa, pastos, pillu, pullu, zlaki, etc. http:/www.tropicos.org/projectwebportal.aspx?pagename=ClassificationNWG&projectid=10

[B109] Soreng RJ Peterson PM Romaschenko K Davidse G Zuloaga FO Judziewicz EJ Filgueiras TS Davis JI Morrone O (2015) Aworldwide phylogenetic classiﬁcation of the Poaceae (Gramineae). Journal of Systematics and Evolution 53: 117–137. https://doi.org/10.1111/jse.12150

[B110] Soreng RJ Peterson P Romaschenko K Davidse G Teisher J Clark L Barbera P Gillespie L Zuloaga F (2017) A worldwide phylogenetic classification of the Poaceae (Gramineae) II: An update and comparison of two 2015 classifications. Journal of Systematics and Evolution 55: 259−290. https://doi.org/10.1111/jse.12262

[B111] Spies JJ Vouges SP (1988) Chrosome studies of African plants – 7. Bothalia 18: 114–119.

[B112] Spies JJ Van der Merwe E Du Plessis H Saayman EJL (1991) Basic chromosome numbers and polyploid levels in some South African and Australian grasses (Poaceae). Bothalia 1: 163–170. https://doi.org/10.4102/abc.v21i2.882

[B113] Steudel EG (1855) Synopsis Plantarum Glumacearum. Pars 2.

[B114] Sutton DD (1973a) Leaf anatomy in *Leptochloa fascicularias* [sic]. Michigan Academician 5: 113–119.

[B115] Sutton DD (1973b) Leaf anatomy in the subfamily Eragrostoideae. Michigan Academician 5: 373–383.

[B116] Systematics Association Committee for Descriptive Biological Terminology (1962) II. Terminology of simple symmetrical plane shapes (Chart 1). Taxon 11: 145–156. https://doi.org/10.2307/1216718

[B117] Thiers B (2017) Index Herbariorum: A global directory of public herbaria and associated staff. New York Botanical Garden's Virtual Herbarium. http://sweetgum.nybg.org/ih [and continuously updated]

[B118] Valls JFM (1978) A biosystematic study of *Leptochloa* with special emphasis on *Leptochloa dubia* (Gramineae: Chloridoideae). PhD Dissertation, Texas A&M University, College Station.

[B119] Watson L Dallwitz M (1992) The Grass Genera of the World. CAB International, Wallingford.

